# Discovery of Novel Chemical Series of OXA-48 β-Lactamase Inhibitors by High-Throughput Screening

**DOI:** 10.3390/ph14070612

**Published:** 2021-06-25

**Authors:** Barbara Garofalo, Federica Prati, Rosa Buonfiglio, Isabella Coletta, Noemi D’Atanasio, Angela Molteni, Daniele Carettoni, Valeria Wanke, Giorgio Pochetti, Roberta Montanari, Davide Capelli, Claudio Milanese, Francesco Paolo Di Giorgio, Rosella Ombrato

**Affiliations:** 1 Angelini Pharma S.p.A., Global R&D External Innovation, Viale Amelia 70, 00181 Rome, Italy; barbara.garofalo@angelinipharma.com (B.G.); federica.prati@angelinipharma.com (F.P.); rosa.buonfiglio@angelinipharma.com (R.B.); isabella.coletta@angelinipharma.com (I.C.); noemi.datanasio@angelinipharma.com (N.D.); claudio.milanese@angelinipharma.com (C.M.); francescopaolo.digiorgio@angelinipharma.com (F.P.D.G.); 2 Axxam SpA Via Meucci 3, Bresso, 20091 Milan, Italy; Angela.Molteni.AM@axxam.com (A.M.); Daniele.Carettoni.DC@axxam.com (D.C.); Valeria.Wanke.VW@axxam.com (V.W.); 3 Consiglio Nazionale delle Ricerche—Istituto di Cristallografia, Via Salaria—km 29.300, Monterotondo, 00015 Rome, Italy; giorgio.pochetti@ic.cnr.it (G.P.); roberta.montanari@ic.cnr.it (R.M.); davide.capelli@ic.cnr.it (D.C.)

**Keywords:** OXA-48, β-lactamase inhibitor, bacterial resistance, HTS

## Abstract

The major cause of bacterial resistance to β-lactams is the production of hydrolytic β-lactamase enzymes. Nowadays, the combination of β-lactam antibiotics with β-lactamase inhibitors (BLIs) is the main strategy for overcoming such issues. Nevertheless, particularly challenging β-lactamases, such as OXA-48, pose the need for novel and effective treatments. Herein, we describe the screening of a proprietary compound collection against *Klebsiella pneumoniae* OXA-48, leading to the identification of several chemotypes, like the 4-ideneamino-4H-1,2,4-triazole (SC_2) and pyrazolo[3,4-b]pyridine (SC_7) cores as potential inhibitors. Importantly, the most potent representative of the latter series (ID2, AC_50_ = 0.99 μM) inhibited OXA-48 via a reversible and competitive mechanism of action, as demonstrated by biochemical and X-ray studies; furthermore, it slightly improved imipenem’s activity in *Escherichia coli* ATCC BAA-2523 β-lactam resistant strain. Also, **ID2** showed good solubility and no sign of toxicity up to the highest tested concentration, resulting in a promising starting point for further optimization programs toward novel and effective non-β-lactam BLIs.

## 1. Introduction

β-lactams such as penicillins, cephalosporins, carbapenems, and monobactams are the most effective and extensively used class of antibacterial agents, representing approximately 65% of the prescribed antibiotics worldwide [1]. Unfortunately, the widespread use of these medicaments has culminated in the outbreak and dissemination of resistance mechanisms to β-lactams. Prevalently, drug-resistant bacteria produce β-lactamase enzymes that hydrolyze and inactivate the β-lactam ring [2,3,4,5], thus preventing the covalent inhibition of essential cell-wall biosynthesis proteins, named penicillin-binding proteins (PBPs) [6,7,8,9,10], which result in the loss of drug’s antibacterial effectiveness.

Today, a high number of β-lactamase variants are known (>7000 to date) [11], and organized in A, B, C, and D classes (Ambler classification) on the basis of their amino acid sequence homology [12,13]. β-lactamases are also distinguished as serine-dependent (Ambler classes A, C, and D) or metallo (Ambler class B) enzymes, according to the mechanism driving the β-lactam hydrolysis [12,13].

**Figure 1:**
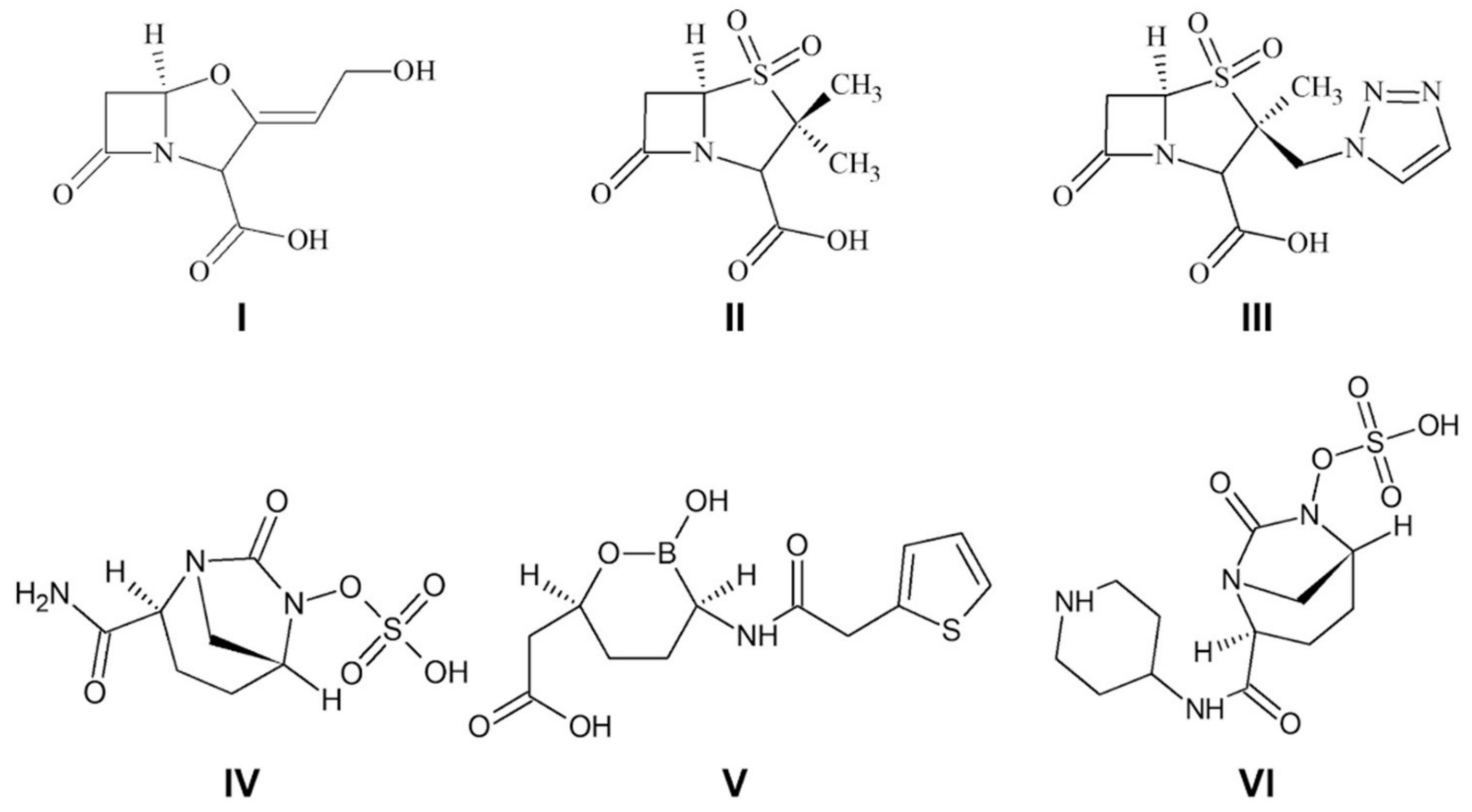
Structure of clinically available BLIs: clavulanic acid (**I**), sulbactam (**II**), tazobactam (**III**), avibactam (**IV**), vaborbactam (**V**), relebactam (**VI**).

Nowadays, the combination of susceptible β-lactams with β-lactamase inhibitors (BLIs), which protect them from degradation and restore their antibacterial activity, serves as the major strategy for overcoming the antibiotic resistance [6]. In this respect, clavulanic acid, sulbactam, and tazobactam (compounds **I**–**III** in Figure 1, respectively) were the first BLIs available to the healthcare community. All the compounds act as suicide substrates through the formation of irreversible adducts with β-lactamases. Clavulanic acid and tazobactam are mainly active against class A enzymes with limited clinical utility towards contemporary isolates [6,14,15], whereas sulbactam displaying less activity against class A β-lactamases is more effective against class C β-lactamases [14]. Over the last years, three new BLI combination products with greater spectra of inhibition were approved: avibactam with ceftazidime [16], vaborbactam with meropenem [17], and relebactam with imipenem (IMP)-cilastatin [18,19] (compounds **IV**–**VI** in Figure 1). These novel drugs operate via a covalent mechanism of action (MoA). However, differently from the first-generation BLIs, they do not share the same labile 2-azetidone-based structure of β-lactam antibiotics, thus avoiding a critical factor in the rapid evolution of resistance [15,20,21].

Interestingly, avibactam is also the only clinically available BLI with significant efficacy against OXA-48 enzyme [22]. OXA-48 belongs to the carbapenem-hydrolyzing class D β-lactamases (CHDLs) and together with its variants represents one of the most severe threat of resistance, compromising efficacious anti-infective treatments. Isolated for the first time in 2001 from a patient in Turkey [23], the OXA-48 enzyme is now widespread across the world and is commonly identified in *Escherichia coli* and *Klebsiella pneumoniae*. Unfortunately, resistance to ceftazidime-avibactam for class A and D β-lactamases have been recently reported [24,25,26,27,28]. Hence, novel OXA-48 inhibitors with reduced susceptibility to bacterial resistance mechanisms are foreseen to come. Accordingly, following the development of non-β-lactam BLIs, new research efforts led to non-covalent BLIs as alternatives [6,29,30,31]. The development of new OXA-48 inhibitors with a different enzyme-inhibition profile compared to existing inhibitors is of importance since new mechanisms for BLI inactivation would be likely required [32].

On these premises, we embraced a drug discovery campaign towards novel OXA-48 inhibitors structurally different from previously identified BLIs. Herein, we report the high-throughput screening (HTS) of an internal compound collection, against the OXA-48 enzyme, and thoroughly describe the analyses leading to the identification of several scaffold families as potential inhibitors. Particularly, the 4-ideneamino-4*H*-1,2,4-triazole and pyrazolo[3,4-b]pyridine chemical series were further investigated in terms of preliminary structure activity relationship (SAR) analysis, mechanism of action (MoA) determination by means of biochemical and X-ray studies, whole-cell activity in combination with representative β-lactams against different bacterial strains. Importantly, one representative (**ID2**) of the pyrazolo[3,4-b]pyridine family resulted effective in restoring β-lactam activity in resistant-bacteria via a reversible MoA and showed favorable drug-like properties.

## 2. Results and Discussion

### 2.1. Compound Collection

A fundamental requirement for the success of any HTS is the availability of a sizeable and properly organized chemical library. Here, a well-organized and diversified chemical collection was built from a pool of commercially available compounds. The chemical library was compiled by employing the scaffold concept to aid preliminary SAR expansion after HTS data analysis, besides ensuring chemical variability of the dataset. Starting from the compound catalogs of several vendors, millions of molecules were filtered out due to physicochemical properties out of conventional drug-like ranges [33] and structural alerts as reactive groups [34], REOS [35], and PAINS [36,37]. The remaining compounds were submitted to framework decomposition based on the Bemis-Murcko definition [38], providing ~8600 decorated scaffolds consisting of a central core and appended groups (unsubstituted rings and chains). Each scaffold included an average of 20 compounds up to a maximum number of 54 representatives. Also, molecules were annotated using the InChyKey [39] of the corresponding scaffold family to facilitate database storage and searching. Overall, ~190,000 molecules were collected. We reasoned that this number of compounds was a good compromise to contain molecular redundancy but guarantee enough cluster representativeness to efficiently identify latent hit series, rapidly define preliminary SAR, and avoid singleton clusters [40,41].

The compound library was assembled in a modular fashion, consisting of two non-redundant sets: (i) full set composed of 156918 compounds, and (ii) subset containing 34423 representatives. Each scaffold was described from one to four molecules in the subset and up to 50 molecules in the full set.

The rationale behind this library assembling was the opportunity of: (i) screening the subset only in the first step of a HTS, thus minimizing the number of tested compounds while maintaining high chemical diversity; (ii) running a fast follow-up for SAR analysis and expansion of hits at minimum time and cost, by simply selecting classes of active analogs from the full set.

**Figure 2:**
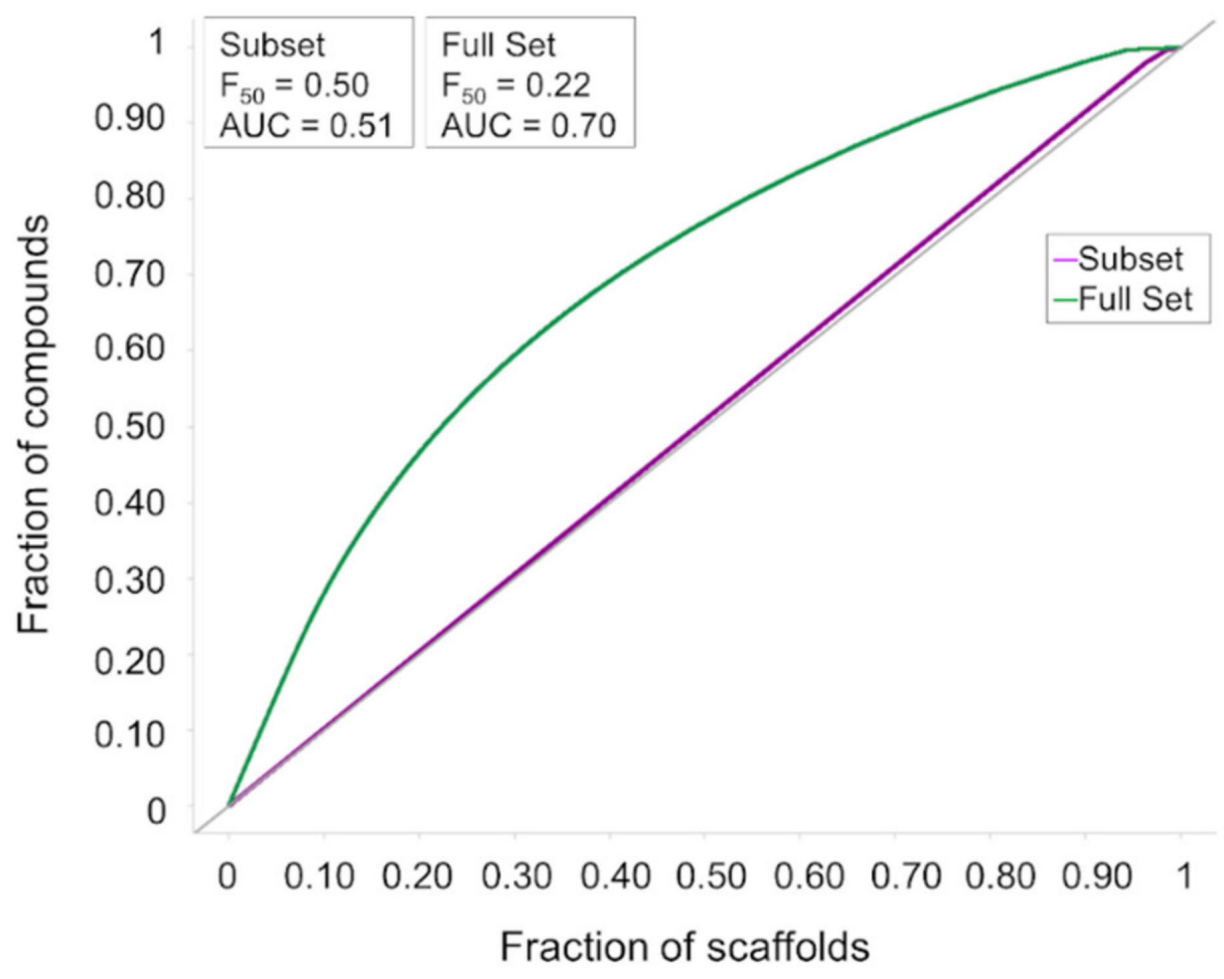
Cyclic system retrieval (CSR) curves of the subset and full set. Related AUC and F_50_ values are also reported.

The scaffold distribution along the two datasets can be illustrated by the cyclic system retrieval (CSR) curves (Figure 2) [42,43,44], where the cumulative fraction of compounds (Y-axis) per dataset is plotted as a function of the corresponding fraction of scaffolds (X-axis).

The maximum diversity arises when a dataset is composed of one chemotype per molecule. In this case, the CSR curve corresponds to a diagonal. As the scaffold diversity decreases, the curve moves away from the diagonal, becoming a vertical line if all the compounds have the same chemotype. As depicted in Figure 2, the curve of the Subset (violet line) is close to the diagonal indicating a good diversity, while the curved green line of the full set highlights that it contains more densely represented scaffolds. Starting from the CSR curves, related metrics, such as the area under the curve (AUC) and the fraction of scaffolds to retrieve 50% of the dataset (F_50_), can be also used to quantitatively compare the two datasets [45,46]. Low AUC values mean high scaffold diversity, while the opposite is true for F50 values. Consistent with the shape of the curves, F_50_ and AUC values of the subset are 0.50 and 0.51, respectively. Conversely, the Full Set shows the largest AUC value of 0.70 and F_50_ is equal to 0.22. Overall, these data indicate that the subset encompasses a high chemical diversity and support the rationale for testing this dataset as representative of the full set in the first stage of an HTS campaign.

### 2.2. High-Throughput Screening

**Figure 3:**
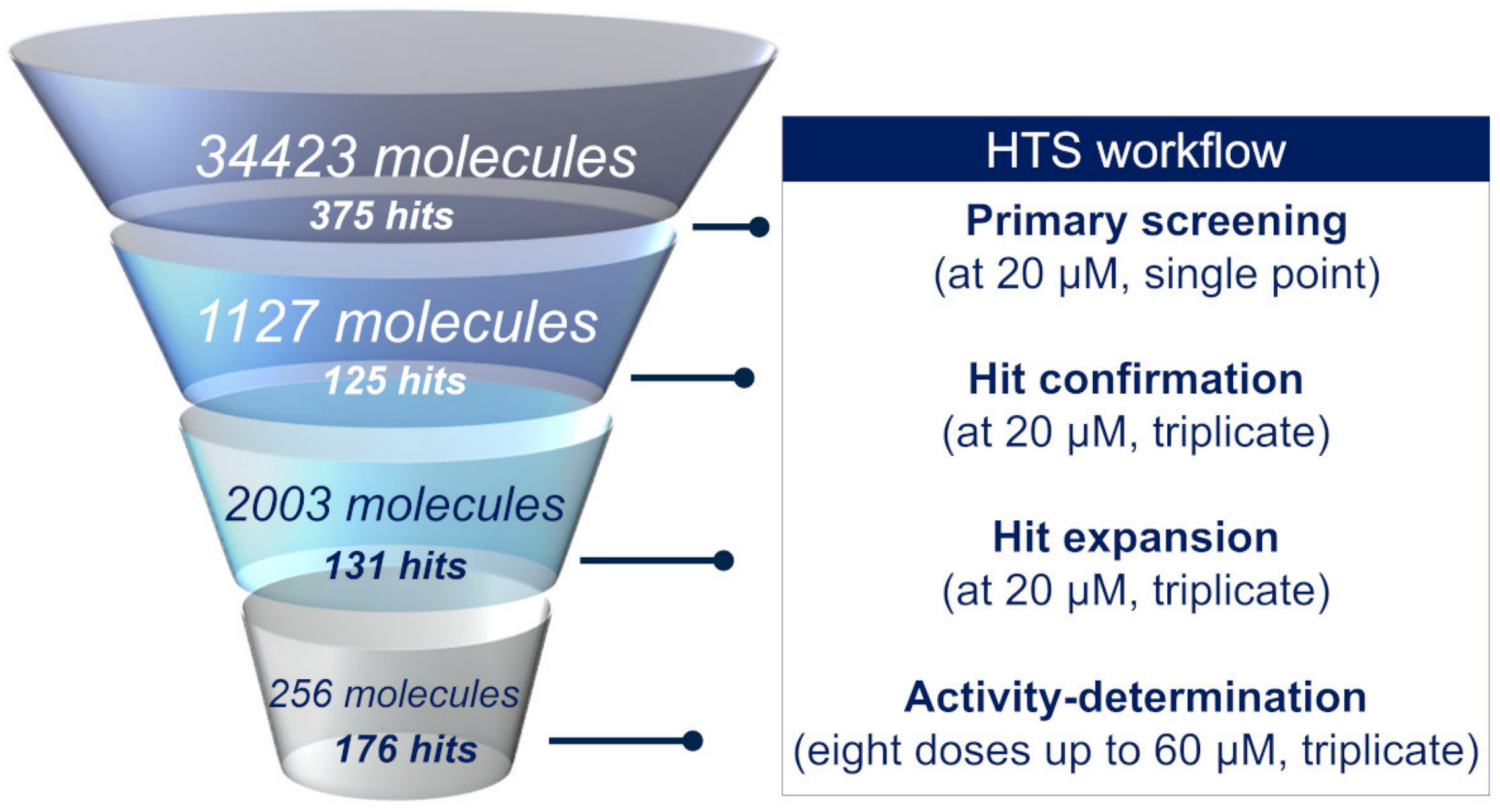
HTS workflow.

During the last decade, HTS has evolved to become an innovative multidisciplinary branch in biological chemistry combining aspects of medicinal chemistry, biology, and laboratory automation. A typical HTS campaign can be divided into four main phases, regardless of the assay type and readout: (i) primary screening, (ii) hit confirmation (HC), (iii) hit expansion (HE), and (iv) activity determination [47]. In the next paragraphs, we discuss the results of a HTS campaign against *K. pneumoniae* OXA-48 enzyme starting from the propriety library described above (Figure 3).

#### 2.2.1. Primary Screening

After an initial pilot run (three plates, consisting of 960 randomly selected compounds), the primary screening of the subset plates was carried out at 20 µM in single point against OXA-48 enzyme in a nitrocefin-based assay, using the fluorescence-quenching readout. This enzymatic assay employs the cephalosporin nitrocefin as OXA-48 substrate and takes advantage of the fluorescent properties of white microtiter plates. Chromogenic nitrocefin is hydrolyzed by β-lactamases to yield a product with increased absorbance properties (~495 nm) that quenches the plate fluorescence by absorbing the plate’s emission light [48]. In this assay, test compounds that inhibit OXA-48 catalytic activity are predicted to reduce nitrocefin quenching of plate fluorescence, resulting in a quantitative increase in well fluorescence (ON-system for inhibitors). The nitrocefin-based epi-absorbance readout system has been successfully applied in HTS campaigns on other β-lactamases, such as IMP-1 and VIM-2, with the identification of inhibitors belonging to novel scaffolds displaying a favourable degree of selectivity and sub-micromolar potency [49,50].

The screening results were expressed as activity percent. Considering that the activity of the neutral control was set at 0%, enzyme inhibitors and stimulators showed negative and positive values, respectively. Consistently, compounds causing a complete inhibition of the target signal resulted in an activity percent equivalent to −100%. Overall, the primary assay robustness was good [51], with Robust Z Prime factor (RZ′) equal to 0.86.

**Figure 4:**
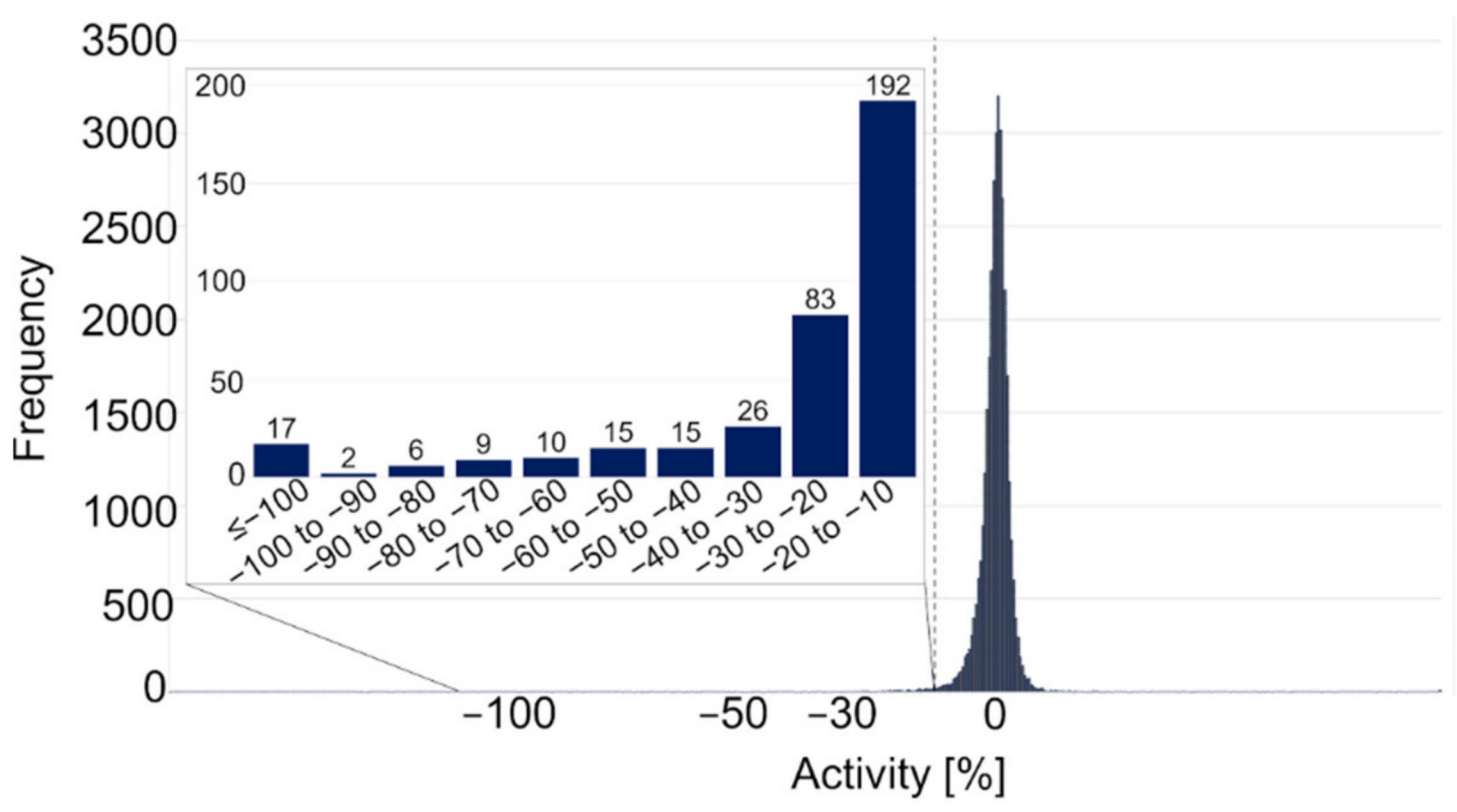
Bar chart showing the compound distribution across the activity percent as outcome of the primary screening. A zoom of the distribution of the negative values are also reported. The activity binning was performed considering half-closed intervals in which the largest endpoint was included. The dashed line indicates the activity threshold.

Figure 4 displays the distribution of tested compounds as function of the activity percent, showing a Gaussian-like curve. Around the mean value of −0.13%, the negative X-axis is differently populated in respect to the right part of the graph with a gentle decrease of frequency moving down to −100% activity (long and thin tail in Figure 4), thus leading to an asymmetric distribution. Interestingly, such left-skewed curve highlights the presence of a certain number of compounds that potentially induce a complete inhibition of the target (zoomed bar chart in Figure 4).

In this study, we set the activity threshold for hit selection at −12.60% (dashed line in Figure 4), which corresponds to the average value plus two times the standard deviation. Accordingly, we identified 375 putative hits out of 34423 tested compounds (1.1% hit rate), which were distributed to different extent across 295 InChyKey scaffolds.

#### 2.2.2. Hit Confirmation

The 375 putative hits and the corresponding inactive analogs selected from the same clusters in the Subset plates (752 compounds) were pursued to HC and tested at 20 µM with interplate triplicates. Overall, we identified 299 OXA-48 inhibitors (activity below −12.60%) out of 1127 retested compounds.

**Figure 5:**
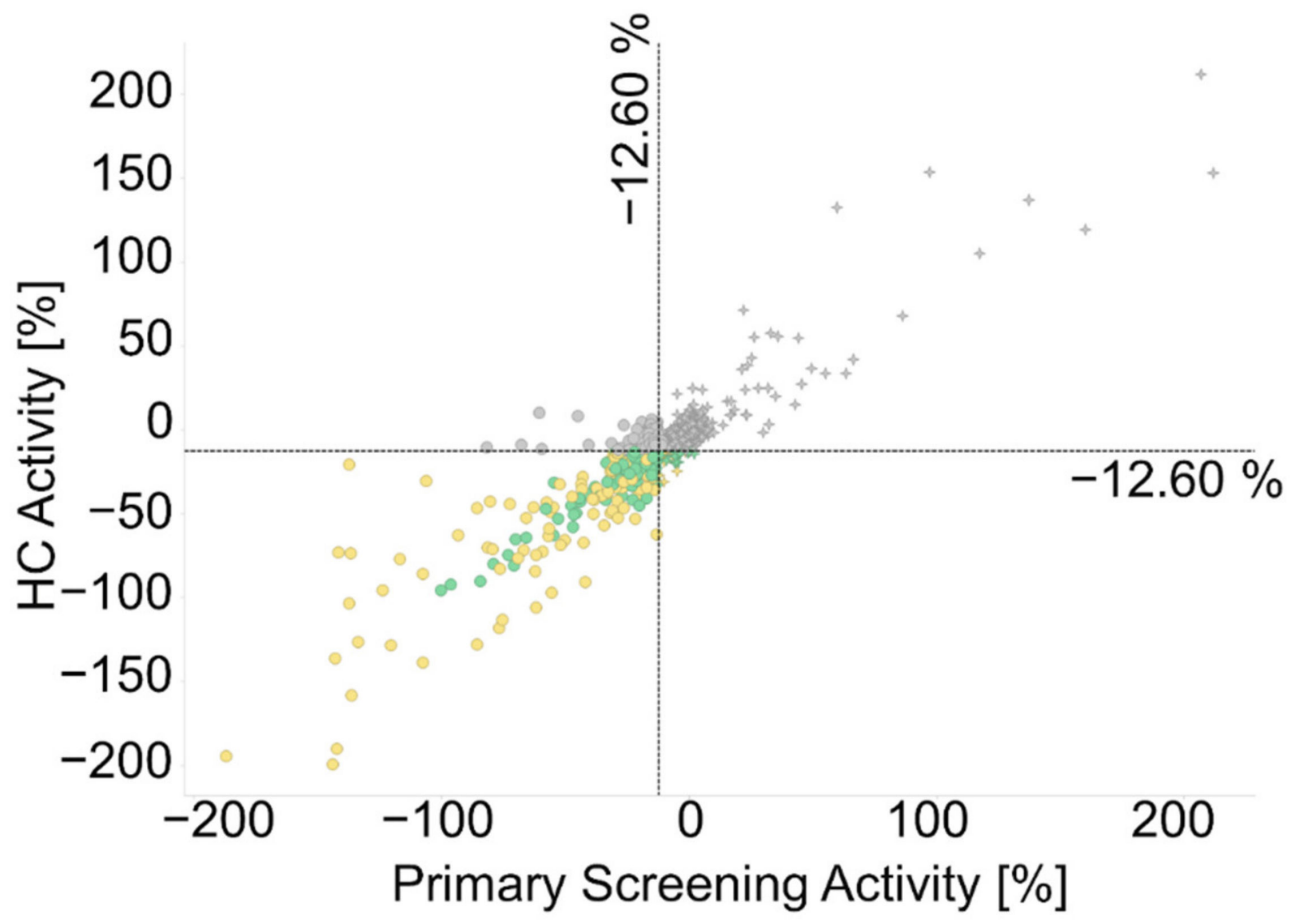
Scatter plot of primary screening versus HC in terms of activity percent. The dots are shaped by outcome of the primary screening: circles and diamonds correspond to actives and inactives, respectively. The dots are colored by outcome of the HC: green, yellow, and grey correspond to active, interfering, and inactive compounds, respectively.

Specifically, 264 hits confirmed the primary screening results (dots in the left bottom panel in Figure 5), with a confirmation rate of 70.4%. The remaining 35 actives were recovered from the compound set that showed no efficacy in the primary assay (diamonds in the right bottom panel in Figure 5). Overall, HC data brought out the good reproducibility of the applied screening test. Indeed, the highly potent primary hits were also active in HC, whereas compounds close to the activity threshold could be differently classified as actives or inactives in the two screening steps.

The 299 confirmed hits were then checked for potential interfering behavior with the assay readout, to possibly identify and discard false positives. Specifically, the tested compounds were added (20 µM, triplicate) to the assay well after the fluorescence reaction between OXA-48 enzyme and the chromogenic nitrocefin substrate was completed to check on potential autofluorescence effects. Compounds were tagged as assay interferents if the difference in the enzymatic activity before and after their administration was below −15% (this cut-off was computed as the average of values plus three times the standard deviation). Overall, 161 out of 299 compounds resulted true hits, with a confirmation rate of 53.9%. (Figure 5). Interestingly, all compounds with activity percent below −100% resulted false positives, whereas the true hits were mostly distributed near the −12.60% HC threshold (Figure 5).

**Figure 6:**
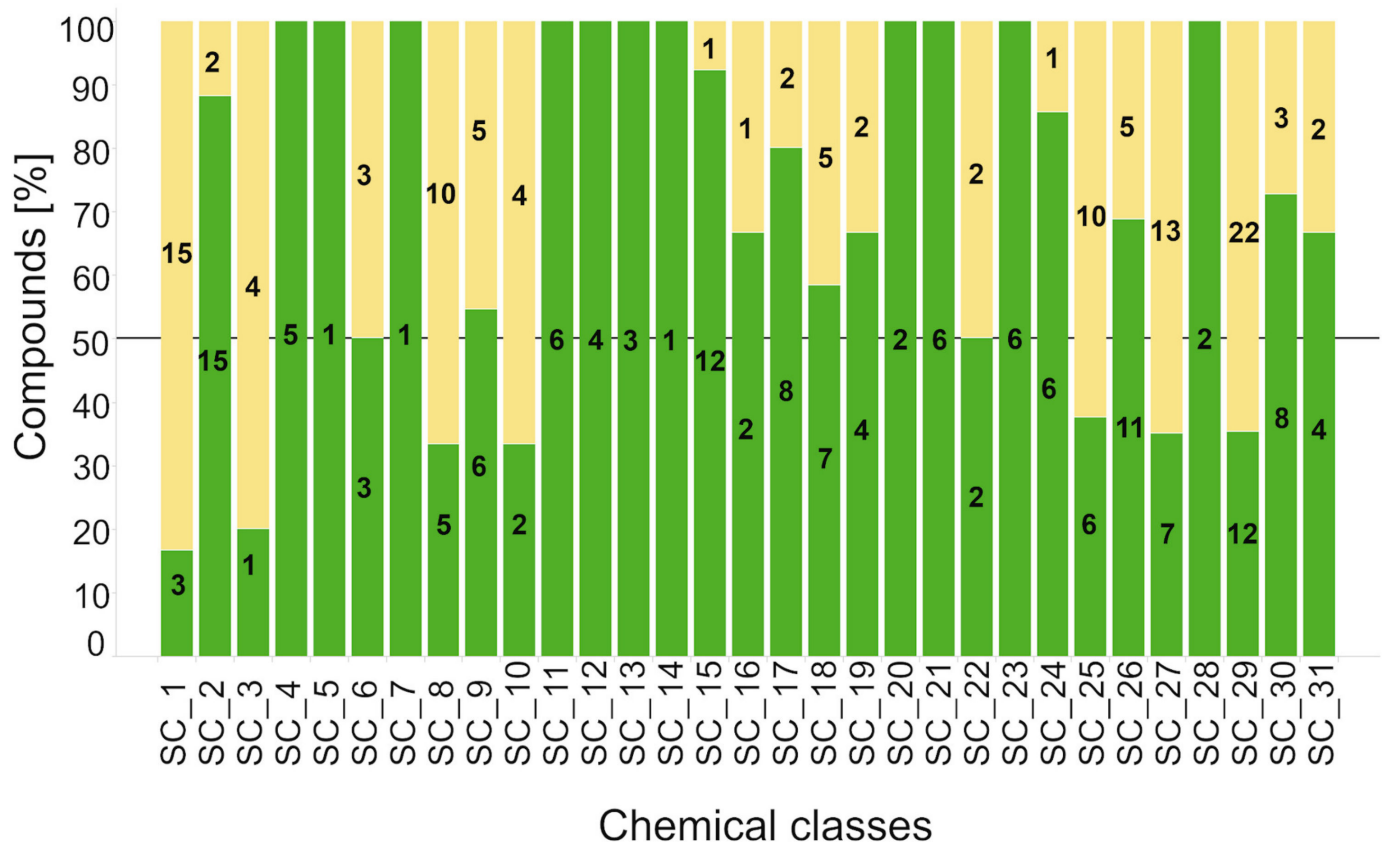
Stacked bar chart showing percentage of true hits (green) and interfering compounds (yellow) within each chemical class (SC_1–SC_31).

For SAR purpose, the 161 true hits were grouped by visual inspection based on common cores, intended as the initial InChyKey scaffolds stripped of the appended groups. This analysis allowed to find 31 different cores, defined as SC_1–SC_31. To possibly identify critical interfering behavior across these 31 scaffolds, the distribution of both true and false positives within each chemotype was analyzed (Figure 6).

**Figure 7:**
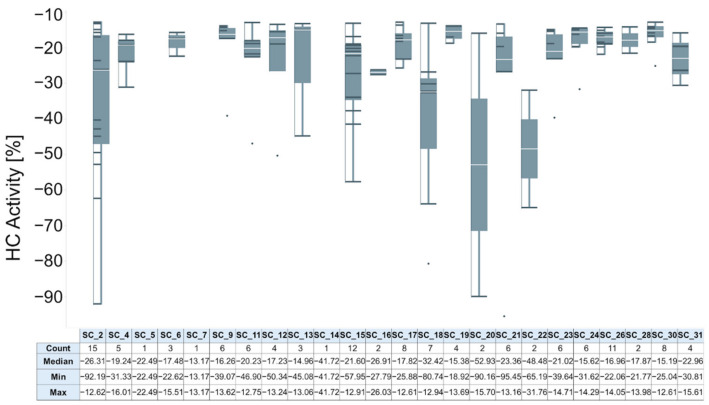
Distribution of activity percent within the scaffolds. In the box plot, a white line within the boxes marks the median value. Dots below the whiskers indicate outliers. For each scaffold, the numbers of hits, median, minimum, and maximum activity percent values are reported in the table.

Interestingly, the interfering behavior was found to be highly associated to some classes, such as SC_1 and SC_3 with a rather low percentage of true hits (below 25%). On the other hand, regardless of the number of actives, SC_4, SC_5, SC_7, SC_11–SC_14, SC_20, SC_21, SC_23, and SC_28 merely encompassed true positives with no interfering molecules. Other classes showed a higher number of true positives compared to interfering compounds. For instance, SC_2 was characterized by 15 true hits versus only 2 false positives, and SC_15 grouped 12 true hits out of 13 compounds. On the basis of such analysis, we reasoned that the number of true positives per class could be used as a criterion for prioritizing promising chemotypes for OXA-48 inhibition. Therefore, seven classes with more than 50% of false positives (SC_1, SC_3, SC_8, SC_10, SC_25, SC_27, and SC_29), including 36 active compounds, were evaluated of low interest, and discarded from the HTS triage. Thus, the pool of true hits turned into 125 molecules, which were then investigated in terms of activity distribution across the corresponding 24 scaffold families. As shown in the box plot of Figure 7, the actives within each class were basically condensed in the top part of the chart, between −30% and the fixed activity cut-off of −12.60%.

In some cases, a higher spread distribution was observed with some representatives showing lower activity percent compared to the majority of the related class members. For instance, SC_2, SC_18, and SC_21 were characterized by active outliers below −80% and median activity between −33 and −23%. Notably, SC_20 and SC_22 showed median activity around −50%. However, the former contained only two molecules with a very different activity profile (−90.16 to −15.70 %), whereas the latter, with equal number of actives, showed a more balanced activity distribution (−65.19 to −31.76 %). We also identified classes with one active compound only, such as SC_5, SC_7, and SC_14. Interestingly, these singleton actives were found to carry specific functional groups not common to other members of the same scaffold, but present on the most potent hits of other classes. These findings suggest that the introduction of punctual chemical features on some chemotypes could potentially improve OXA-48 inhibition.

#### 2.2.3. Hit Expansion

Starting from the 125 confirmed hits, we independently run an InChyKey-based approach and a substructure search on the cores of the 24 classes to identify analogs from the Full Set, thus enriching the numbers of hits and reinforcing the singleton clusters. These two strategies led to the selection of 2003 molecules, which were tested against OXA-48 enzyme at 20 µM in triplicate.

Considering that a precise selection of actives’ analogs was screened at this stage, we applied a more stringent threshold of activity percent (−20%) for hit nomination obtaining 131 additional hits, not interfering in the counter-screening assay.

**Figure 8:**
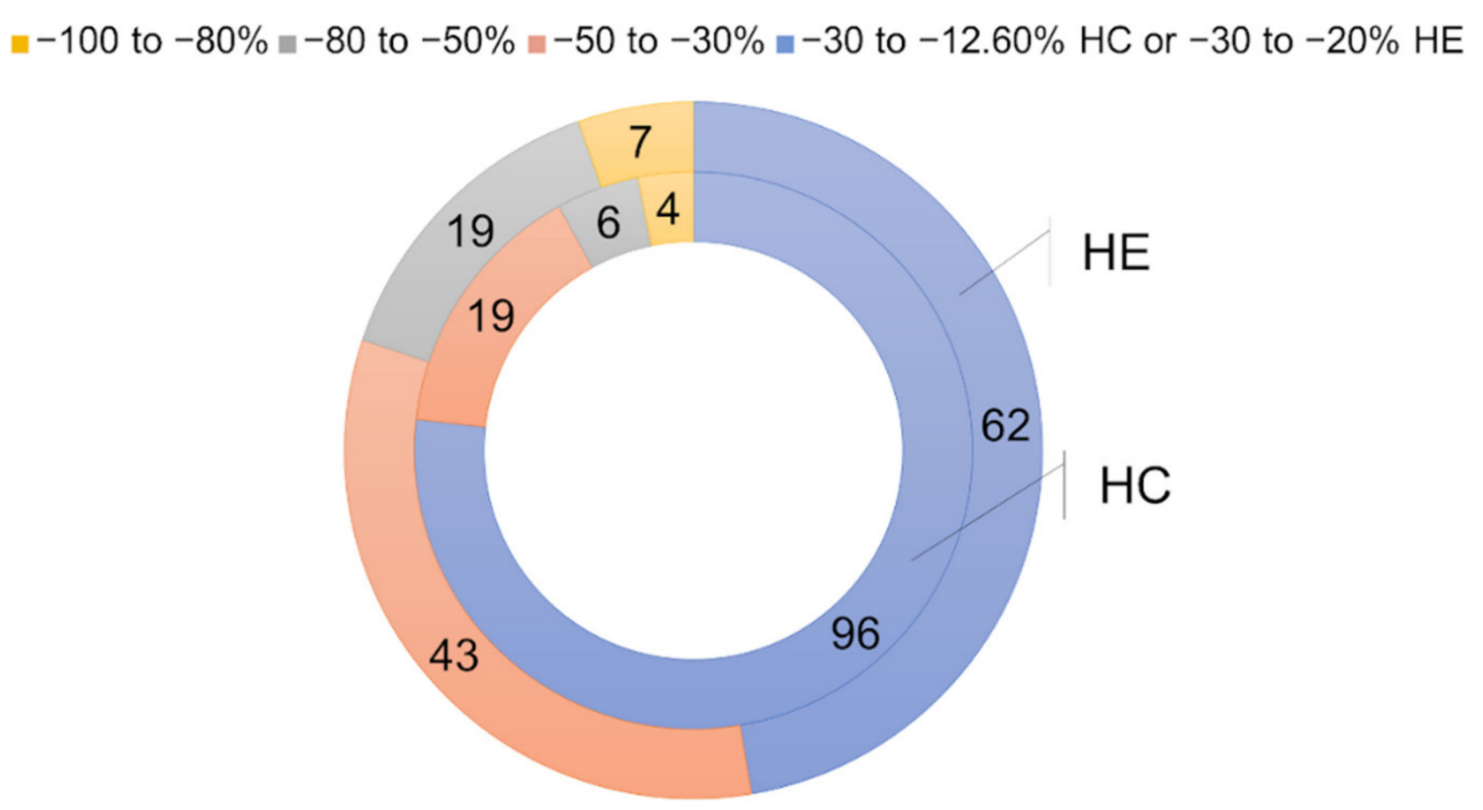
Donut chart showing the number of compounds in i-iv ranges of activity percent: (i) −100 to −80%, (ii) −80 to −50%, (iii) −50 to −30%, (iv) −30 to −12.60% (HC) or −30 to −20% (HE). Each range was considered as half-closed interval of activity percent in which the largest endpoint was included. The innermost and external rings refer to the HC and HE outcomes, respectively.

Interestingly, when comparing the actives derived from the HC and HE stages in terms of activity percent values (Figure 8), we observed that the number of potent compounds (<−30%) was increased, while poorly active molecules were reduced in the latter stage. Overall, this result highlights the benefit of using the Full Set for a fast and focused search of active compounds driven by the Subset outcomes.

**Figure 9:**
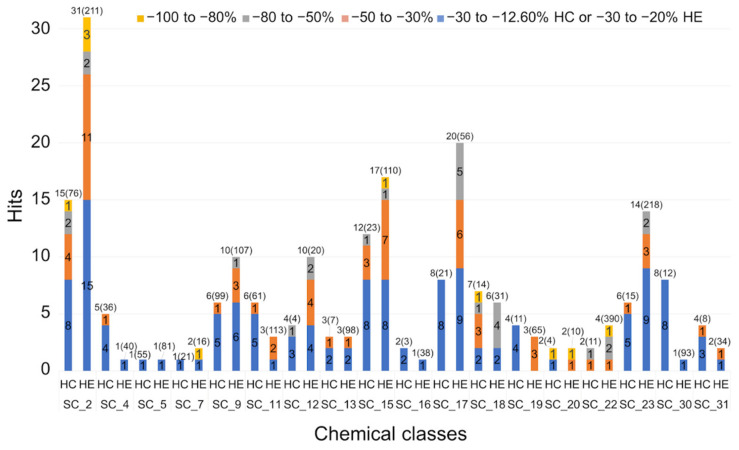
Stacked bar chart showing the hit distribution per scaffold within i–iv ranges of activity percent: (i) −100% to −80%, (ii) −80% to −50%, (iii) −50% to −30%, (iv) −30% to −12.60% (HC) or −30% to −20% (HE), as obtained from the HC and HE stages. Each range was considered as half-closed interval of activity percent in which the largest endpoint was included. On the top of the bars, the total hits and the total tested compounds in brackets are reported. For the sake of simplicity, scaffolds without active representatives in the HE are not reported in the plot.

The activity percent distribution of HC and HE hits per chemical class was also analyzed (Figure 9).

In HE some scaffold families did not confirm activity (SC_6, SC_14, SC_21, SC_24, SC_26, and SC_28), while other ones like SC_2 and SC_15 were conspicuously enriched of actives that populated the most promising range of activity (< −80%) with three and one new hits, respectively. A higher distribution toward more negative activity percent values in HE compared to HC was also observed for SC_9, SC_17, SC_22, and SC_23, resulting suitable chemotypes for OXA-48 inhibition. Similarly, SC_7 was made up of three active representatives in total, one of which recovered in HE and showing excellent activity (< −80%). This derivative turned out one of the most active compounds tested in the screening cascade (see Table 1), thus proving HE as an excellent strategy for hit finding in the HTS triage.

#### 2.2.4. Activity Determination

In total, 256 active molecules (125 from the subset and 131 from the full set) were then assayed in dose–response at eight concentrations from 60 to 0.002 µM with interplate triplicate data points. Overall, 176 compounds showed measurable enzymatic inhibition against OXA-48 (Supplementary Figure S1a), with a final hit rate of 5.6% with respect to the total compounds tested in the HC and HE stages (3130 compounds). Notably, while 88 compounds displayed AC_50_ higher than 60 µM, 13 molecules resulted active in the single digit micromolar range (bar chart in the Supplementary Figure S1a). Interestingly, compounds that did not confirm OXA-48 inhibitory potency in dose–response also showed poor activity in the single point screening, with activity percent higher than −50%.

As for scaffold distribution, the 176 active molecules were found in 20 chemotypes (SC_6, SC_13, SC_14, and SC_28 included inactive compounds only, Supplementary Figure S1b). Overall, the confirmed inhibitors maintained a repartition per chemical class similar to the previous stages. For instance, SC_2, SC_15, SC_17, and SC_23 confirmed to be composed of many active compounds (Table 1).

To proceed with the most promising compounds towards hit optimization, the 20 identified chemical scaffolds were also examined based on the related hit rate with respect to the whole HTS campaign and AC_50_ activity values (Table 1). Interestingly, among the classes with higher hit rate, SC_2 was worthy of investigation. In fact, not only did we identify SC_2 as the most populated group of confirmed hits, but also it contained the largest number of inhibitors with AC_50_ values < 5 µM and no less important the most potent representative of the HTS cascade (AC_50_ = 0.72 µM).

Analyzing the other potent chemotypes, SC_7 and SC_22, with hit rate of 5.4% (2 hits out of 37 tested compounds) and 0.7% (3 hits out of 401 tested compounds) respectively, included the next most active hits with AC_50_ values around 1 µM.

**Figure 10:**
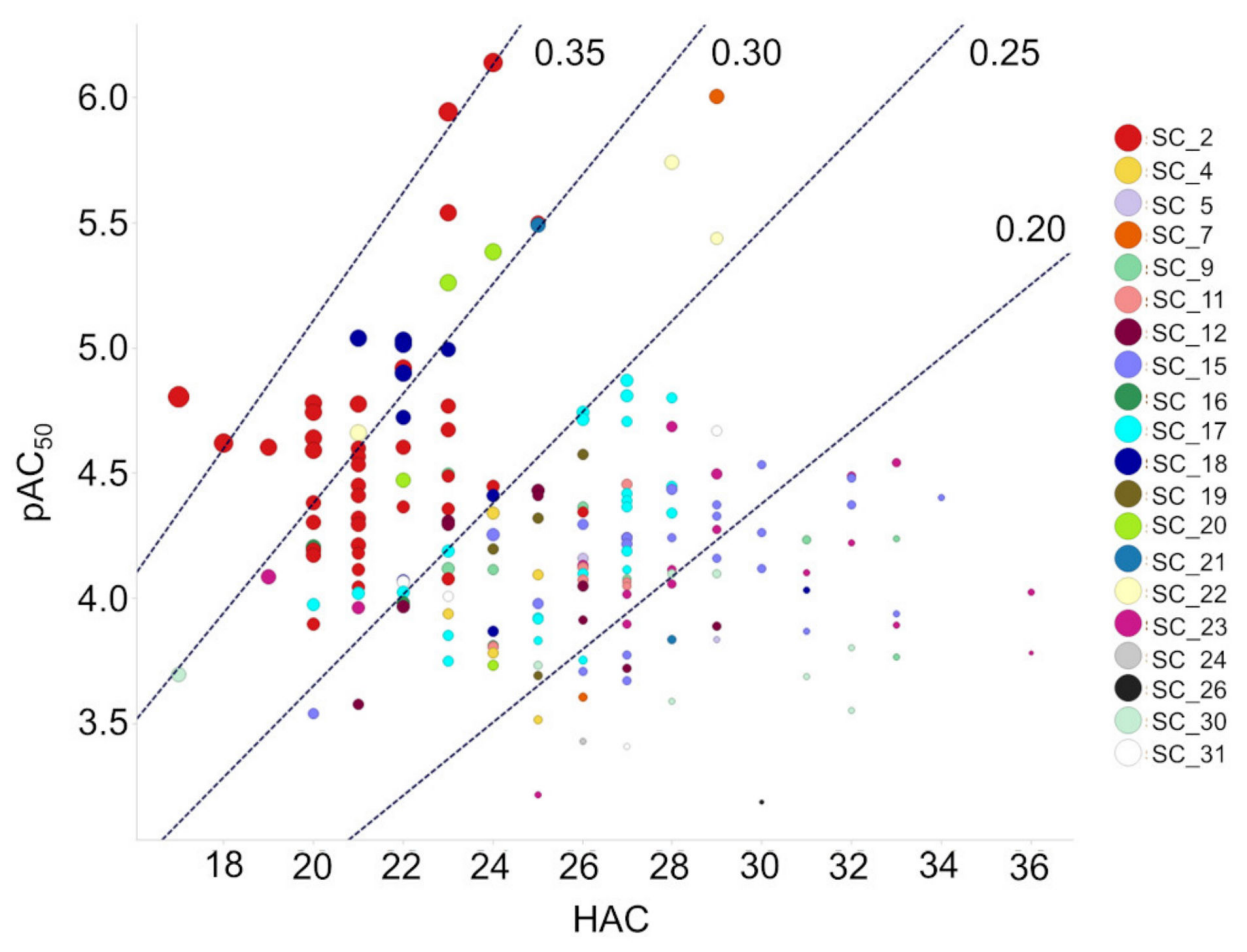
Scatter plot of pAC_50_ versus HAC of the confirmed OXA-48 inhibitors colored by chemical classes. The size of the dots is related to the LE value series of compounds. Diagonal lines represent areas of LE values defined by the equation LE = (1.37/HAC) × pAC_50_.

Finally, due to the common tendency of HTS campaigns to detect hit compounds with inflated physicochemical features, such as high molecular weight and lipophilicity, the 176 actives were analyzed in terms of ligand efficiency (LE) metrics. This widely applied approach enables to assess and prioritize HTS hits with the most favorable combinations of physicochemical and pharmacological properties for the forthcoming optimization phase, ultimately offering more chances of success to the drug discovery program [52]. In Figure 10, pAC_50_ data were plotted as function of the corresponding heavy atom count (HAC), and diagonal lines were reported to depict LE areas. Interestingly, this analysis revealed that SC_2 occupied mainly the region of the plot characterized by the most favorable LE values (LE > 0.25), indicating an efficient ligand-target binding for this chemical series. In contrast, representatives of the other classes were scattered with prevalence towards lower LE values.

Overall, the active chemical scaffolds identified during the HTS campaign were evaluated considering different aspects. Although several classes showed favorable properties, we initially focused on the most active hits belonging to SC_2 and SC_7 families. Beyond showing a reasonable hit rate, good activity on the target and excellent LE values, SC_2 with the common 4-ideneamino-4*H*-1,2,4-triazole core, was known to be active against metallo β-lactamase enzymes [53,54,55,56]. However, this class provided enough confidence in terms of intellectual property (IP) rights and chemical accessibility for further exploration.

SC_7, defined by the pyrazolo[3,4-b]pyridine core, was mainly selected because of the good biological activity against OXA-48 enzyme, promising LE metrics, significant chemical novelty and IP accessibility in the antibacterial therapeutic area, as well as good chemical feasibility. Despite the low hit rate of this scaffold, SAR analysis on the screening collection (see below) suggested space for further investigation around this chemical class.

In the next sections, representatives of the two selected scaffold families are analyzed and discussed in terms of MoA investigation, preliminary SAR evaluation, as well as structural exploration of ligand-target binding supported by crystallographic studies. The most promising hits were evaluated for in vitro efficacy in a minimum inhibitory concentration (MIC) assay in combination with ad hoc selected β-lactam antibiotics and cytotoxicity and solubility assessment.

### 2.3. Mechanism of Action

The HTS triage includes the need for careful follow-up MoA studies around selected hits. To assess the reversibility of OXA-48 inhibition, the MoA of two representatives of SC_2 and SC_7, namely **ID1** and **ID2** (Table 2) with AC_50_ of 1.14 and 0.99 µM respectively (data from activity determination in HTS), was first evaluated by time-dependent inhibition assays. For irreversible inhibitors, slow inhibition kinetics govern the binding process and commonly used efficacy parameters, such as IC_50_, depend on the enzyme-drug incubation period. Conversely, IC_50_ values of reversible inhibitors do not significantly vary according to preincubation time, due to the rapid equilibrium of binding between the enzyme and the inhibitory drug.

**Figure 11:**
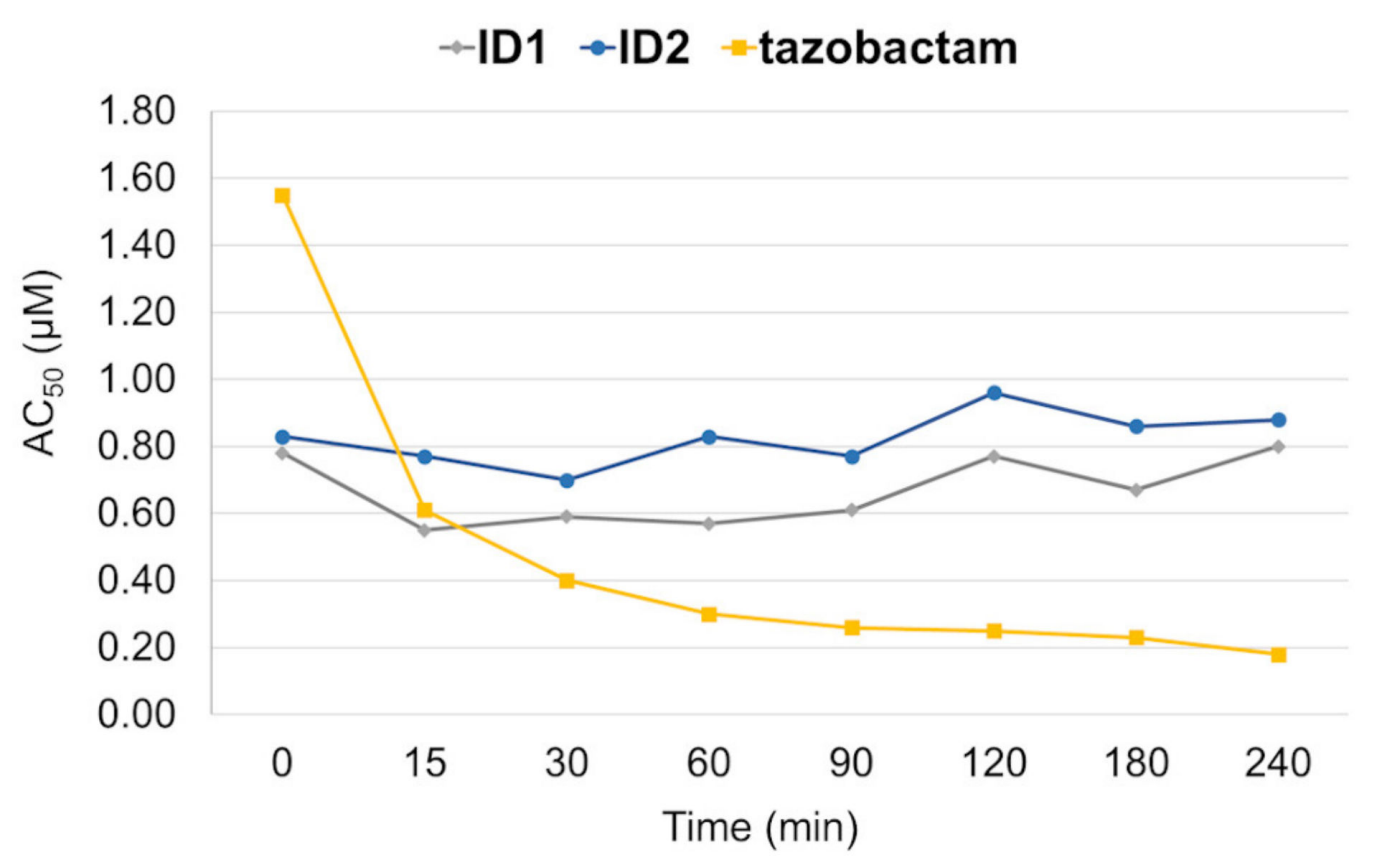
Time dependent inhibition with **ID1**, **ID2**, and **tazobactam**.

On this basis, compounds **ID1** and **ID2** as well as the irreversible reference inhibitor tazobactam were tested in dose–response against OXA-48 lactamase, by pre-incubating the compounds with the enzyme at different time points (0, 15, 30, 60, 90, 120, 180, 240 min) before starting the reaction upon addition of the nitrocefin substrate. Interestingly, AC_50_ values of compounds **ID1** and **ID2** were independent on the preincubation time with OXA-48, suggesting a reversible inhibition of the enzyme (grey and blue lines respectively in Figure 11). On the other hand, preincubation of tazobactam with OXA-48 caused an inversely proportional time-dependent inhibition of the carbapenemase activity (yellow line in Figure 11), confirming that a tight enzyme-ligand adduct was formed in a time-dependent fashion. Indeed, the AC_50_ value of tazobactam reached a plateau after 90 min of preincubation and was almost six-fold lower than the corresponding AC_50_ value obtained without preincubation.

To confirm the reversibility of OXA-48 inhibition by compounds **ID1** and **ID2**, dilution-after-preincubation tests were also performed. Specifically, the experiments were conducted by preincubating (0 and 60 min) five-fold concentrated test compounds and OXA-48 enzyme, next diluting (1:5) the assay well to the standard reaction conditions and adding the nitrocefin substrate. In parallel, a control experiment with compounds **ID1** and **ID2** tested under the standard protocol was also run. Based on the assay design, reversible inhibitors are expected to regain enzyme activity after dilution of the enzyme-inhibitor (EI) complex and show no significant difference between AC_50_ values from samples preincubated under high-concentration and standard conditions. Conversely, irreversible inhibitors are envisaged not to recover any enzyme activity following dilution of the tight EI complex, which will translate in a substantial decrease in OXA-48 AC_50_ of high-concentration preincubated samples. As shown in Table 2, the dilution-after-preincubation experiment highlighted that the AC_50_ values of compounds **ID1** and **ID2** were not modulated by the preincubation with OXA-48 at high concentration compared to the standard conditions, thus supporting a reversible MoA. On the contrary, the effect of dilution on enzyme inhibition was observed for the irreversible compound tazobactam (Table 2). The AC_50_ value obtained using concentrated reagent solutions at the preincubation step (90 min as from the time-dependent inhibition study) was almost three-fold lower than the AC_50_ value obtained under standard assay protocol.

**Figure 12:**
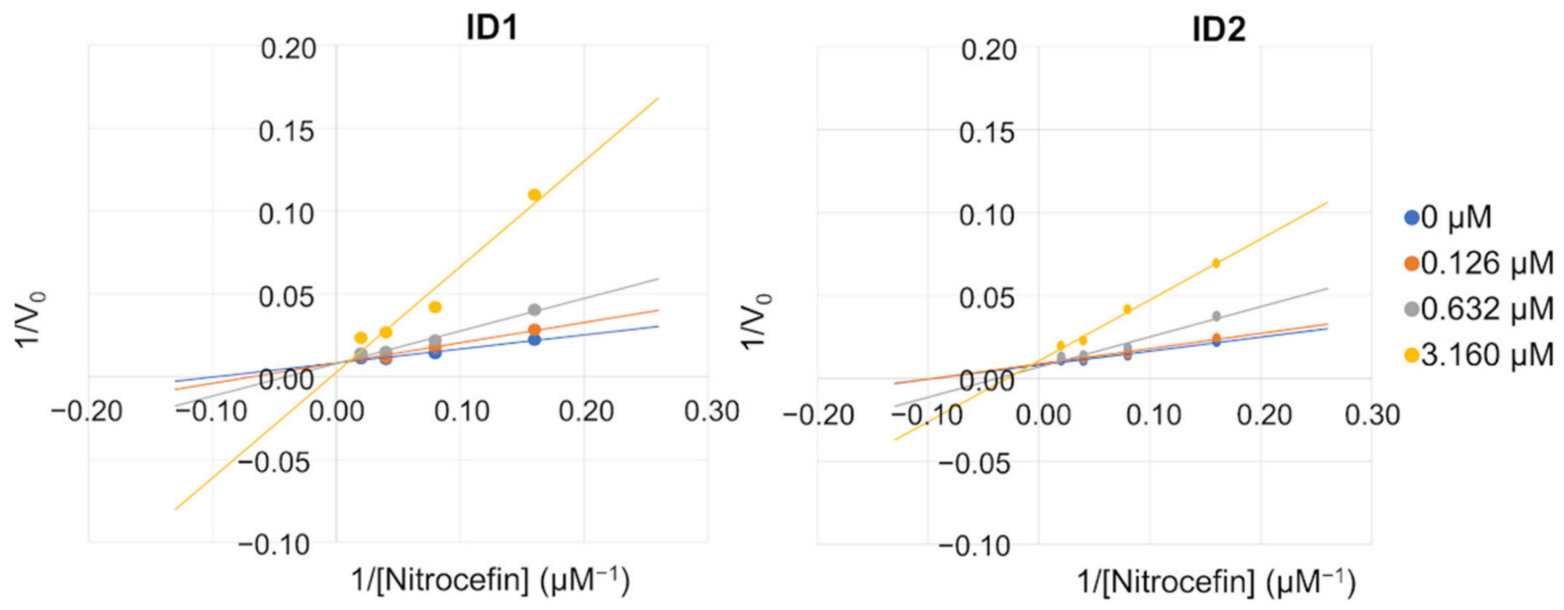
Lineweaver–Burk plots showing competitive inhibition of OXA-48 with respect to nitrocefin using **ID1** and **ID2**. Inhibitor concentrations were 0 (blue), 0.126 (orange), 0.632 (grey), and 3.160 µM (yellow).

To further elucidate the type of reversible inhibition of compounds **ID1** and **ID2**, double substrate (nitrocefin) and inhibitor titrations were run. Figure 12 shows that the experimental results fit well to a competitive inhibition model in the Lineweaver–Burk plots, with a K_i_ of 0.32 and 0.33 and an α value 5.50 and 5.44 for compounds **ID1** and **ID2** respectively, indicating that the inhibitors preferentially bind to the free enzyme.

In both cases the nitrocefin K_m_ value was directly proportional to the inhibitor concentration, while the V_max_ value was independent (Table 3), suggesting a substrate competitive OXA-48 inhibition.

### 2.4. SC_2: SAR from HTS

A set of 287 4-ideneamino-4*H*-1,2,4-triazole derivatives, including compounds with multiple substitutions around the central core, as well as constraint analogs, was screened in the HTS campaign (see Supplementary Materials). Overall, 38 compounds showed measurable OXA-48 AC_50_ values (Supplementary Table S1) and four derivatives (**ID1**, **ID3**, **ID4**, and **ID5**) resulted active in the sub- and single-digit micromolar range.

Herein, the SAR around the most active 4-[(3-sulfanyl-4*H*-1,2,4-triazol-4-yl)imino]methyl benzoic acids **ID1** and **ID3**-**ID5** is discussed, considering both positive and negative results from the whole screening collection (Table 4).

Compound **ID3** resulted the most potent derivative of the series, with OXA−48 activity percent at 20 μM = −94.1% and OXA−48 AC_50_ of 0.72 μM. Interestingly, despite the competitive MoA anticipated for this chemical class, compound **ID3** was as potent as the covalent reference compound tazobactam (AC_50_ = 0.84μM). Replacement of the 4-chlorophenyl ring of **ID3** with different hydrophobic aryl or cycloalkyl groups, such as phenyl (**ID1**), 2,4-dichlorophenyl (**ID4**), and cyclohexyl (**ID5**) moieties retained activity in the same range (AC_50_ = 1.14–3.17 μM) of the parent compound. Conversely, removal of the 3-substituent on the triazolo ring (**ID6**) led to more than 20-fold loss in potency, suggesting that a bulky moiety at this position is beneficial for activity. Additionally, it seems that differently substituted phenyl rings as well as heterocycles—such as *p*-pyridine, *o*-furan, and pyrazole—might be tolerated at this site (Supplementary Table S1).

Several close analogs of hits **ID1** and **ID3**-**ID5**, sharing the same chemical structure but the *p*-benzoic acid moiety, were also found in the screening collection. These compounds allowed to specifically evaluate the role of this acidic functional group in OXA-48 binding. Indeed, all derivatives, such as **ID7-29** in Table 4, showed reduced activity compared to the parent compounds.

Specifically, when moving the carboxylic acid from the *p*- to the *o*-position of the phenyl ring of **ID5**, a poor OXA−48 activity percent at 20 μM = −1.24% (**ID7**) was observed in comparison to the corresponding hit (−88.29%). Additionally, replacement of the *p*-COOH with functional groups (namely -H, -COOCH_3_, -CN, -F, -Cl, -Br, -NO_2_, -CH_3_, -SCH_3_, -OH) having different electronic (inductive and mesomeric), lipophilic, and steric properties to suitably cover the Craig plot [57], was generally detrimental for activity. Indeed, compounds **ID9-19**, showed activity percent at 20 μM ranging from −58.40% to 0.02. Also, *o*- and *m*-substituted phenyl moieties did not result in any improvement in activity (see Supplementary Materials). Only the *p*-COOCH_3_ (**ID9**) and *p*-CN (**ID11**) derivatives showed measurable AC_50_ values of 35.60 and 12.00 μM, respectively, suggesting that substituents having -σ and + π effects, similarly to the COOH group, might be better tolerated.

Finally, replacement of the *p*-benzoic acid moiety with different heterocycles, like furan (**ID20–22**), pyridine (**ID24–26**), and ethenylfuran (**ID27–29**) was also unfavorable, leading to activity percent at 20 μM higher than −15.4%. Several inactive derivatives carrying methyl-substituted heterocycles were also contained in the whole dataset (see Supplementary Materials). Only the thiophen derivative **ID23** showed OXA-48 activity percent at 20 μM = −29.46% and OXA−48 AC_50_ of 24.80 μM.

Overall, the SARs observed along the screening collection revealed the paramount role of *p*-benzoic acid moiety for activity. Indeed, all compounds in the dataset containing this functional group showed activity percent at 20 μM higher than −61.30% and AC_50_ values ranging from 0.72 to 15.70 μM. Importantly, the structural role of this moiety was also confirmed by X-ray studies.

A crystal structure study was carried out to elucidate the binding mode of **ID3** as representative of SC_2 in complex with OXA-48 (see Experimental Section for details). We obtained a 1.65 Å resolution data set including two monomers in the asymmetric unit (PDB code 7AW5). The electron density of the ligand showed an unambiguous binding mode and equivalent interaction pattern in the two monomers.

**Figure 13:**
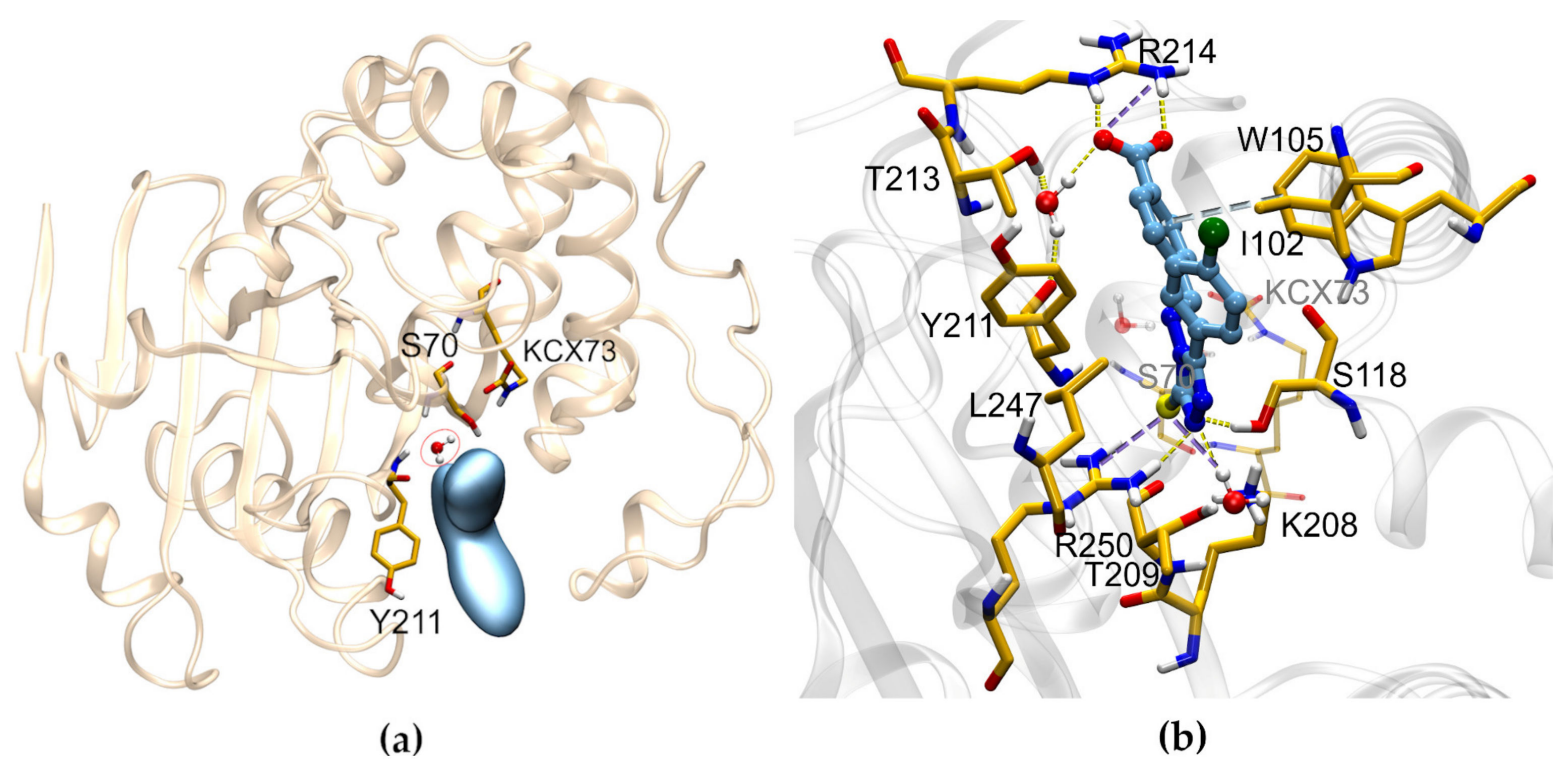
X-ray cocrystal structure of **ID3** in complex with OXA-48 enzyme (PDB code: 7AW5), reported as cyan and yellow representations, respectively. (**a**) OXA-48 catalytic pocket occupied by ID3 (cyan surface). Carboxylated Lys73 (KCX73), catalytic Ser70 and Tyr211 (oxyanion hole), and the catalytic water molecule (red circle) are reported. (**b**) **ID3** and the most relevant residues of the binding pocket. Hydrogen bonds, electrostatic and π–π interactions are represented as yellow, purple, and grey dashed lines. For the sake of clarity, some portions of the protein have been omitted.

The inhibitor occupied the entrance of the catalytic pocket (Figure 13a) where Lys73 was observed in the carboxylated form, an important functionalized residue for the β-lactam inactivation mechanism [58]. Typically, the so called ‘oxyanion hole’ delimited by the catalytic Ser70 and Tyr211 residues is filled with a catalytic conserved water molecule involved in the deacetylation process [59] and accommodates β-lactam group liable to the hydrolysis. In the solved crystal structure, we observed such solvation network (Figure 13a).

An ionic interaction between the thiolate of the ligand and the basic network identified by Arg250 and Lys208 was observed (Figure 13b and Supplementary Figure S2a). Interestingly, the positively charged cavity consisting of Arg250 and Lys208, and Thr209 at the entrance of the binding pocket recurs to engage the carboxylate group of carbapenems like IMP, meropenem ertapenem and doripenem [60]. Similarly, the sulfate group of avibactam is found to contact the guanidinium group of Arg250 [61]. This common feature validated the role of **ID3′**s thiolate group and brought out its significant contribution to the affinity for OXA-48 enzyme in the SC_2 series. Another interaction of such negatively charged group consisted of a hydrogen bond with Ser118, whereas the triazole nitrogen engaged Thr209 via a water molecule and Arg250. Moving to the R1 group (Supplementary Figure S2b), hydrogen bonds and another ionic interaction were established between the benzoic acid and Arg214. The carboxylic group was also involved in water mediated contact with Thr213 and Tyr211, and a π–π stacking was observed between Trp105 and the phenyl ring of the benzoic acid. Lastly, the *p*-chlorophenyl moiety of the ligand was surrounded by the hydrophobic Leu247 and Ile102. Figure 13b and Supplementary Figure S2 highlight the discussed interaction pattern of **ID3** in complex with OXA-48.

Overall, this crystal structure showed the two negatively charged groups of **ID3** properly oriented to reach Arg214 and Arg250 via electrostatic interactions, in addition to other contacts with the neighboring residues. Notably, the observed binding pose supports that **ID3** binds in the active site of OXA-48 in a reversible manner, in agreement with the experimental MoA discussed for the close analog **ID1**.

### 2.5. SC_7: SAR from HTS

A small dataset of 37 pyrazolo[3,4-b]pyridine derivatives was screened in the HTS campaign (Supplementary Table S2).

The tested set allowed to explore several decorations at different positions of the central scaffold, which generally resulted unsuccessful in modulating OXA-48 functionality. Compound **ID2** came out as the only active molecule of the series (singleton active) with an activity percent at 20 μM = −93.92% and AC_50_ of 0.99 μM (Table 5)

Interestingly, SAR analysis showed that hit **ID2** was the only derivative carrying a *p*-benzoic acid at position 6 of the pyrazolo[3,4-b]pyridine core, suggesting the key role of this functional group for the inhibitory activity. Indeed, the close analogs **ID30** and **ID31**, sharing the same chemical structure of **ID2** but the 6- *p*-benzoic acid moiety, resulted inactive (Table 5). These findings corroborated the idea that carboxylic acid functions are relevant for OXA-48 binding, as observed for other chemical classes tackling this enzyme. In this respect, we reasoned that the identified key *p*-benzoic acid was underrepresented in the tested library, thus possibly limiting the relevance of the SAR generated around SC_7 toward the target of interest. Therefore, we envisaged that the observed negative SAR of the near neighbors could have not covered all appropriate changes in the hit compound and further understanding of what was offered from singleton **ID2** was worth the effort for additional investigation.

On this basis, a small set of compound **ID2′**s analogs (**1**–**14** in Table 5) was designed and synthesized to better elucidate the role and evaluate possible substituents of carboxylic acid groups at position 4 and 6 of the pyrazolo[3,4-b]pyridine core [62].

### 2.6. Chemistry

**Scheme 1:**
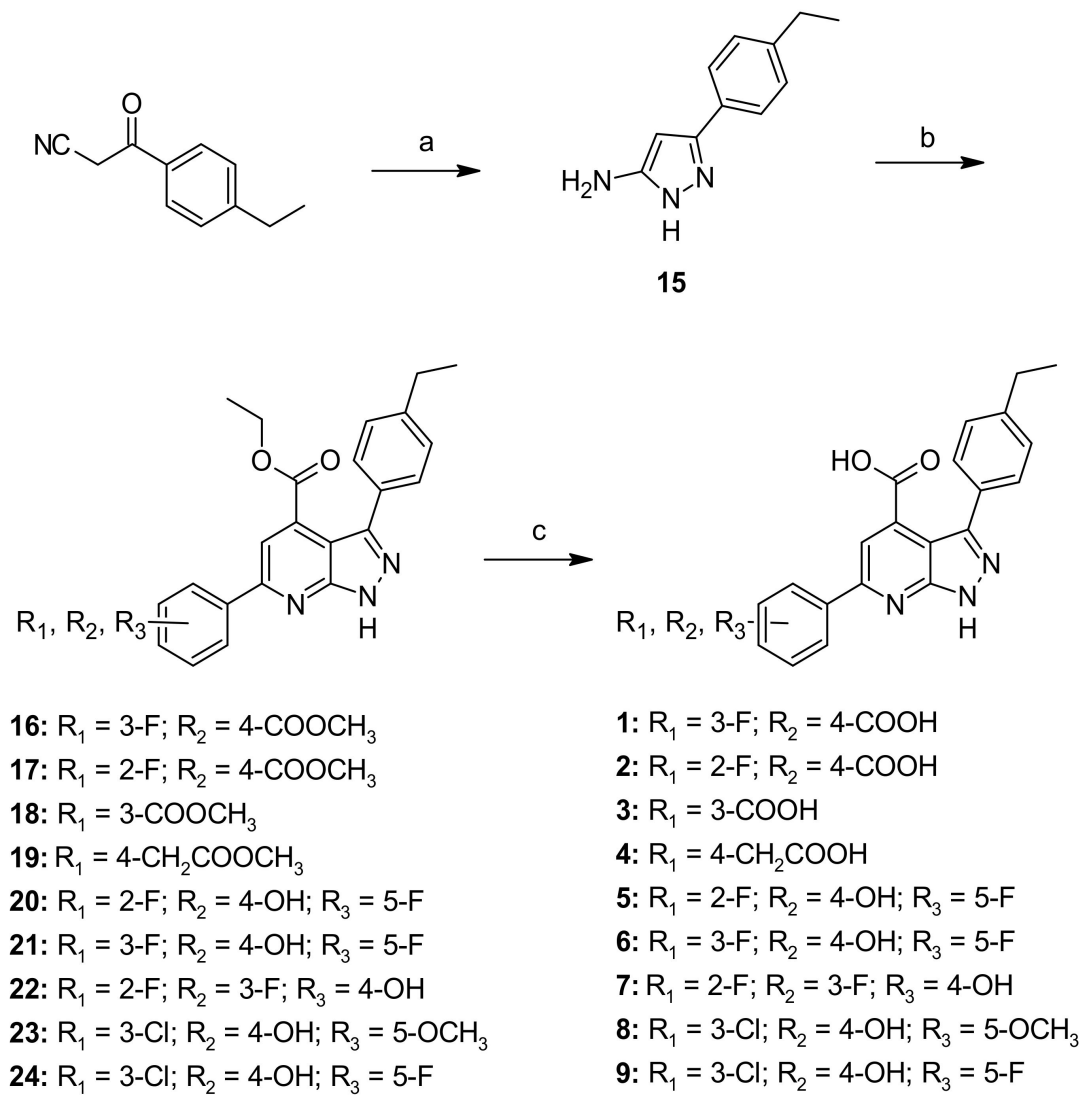
(a) H_4_, MeSO_3_H, EtOH, 80 °C, 100 min, 72%; (b) ethyl pyruvate, aryl aldehyde, HCl, EtOH, MW 150 °C, 10–30 min, 8–29%; (c) LiOH, MeOH-THF-water (1:1:1), 40–45 °C, 20–72 h, 27–94%.

A survey of 6-benzoic acid replacements on the 1*H*-pyrazolo[3,4-b]pyridine-4-carboxylic acid core was undertaken (Scheme 1). For this purpose, the 3-(4-ethylphenyl)-1*H*-pyrazol-5-amine intermediate **15**, prepared by treatment of 3-(4-ethylphenyl)-3-oxopropanenitrile with hydrazine, was coupled to ethyl pyruvate and the desired aryl aldehyde in a microwave-assisted multi-component cyclization reaction, leading to compounds **16**–**24**. Subsequent ester hydrolysis with LiOH provided the target compounds **1**–**9**.

**Scheme 2:**
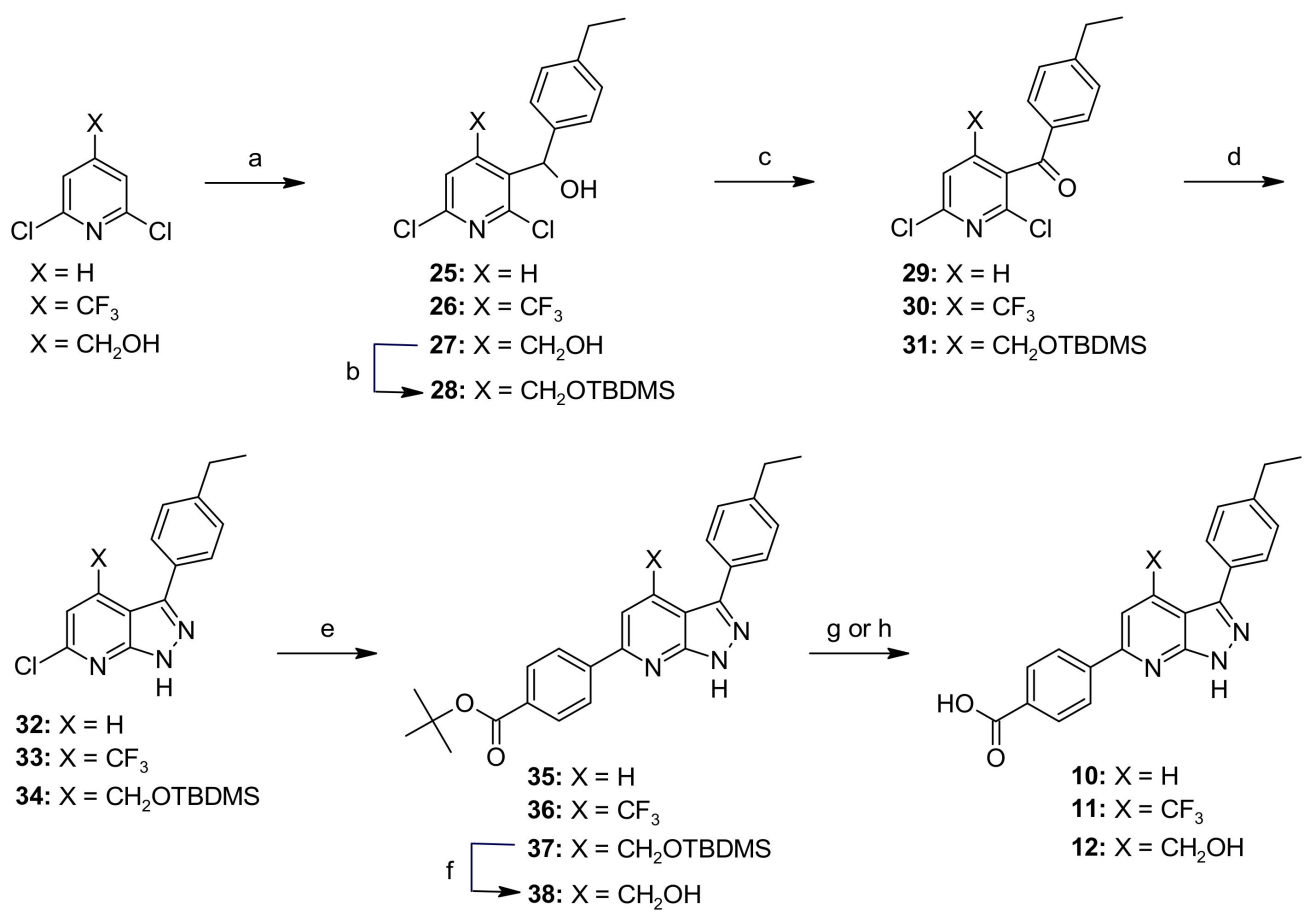
(a) 4-ethylbenzaldehyde, LDA, THF, −78 °C, 15 min, 23–95%; (b) TBDMS-Cl, DIMAP, imidazole, DCM, rt, 16 h, 91%; (c) Dess-Martin periodinane, DCM, rt, 15–30 min, 80–99%; (d) N_2_H_4_, DIPEA, EtOH-THF (4:1), 80 °C, 1.5–16 h, 43–83%; (e) (4-(*tert*-butoxycarbonyl)phenyl)boronic acid, K_2_CO_3_, Pd(dppf)Cl_2_.CH_2_Cl_2_, dioxane-water (4:1), MW 120–140 °C, 1–3h, 49–54%; (f) TBAF, THF, rt, 16 h, 52%; (g) TFA, DCM, rt, 16 h, 34–43% (for **10** and **11**); (h) 4M HCl in dioxane, rt, 40 h, 65% (for **12**).

**Scheme 3:**
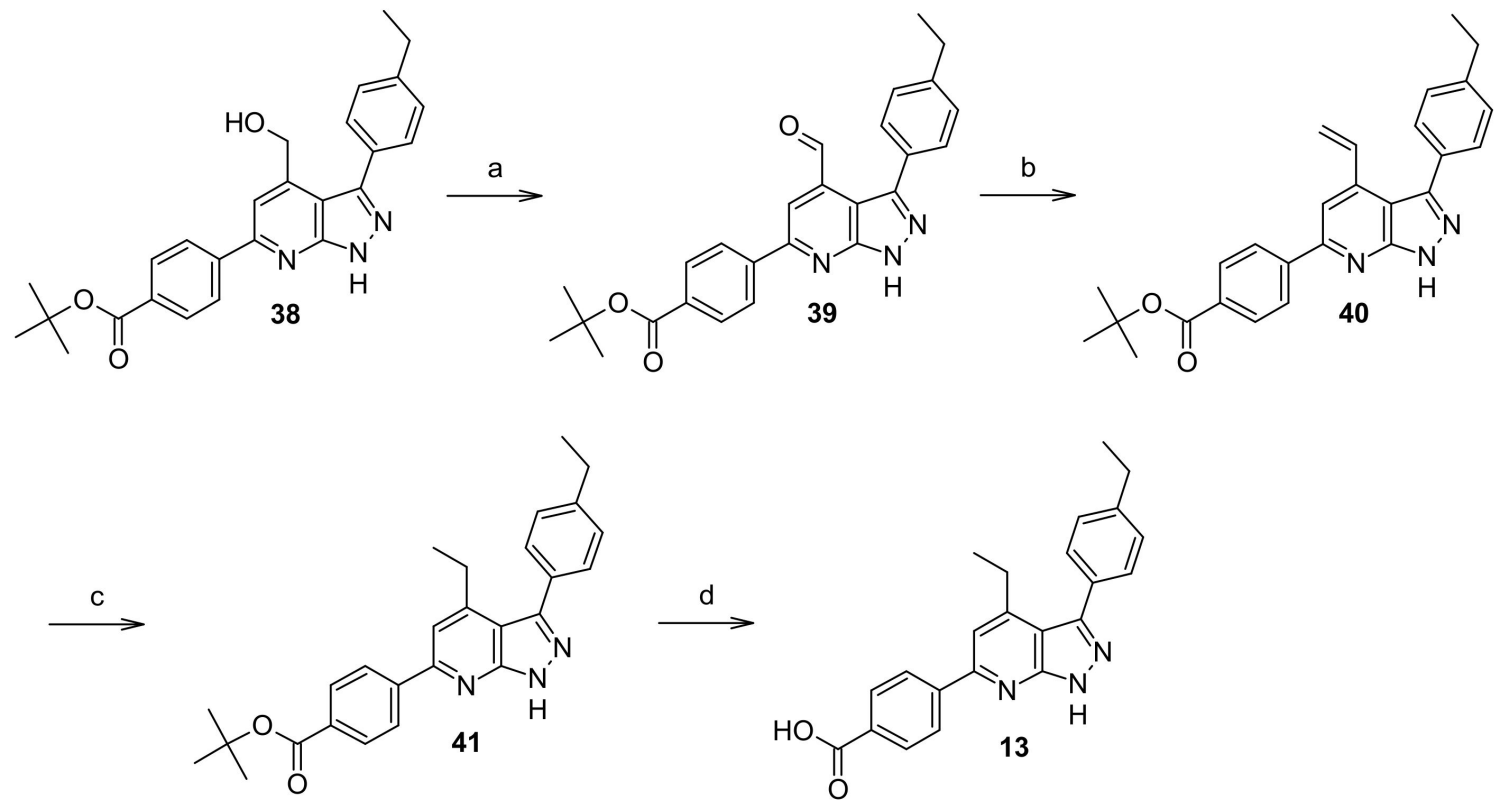
(a) MnO2, DCM, 95 °C, 16 h, 74%; (b) MePPh_3_Br, KOtBu, Et_2_O, rt, 16 h, 78%; (c) H_2_, Pd/C, AcOH, EtOH, 5 bar, rt, 5 h, 91%; (d) 4M HCl in dioxane, rt, 40 h, 31%.

**Scheme 4:**
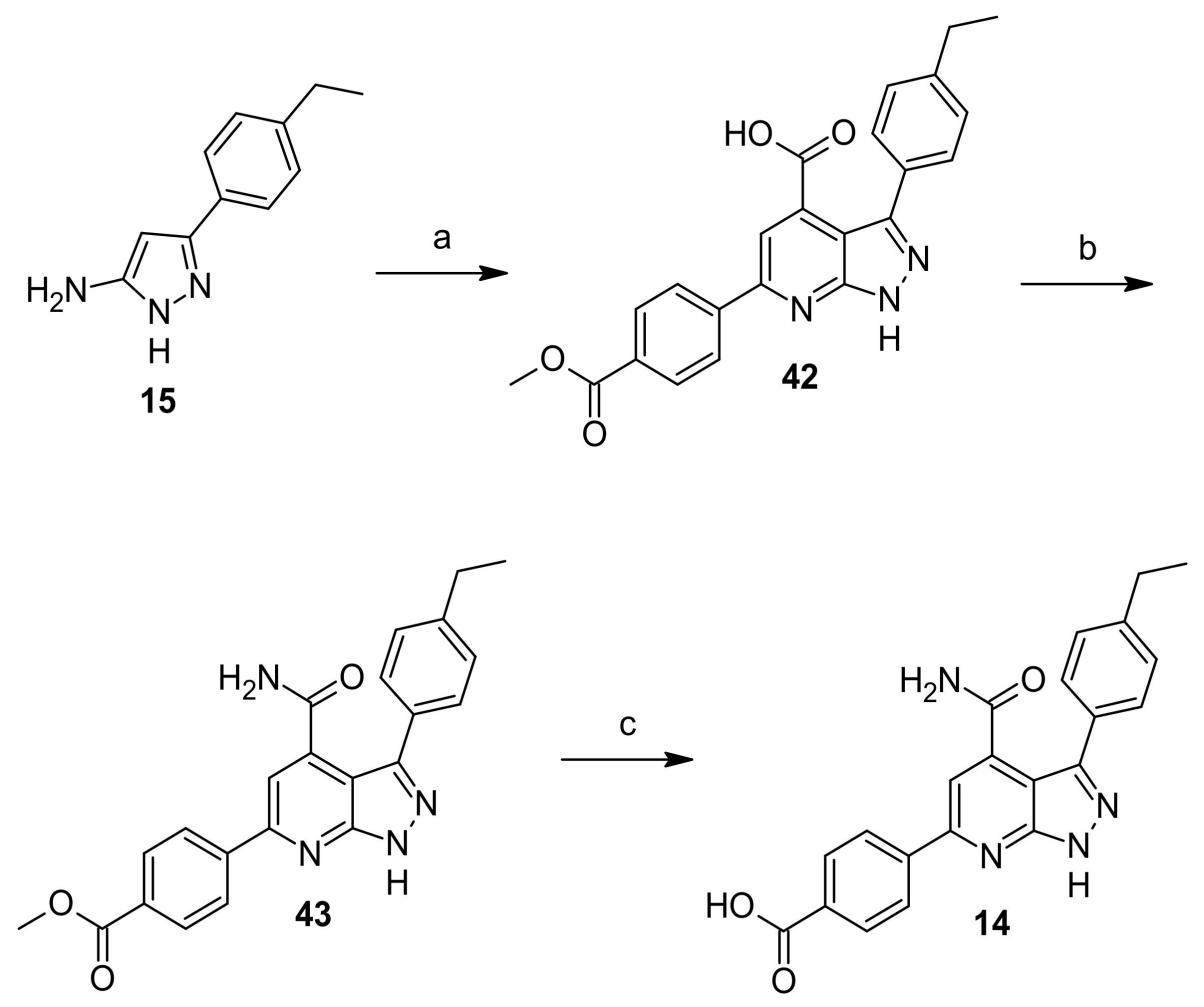
(a) 2oxopropanoic acid, methyl-4-formylbenzoate, HCl, EtOH, MW 150 °C, 15 min, 18%; (b) HATU, NH_4_Cl, DIPEA, DMF, rt, 64 h, 74%; (c) LiOH, MeOH-THF-water (1:1:1), rt, 4 h, 23%.

In parallel to this approach, compounds **10**–**14** were prepared to explore alternative substituents to the carboxylic acid group at 4-position of the 1*H*-pyrazolo[3,4-b]pyridine core (Scheme 2, Scheme 3 and Scheme 4). Derivatives **10**–**12** were synthesized by treating the desired 4-substitued 2,6-dichloropyridine with LDA, followed by nucleophilic addition of 4-ethylbenzaldehyde to provide alcohols **25**–**27**. The di-hydroxy intermediate **27** was selectively protected at the primary alcohol functionality using *tert*-butyldimethylsilyl chloride to afford derivative **28**. Upon oxidation of alcohols **25**, **26**, and **28** with Dess-Martin periodinane to the corresponding diaryl-ketones **29**–**31**, the pyrazolo[3,4-b]pyridine core was formed via reaction with hydrazine in EtOH and THF. With reactants **32**–**34** in place, Suzuki reactions were performed in a microwave reactor in the presence of (4-(*tert*-butoxycarbonyl)phenyl)boronic acid and Pd(dppf)Cl_2_.CH_2_Cl_2_ as a catalyst, affording intermediates **35**–**37**. Deprotection of the TBDMSO group of **37** under standard conditions, followed by hydrolysis of *tert*-butyl esters **35**, **36**, and **38** with TFA in DCM or HCl in dioxane led to compounds **10**–**12** (Scheme 2).

The 4-ethyl-1*H*-pyrazolo[3,4-b]pyridine analog **13** was prepared starting from intermediate **38** as depicted in Scheme 3. Upon MnO_2_-catalysed oxidation of alcohol **38** to aldehyde **39**, the ethenyl intermediate **40** was prepared via Wittig reaction of **39** with methyltriphenylphosphonium bromide. Subsequent hydrogenation of the double bond followed by acid removal of the *tert*-butoxy protecting group of **41** provided the target compound **13**.

Amide **14** was prepared according to the three-step synthesis showed in Scheme 4. The 3-(4-ethylphenyl)-1*H*-pyrazol-5-amine **15** was coupled to 2-oxopropanoic acid and methyl-4-formylbenzoate in a multi-component reaction to afford 1*H*-pyrazolo[3,4-b]pyridine-4-carboxylic acid intermediate **42**. Coupling of the carboxylic acid with ammonium chloride provided amide **43** and subsequent based-catalyzed methtyl ester hydrolysis led the compound of interest.

### 2.7. SC_7: Explorative SAR

Starting from hit compound **ID2**, the effect of introducing electron withdrawing groups like fluorine at position 2 and 3 of the benzoic acid moiety was evaluated. Both modifications were well tolerated, as shown by compounds **1** and **2** that retained the same activity of the parent compound.

The carboxylic acid group was also moved from position 4 to position 3 of the 6-phenyl ring (**3**) resulting in 17-fold loss in activity, whereas substitution of the benzoic with the phenethyl acid provided derivative **4**, which was active in the single digit micromolar range (AC_50_ = 4.78 μM).

Replacement of the benzoic acid group of **ID2** with di-substituted phenols (**5**–**9**) was also explored. This modification was meant to investigate the effect of functional groups such as the hydroxyl moiety, which should mimic the main pharmacophoric features of carboxylic acids and serve as both hydrogen-bond donor (HBD) and hydrogen-bond acceptor (HBA) as well as negative ionizable group. Overall, phenols **5**–**9** resulted active in the double digit micromolar range (AC_50_ = 13.69–63.77 μM) and were from 14- to 64-fold less active than the parent compound. The observed drop in activity might be ascribed to several reasons: i) reduced acidity of phenols (calculated pKa = 6.72–7.59) with respect to **ID2′**s benzoic acid (calculated pKa = 3.62), resulting in diminished negative ionization potential; ii) removal of the oxo-group of the carboxyl moiety, thus losing a HBA functional group; iii) different length and three-dimensional orientation of the -OH and -COOH functional groups. Along the phenol set, 2,5-di-F-phenol **5** (AC_50_ = 16.21 μM) was almost as active as 2,6-di-F-phenol **6** (AC_50_ = 24.38 μM) and 3-fold more potent than the corresponding 2,3-di-F-phenol **7** (AC_50_ = 51.21 μM). 2-Cl-6-OCH_3_-phenol **8** (AC_50_ = 13.69 μM) showed enzymatic potency comparable to **5** and was 4.6-fold more active than 2-Cl-6-F-phenol **9** (AC_50_ = 63.77 μM). These findings highlight that different substituents on the phenol ring can also impact on OXA−48 enzymatic activity, possibly due to steric hindrance, electronic or solvation effects that might ultimately influence the entropic and enthalpic contributions to the binding affinity.

Considering that acidic functionalities resulted relevant for the OXA-48 interaction, we also investigated the effect of the carboxylic acid at position 4 of the pyrazolo[3,4-b]pyridine core on the carbapenemase activity. Interestingly, replacement of this carboxyl moiety with H (**10**), -CF_3_ (**11**), and -CH_2_CH_3_ (**13**) group was detrimental for the enzymatic potency. Also, substitution of the 4-COOH group of **ID2** with -CH_2_OH **(12**) or -CONH_2_ (**14**) resulted in complete loss of activity, suggesting that this acidic feature also dramatically contributes to the inhibitory activity.

**Figure 14:**
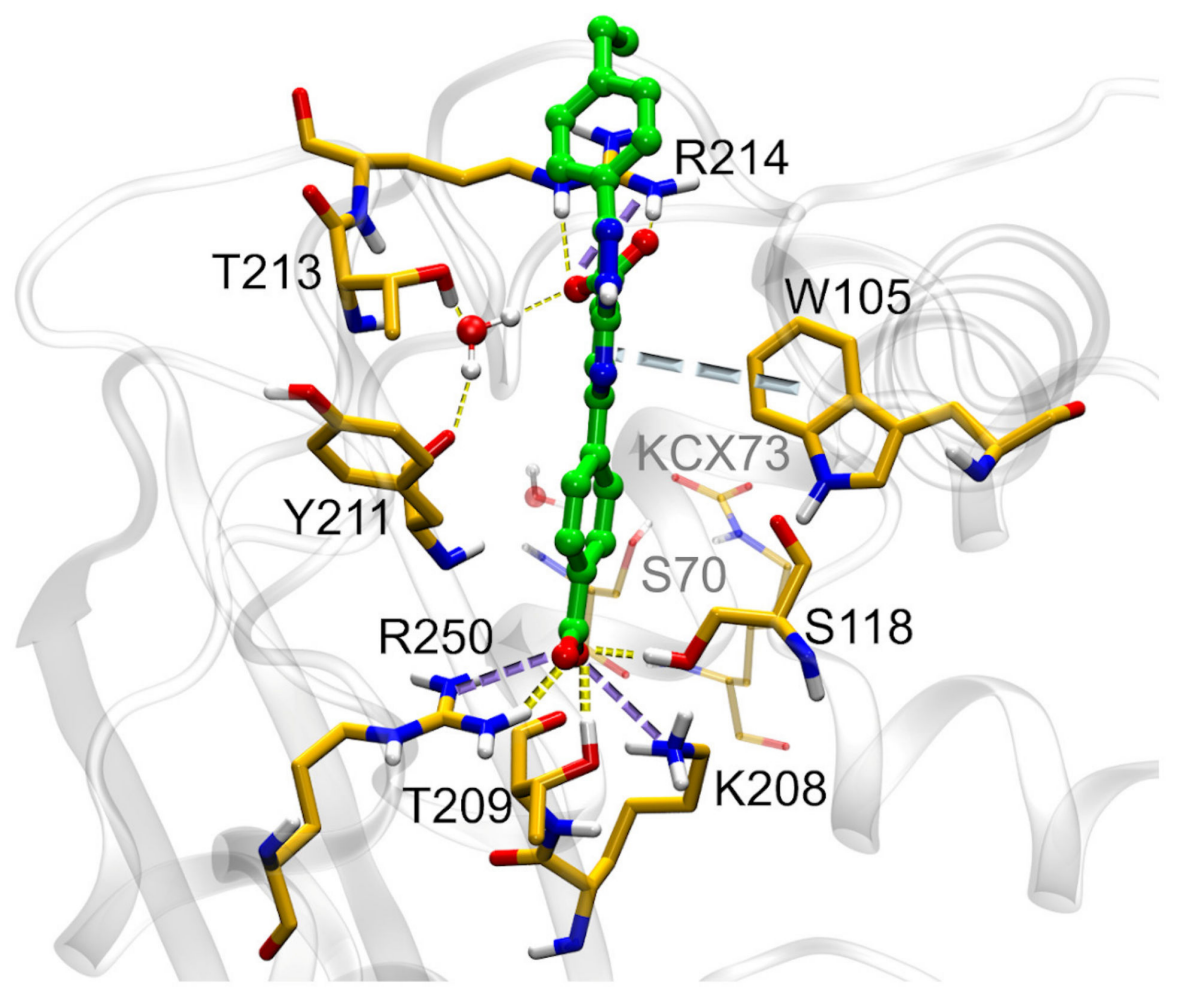
X-ray cocrystal structure of **ID2** in complex with OXA-48 enzyme (PDB code 7AUX). The ligand and the most relevant residues of the binding pocket are reported in green and yellow, respectively. Hydrogen bonds, electrostatic and π–π interactions are represented as yellow, purple, and grey dashed lines. For the sake of clarity, some portions of the protein have been omitted.

The observed SARs were further corroborated and rationalized by the high-resolution (2.05 Å) X-ray crystal structure of OXA−48 enzyme in complex with **ID2** (PDB code: 7AUX), which was solved to elucidate the binding mode of pyrazolo[3,4-b]pyridine derivatives (Figure 14).

The asymmetric unit consists of two OXA−48 molecules, consistent with each other (see Experimental Section). The overall density was unambiguous and both chains included the carboxylated form of Lys73 residue. The interaction pattern of the inhibitor in the binding pocket of the two monomers was comparable as well as solvation of the cavity in proximity of Ser70, Tyr211, Thr213, and Arg214. A salt bridge and hydrogen bonds between the positively charged Arg250 and the benzoic acid moiety were observed (Supplementary Figure S3a). The carboxylate group was also oriented in such a way that also Lys208 participated in an ionic bond. Additionally, the distances between the carboxylic oxygen and the hydroxyl group of Thr209 and Ser118 below 3.0 Å were consistent with the formation of hydrogen bonds. Interestingly, the described binding mode resembled the interaction network of the sulfate group of avibactam [61], and the thiolate of SC_2.

Moving on the upper part of the binding pocket (Supplementary Figure S3b), Arg214 established a salt bridge and hydrogen bonds with the 4-COOH group, as well as a cation–π interaction with the phenylethyl moiety of the ligand. The carboxylate group on the pyrazolo[3,4-b]pyridine core was also involved in a water bridge hydrogen bond with Thr213 and Tyr211. Moreover, the central bicyclic aromatic system of the ligand established a π–π interaction with Trp105. Figure 14 and Supplementary Figure S3 highlight the discussed interactions.

Overall, an unambiguous binding mode was obtained consistent with that seen for reversible compound **ID3**, where dianionic moieties clearly engage the positively charged Arg214 and Arg250. The obtained crystal structure therefore proved compound **ID2** to occupy OXA-48 binding pocket with a reversible MoA in line with the aforementioned experimental results.

### 2.8. Determination of MIC, Cytotoxicity and Solubility

To evaluate the capability of restoring β-lactam susceptibility, the two most promising representatives of SC_2 (**ID3**) and SC_7 (**ID2**) were screened alone and in combination with ampicillin (AMP) and IMP against sensitive and resistant Gram-negative bacteria. Avibactam [63] and tazobactam were also included in the MIC characterization as BLI reference compounds. Specifically, we used *E. coli* ATCC25922 as antibiotic-sensitive strain, while *E. coli* ATCC BAA-2523 and *K. pneumoniae* ATCC BAA-2524 as β-lactam-resistant bacteria, producing OXA-48 carbapenemase.

Test compounds and tazobactam were tested at 32 µg/mL, while avibactam was applied at 4 µg/mL. AMP, a member of the penicillin class, was used at increasing concentrations from 1 to 512 μg/mL, while IMP, a carbapenem drug, was applied at 0.03–8 μg/mL. Both antibiotics were selected for the combination study as they can be hydrolyzed by the target OXA-48 enzyme [64].

MIC values were determined by the broth dilution method and interpreted using published guidelines described by Clinical and Laboratory Standards Institute (CLSI) [65,66]. As expected, AMP and IMP showed antibacterial activity against *E. coli* ATCC25922 (IMP was 5-fold more potent than AMP). Both antibiotics displayed reduced potency in β-lactam-resistant strains. Indeed, AMP was completely inactive against *E. coli* ATCC BAA-2523 and *K. pneumoniae* ATCC BAA-2524 (MIC > 512 µg/mL), whereas IMP showed MIC of 2 and 4 µg/mL, respectively, suggesting that AMP is more susceptible to β-lactamase hydrolysis.

Similarly, to reference BLIs, **ID2** and **ID3** showed no antibacterial effect of their own against the Gram-negative pathogens (Table 6). When co-administrated with AMP, **ID2** and **ID3** did not improve the antibiotic activity in resistant bacteria. Tazobactam was also not very effective, while avibactam showed MIC of 16 and 128 µg/mL against *E. coli* and *K. pneumoniae* resistant strains, respectively. On the other hand, tazobactam and avibactam remarkably improved IMP’s activity (from two to more than six-fold) in target resistant bacteria. Notably, even **ID2** decreased the drug’s MIC of two-fold (from 2 to 0.5 µg/mL) in *E. coli* ATCC BAA-2523 but not in *K. pneumoniae* ATCC BAA-2524, whereas **ID3** did not show any significant effects (IMP’s MIC was reduced one-fold only in both resistant strains).

The poor activity of inhibitors **ID2** and **ID3** against β-lactam-resistant bacteria might be ascribed to several factors, such as suboptimal potency against OXA-48 enzyme or poor permeability into the bacterial periplasm. Indeed, despite the reversible inhibitors **ID2** and **ID3** show OXA-48 AC_50_ values comparable to the covalent reference tazobactam, their different MoA and diverse activity in β-lactam-resistant bacteria possibly suggest that stronger OXA-48 inhibition is required for reversible inhibitors to be effective in a whole cell assay.

Also, it is well known that the low permeability barrier of Gram-negative bacteria is a major obstacle in the discovery and development of antibiotics effective against such pathogens. Indeed, compounds with activity against *Gram negative* organisms must overcome further barriers other than the inner membrane and the peptidoglycan layer to function, namely, the penetration of the outer lipid membrane and evasion of efflux pumps [67]. At present no rules exist to predict the permeation of compounds across the cell walls of Gram-negative pathogens; however, understanding the physicochemical property space required for antibacterially active compounds plays a crucial role. In this respect, compounds active against *Gram negative* bacteria are generally characterized by high polarity, which is reflected in low clogD7.4 and high total polar surface area (TPSA) values [68]. When comparing the clogD7.4 and TPSA values of **ID2** (clogD7.4 = 0.19 and TPSA = 116) and **ID3** (clogD7.4 = 0.31 and TPSA = 119) with avibactam (clogD7.4 = −6.77 and TPSA = 139) and tazobactam (clogD7.4 = −5.15 and TPSA = 131) it is evident that our new hits are more lipophilic than the two reference drugs. Also, **ID2** and **ID3** look more lipophilic than most of the compounds active Gram-negative bacteria evaluated by O’Shea and Moser in their analysis of the physicochemical properties of antibacterial agents, where the average clogD7.4 and TPSA values for antibacterials with Gram-negative activity are −2.8 and 165, respectively [68]. Overall, the physicochemical profile of **ID2** and **ID3** might account for their reduced permeability and poor whole cell activity.

Nevertheless, the hit scaffold SC_7 might represent a promising starting point for the development of novel BLIs with whole cell activity. **ID2** could be amenable to structural changes guided by structure-based drug design informed on the available X-ray structures and fine-tuning of physicochemical properties to allow for optimization of the potency and permeability of later-stage lead compounds.

Based on the promising BLI profile of compound **ID2**, a preliminary in vitro ADMET assessment in terms of kinetic solubility and cytotoxicity was carried out. Interestingly, compound **ID2** showed good solubility (179.4 µM) in a buffered aqueous solution at pH 7.4. Also, it was tested for cell viability in dose response against HepG2 cells, following 48 h incubation time [69]. Notably, no sign of toxicity was observed up to the highest tested concentration (100 µM), suggesting a potential safety margin for the BLI activity.

## 3. Materials and Methods

### 3.1. Cheminformatics

All cheminformatics activities were performed using Pipeline Pilot (version 9.5). Chemical group standardization checks in terms of protective group consistency, as well as abbreviations corresponding to chemical structures, tautomer fixing, salt removal, and charge neutralization were carried out to prepare the compound collections. Physicochemical properties were calculated with Chemistry Component collection [70], whereas we used a customized component to monitor structural alerts as reactive groups, REOS and PAINS.

The Generated Fragments Pipeline Pilot component was applied for framework decomposition based on the Bemis-Murcko definition, followed by the InchiKey calculation of the resulting scaffold families to annotate the compounds.

The scaffold distribution of Subset and Full Set collections was analyzed by the CSR curves and quantified with AUC and F_50_ metrics. To generate the CSR curves, the scaffolds were ordered by their frequency of occurrence (most to least common). Then, the cumulative fraction of scaffold is plotted on the X-axis and the cumulative fraction of compounds containing those chemotypes on the Y-axis.

Data analysis and visualization were carried out with Tibco Spotfire (version 7.11.1).

The pKa values of the benzoic acid and phenol group of **ID2** and compounds **5**–**9**, respectively, were calculated using Jaguar software [71] as supplied with the Schrödinger 2019-4 suite. Neutral forms of the ligands were prepared with Ligprep tool [72]. Before pKa calculation, a maximum of five conformers were generated with MacroModel [73] as implemented in the Jaguar tool. The lowest energy conformations were used for pKa prediction maintaining default setting.

### 3.2. Chemistry

#### 3.2.1. General Information

A fresh batch of compounds **ID1**, **ID2**, and **ID3** were purchased from MolPort (Riga, Latvia) for MoA, MIC, X-ray, cytotoxicity, and solubility studies.

Reagents and starting materials were purchased from commercial sources and used as received.

Microwave-assisted reactions were carried out in a Biotage^®^ (Uppsala, Sweden) Initiator + microwave synthesizer. Reaction progress was monitored by thin layer chromatography (TLC, Merck GF254 plates) or LCMS chromatography (Waters X-Select C18, Agilent LCMS system).

Final compounds were purified by flash chromatography on pre-packed silica (230–400 mesh, 40–63 µm) or reverse phase C-18 cartridges using the Combiflash Companion system, when appropriate.

Compound purity and molecular mass were determined by high-pressure liquid chromatography (HPLC) and high-resolution mass spectrometry (UPLC-QTOF). HPLC analysis was carried out through a pump/autosampler (Waters Alliance 2695), a UV photo diode array detector (Waters 2996), and a Waters system data management (Empower 2), using Suplex pkb-100 (250 × 4.6 mm, 5 μm) column. For UPLC-QTOF, an Acquity BEH C18 (2.1 × 50 mm, 1.7 μm) column was used and a positive ion mode in the “VOptics” configuration was employed. Leucine-enkephalin (200 pg/μL) was used as the lock mass in order to provide authenticated exact mass measurement in MS and MS/MS modes within 5 ppm RMS mass accuracy. NMR were obtained using Bruker Avance systems (300, 400, and 500 MHz). All resonance bands were referenced to tetramethylsilane (internal standard). For ^1^H NMR spectroscopy: (s) = singlet; (d) = doublet; (t) = triplet; (q) = quartet; (quin) = quintet; (dd) = double doublet; (dt) = double triplet; (ddd) = double double doublet; (dtd) = double triple doublet; (m) = multiplet; (br) = broad; *J* = coupling constant (Hz); and *δ* = chemical shift (ppm). All compounds tested possessed a purity of ≥95%.

#### 3.2.2. General Procedures

##### General Procedure A—Synthesis of Compounds **16**–**24**, **42**

To a solution of 3-(4-ethylphenyl)-1*H*-pyrazol-5-amine (1 eq.) in EtOH (0.5 M), ethyl-pyruvate or 2-oxopropanoic acid (1 eq.), the proper aldehyde (1 eq.) and conc. HCl (one drop) were added. After heating under MW irradiation at 150 °C for 10–30 min, the reaction mixture was either concentrated under reduced pressure or, in case a precipitated was formed, the solid was filtered off and washed with ice-cold EtOH. Finally, the crude product was purified by the appropriate technique to provide the title compounds.

##### General Procedure B—Synthesis of Compounds **25**–**27**

To a stirred solution of the proper 2,6-dichloropyridine (1 eq.) in THF (0.2 M) at −78 °C, LDA (2 M solution in THF/*n*-heptane/ethylbenzene, 1.1–3 eq.) was added dropwise. After stirring at -78 °C for 30 min, 4-ethylbenzaldehyde (1.1–2.5 eq.) was added dropwise and the resulting mixture was stirred at −78 °C for a further 15 min. The reaction mixture was quenched with an aqueous saturated NH_4_Cl solution, diluted with water, and extracted with EtOAc. The combined organic layers were dried over MgSO_4_, filtered, and evaporated under reduced pressure. The crude was then purified by flash chromatography to provide the title compounds.

##### General Procedure C—Synthesis of Compounds **29**–**31**

To a stirred solution of intermediates **25**, **26**, or **28** (1 eq.) in DCM (0.2 M), Dess-Martin periodinane (1.2 eq.) was added at 0 °C. The resulting mixture was stirred at rt for 15 min. The solids were removed by filtration, then the filtrate was washed with aqueous 1 M NaOH solution and brine, dried over MgSO_4_, filtered and evaporated under reduced pressure. The crude material was then purified by flash chromatography to provide the title compounds.

##### General Procedure D—Synthesis of Compounds **32**–**34**

To a stirred solution of intermediates **29–31** (1 eq.) in EtOH/THF (4:1) (0.5 M), DIPEA (1.05 eq.) was added. The mixture was then cooled to 0 °C and hydrazine (1 M solution in THF, 1.2 eq.) was added dropwise. The resulting solution was stirred at 0 °C for 5 min, then heated at 80 °C for 1.5–16 h. The solvent was evaporated under reduced pressure and the crude product was purified by flash chromatography to provide the title compounds.

##### General Procedure E—Synthesis of Compounds **35**–**37**

A solution of compounds **32**–**34** (1 eq.), (4-(tert-butoxycarbonyl)phenyl)boronic acid (1–1.5 eq.), and K_2_CO_3_ (1.5–2.5 eq.) in dioxane/water (4:1) (0.15 M) was evacuated and purged three times with N_2_. Pd(dppf)Cl_2_·CH_2_Cl_2_ (0.1 eq.) was added and the tube was evacuated and purged three additional times with N_2_. The resulting mixture was heated at 120–140 °C for 1–3 h under MW irradiation then cooled to rt, diluted with EtOAc and water (1:1) and filtered through celite. The phases were separated, and the aqueous layer extracted with EtOAc. The combined organics were washed with water and brine, dried over MgSO_4_, filtered and concentrated under reduced pressure to afford the crude product. The crude was then purified by flash chromatography to provide the title compounds.

##### General Procedure F—Synthesis of Compounds **1**–**9**

To a solution of the intermediates **16**–**24** (1 eq.) in THF/water/MeOH (1:1:1, 0.01–0.05 M), LiOH (1–30 eq.) was added and the resulting mixture was stirred at 40–45 °C for 20–72 h. The reaction mixture was then diluted with water and made acidic to pH~2 by addition of aqueous 1 M HCl solution. The aqueous phase was either directly evaporated under reduced pressure, or first extracted with EtOAc and then dried under vacuum. Alternatively, when precipitation was observed upon acidification, the resulting solid was filtered and washed with water. Both methodologies were applied to afford a residue further purified by the opportune technique to provide the title compounds.

##### General Procedure G—Synthesis of Compounds **10** and **11**

Intermediate **35** or **36** were dissolved in TFA/DCM (1:1, 0.15 M) and the resulting solution was stirred at rt for 16 h. Solvents were removed under vacuum and the residue was purified flash chromatography to provide the title compounds.

#### 3.2.3. Synthesis of Intermediates and Final Compounds

##### 3-(4-Ethylphenyl)-1*H*-pyrazol-5-amine (15)

To a solution of 3-(4-ethylphenyl)-3-oxopropanenitrile (1.43 g, 8.26 mmol) in EtOH (50 mL) hydrazine hydrate (0.97 mL, 9.9 mmol) was added, followed by methanesulfonic acid (0.11 mL, 1.7 mmol). After heating at reflux for 100 min, the reaction mixture was cooled to rt and concentrated under reduced pressure to give a pale-yellow solid. The crude product was purified by flash chromatography on silica gel eluting with 0-10% MeOH/DCM gradient to provide 1.17 g (72% yield) of the title product as a white solid. LCMS m/z: 188 [M + H] +; 1H NMR (400 MHz, CDCl3): δ 7.41–7.35 (m, 2H), 7.18–7.13 (m, 2H), 5.82 (s, 1H), 4.87 (s, 3H), 2.65–2.55 (m, 2H), 1.18 (t, J = 7.6 Hz, 3H).

##### Ethyl-3-(4-ethylphenyl)-6-(3-fluoro-4-(methoxycarbonyl)phenyl)-1*H*-pyrazolo[3,4-b]pyridine-4-carboxylate (16)

The title compound was obtained according to general procedure A, using 3-(4-ethylphenyl)-1*H*-pyrazol-5-amine 15 (200 mg, 1.07 mmol), methyl-2-fluoro-4-formylbenzoate (195 mg, 1.07 mmol) and ethyl-pyruvate (0.12 mL, 1.07 mmol). The crude was recrystallised from water/EtOH to provide 67 mg (14% yield) of the title product. LCMS m/z: 448 [M − H]+. 1H-NMR (400 MHz, DMSO-d6): δ 14.31 (s, 1H), 8.26–8.14 (m, 3H), 8.08 (t, J = 8.0 Hz, 1H), 7.44 (d, J = 8.2 Hz, 2H), 7.36 (d, J = 8.1 Hz, 2H), 4.01–3.88 (m, 5H), 2.70 (q, J = 7.6 Hz, 2H), 1.24 (t, J = 7.6 Hz, 3H), 0.77 (t, J = 7.1 Hz, 3H).

##### Ethyl-3-(4-ethylphenyl)-6-(2-fluoro-4-(methoxycarbonyl)phenyl)-1*H*-pyrazolo[3,4-b]pyridine-4-carboxylate (17)

The title compound was obtained according to general procedure A, using 3-(4-ethylphenyl)-1*H*-pyrazol-5-amine 15 (200 mg, 1.07 mmol), methyl-3-fluoro-4-formylbenzoate (195 mg, 1.07 mmol) and ethyl-pyruvate (0.12 mL, 1.07 mmol). The crude was purified by flash chromatography on silica gel, eluting with 0-50% EtOAc/i-Hex gradient to provide 76 mg (16% yield) of the title product. LCMS m/z: 448 [M − H]+. 1H-NMR (400 MHz, DMSO-d6): δ 14.36 (s, 1H), 8.19 (t, J = 7.9 Hz, 1H), 7.99 (dd, J = 8.1, 1.6 Hz, 1H), 7.93–7.86 (m, 2H), 7.47–7.41 (m, 2H), 7.38–7.33 (m, 2H), 3.99–3.90 (m, 5H), 2.70 (q, J = 7.6 Hz, 2H), 1.24 (t, J = 7.6 Hz, 3H), 0.74 (t, J = 7.1 Hz, 3H).

##### Ethyl-3-(4-ethylphenyl)-6-(3-(methoxycarbonyl)phenyl)-1*H*-pyrazolo[3,4-b]pyridine-4-carboxylate (18)

The title compound was obtained according to general procedure A using 3-(4-ethylphenyl)-1*H*-pyrazol-5-amine 15 (200 mg, 1.07 mmol), methyl-3-formylbenzoate (175 mg, 1.07 mmol) and ethyl-pyruvate (0.12 mL, 1.07 mmol). The crude was purified by flash chromatography on silica gel, eluting with 0-50% EtOAc/i-Hex gradient to provide 125 mg (29% yield) of the title product. LCMS m/z: 430 [M − H]+. 1H-NMR (400 MHz, DMSO-d6): δ 14.26 (s, 1H), 8.85 (app. t, J = 1.8 Hz, 1H), 8.52 (ddd, J = 7.9, 1.9, 1.1 Hz, 1H), 8.11 (ddd, J = 7.7, 1.7, 1.1 Hz, 1H), 8.10 (s, 1H), 7.73 (app. t, J = 7.9 Hz, 1H), 7.46–7.40 (m, 2H), 7.39–7.32 (m, 2H), 3.96 (q, J = 7.1 Hz, 2H), 3.94 (s, 3H), 2.70 (q, J = 7.6 Hz, 2H), 1.24 (t, J = 7.6 Hz, 3H), 0.76 (t, J = 7.1 Hz, 3H).

##### Ethyl-3-(4-ethylphenyl)-6-(4-(2-methoxy-2-oxoethyl)phenyl)-1*H*-pyrazolo[3,4-b]pyridine-4-carboxylate (19)

The title compound was obtained according to general procedure A using 3-(4-ethylphenyl)-1*H*-pyrazol-5-amine 15 (200 mg, 1.07 mmol), methyl-2-(4-formylphenyl)acetate (190 mg, 1.07 mmol) and ethyl-pyruvate (0.12 mL, 1.07 mmol). The crude was recrystallized from EtOAc/i-Hex to give 94 mg (20% yield) of the title product. LCMS m/z: 444 [M − H]+.

##### Ethyl-6-(2,5-difluoro-4-hydroxyphenyl)-3-(4-ethylphenyl)-1*H*-pyrazolo[3,4-b]pyridine-4-carboxylate (20)

The title compound was obtained according to general procedure A 3-(4-ethylphenyl)-1*H*-pyrazol-5-amine 15 (200 mg, 1.07 mmol), 2,5-difluoro-4- hydroxybenzaldehyde (169 mg, 1.07 mmol) and ethyl-pyruvate (0.12 mL, 1.07 mmol). The crude was purified by flash chromatography on silica gel, eluting with 0-50% EtOAc/i-Hex gradient to provide 36 mg (8% yield) of the title product. LCMS m/z: 424 [M − H]+. 1H-NMR (400 MHz, DMSO-d6): δ 14.21 (s, 1H), 11.01 (s, 1H), 7.85 (dd, J = 12.0, 7.4 Hz, 1H), 7.81 (d, J = 1.4 Hz, 1H), 7.44–7.39 (m, 2H), 7.37–7.32 (m, 2H), 6.95 (dd, J = 12.5, 7.3 Hz, 1H), 3.92 (q, J = 7.1 Hz, 2H), 2.69 (q, J = 7.6 Hz, 2H), 1.24 (t, J = 7.6 Hz, 3H), 0.71 (t, J = 7.1 Hz, 3H).

##### Ethyl-6-(3,5-difluoro-4-hydroxyphenyl)-3-(4-ethylphenyl)-1*H*-pyrazolo[3,4-b]pyridine-4-carboxylate (21)

The title compound was obtained according to general procedure A using 3-(4-ethylphenyl)-1*H*-pyrazol-5-amine 15 (300 mg, 1.60 mmol), 3,5-difluoro-4-hydroxybenzaldehyde (253 mg, 1.60 mmol) and ethyl-pyruvate (0.18 mL, 1.60 mmol). The crude was purified by reverse phase chromatography (C-18) eluting with 15-80%, 1% formic acid in water/1% formic acid in AcN to give 202 mg (29% yield) of the title product. LCMS m/z: 424 [M − H]+. 1H-NMR (400 MHz, DMSO-d6): δ 14.13 (s, 1H), 10.84 (s, 1H), 8.03 (s, 1H), 8.01–7.91 (m, 2H), 7.45–7.38 (m, 2H), 7.37–7.32 (m, 2H), 3.94 (q, J = 7.1 Hz, 2H), 2.69 (q, J = 7.6 Hz, 2H), 1.24 (t, J = 7.6 Hz, 3H), 0.76 (t, J = 7.1 Hz, 3H).

##### Ethyl-6-(2,3-difluoro-4-hydroxyphenyl)-3-(4-ethylphenyl)-1*H*-pyrazolo[3,4-b]pyridine-4-carboxylate (22)

The title compound was obtained according to general procedure A using 3-(4-ethylphenyl)-1*H*-pyrazol-5-amine 15 (200 mg, 1.07 mmol), 2,3-difluoro-4-hydroxybenzaldehyde (169 mg, 1.07 mmol) and ethyl-pyruvate (0.12 mL, 1.07 mmol). The crude was purified by flash chromatography on silica gel, eluting with 0-50% EtOAc/i-Hex gradient to give 88 mg (18% yield) of the title product. LCMS m/z: 424 [M − H]+. 1H-NMR (400 MHz, DMSO-d6): δ 14.22 (s, 1H), 10.97 (s, 1H), 7.78 (d, J = 1.7 Hz, 1H), 7.70 (td, J = 8.7, 2.2 Hz, 1H), 7.45–7.40 (m, 2H), 7.37–7.32 (m, 2H), 7.02–6.95 (m, 1H), 3.93 (q, J = 7.1 Hz, 2H), 2.69 (q, J = 7.6 Hz, 2H), 1.24 (t, J = 7.6 Hz, 3H), 0.72 (t, J = 7.1 Hz, 3H).

##### Ethyl-6-(3-chloro-4-hydroxy-5-methoxyphenyl)-3-(4-ethylphenyl)-1*H*-pyrazolo[3,4-b]pyridine-4-carboxylate (23)

The title compound was obtained according to general procedure A using 3-(4-ethylphenyl)-1*H*-pyrazol-5-amine 15 (200 mg, 1.07 mmol), 3-chloro-4-hydroxy-5-methoxybenzaldehyde (199 mg, 1.07 mmol) and ethyl-pyruvate (0.12 mL, 1.07 mmol). The crude was purified by flash chromatography on silica gel, eluting with 0–50% EtOAc/i-Hex and by a further recrystallization from DCM with i-Hex to give 55.5 mg (11% yield) of the title product. LCMS m/z: 452/454 [M − H]+. 1H-NMR (400 MHz, DMSO-d6): δ 14.08 (s, 1H), 9.96 (s, 1H), 8.05 (s, 1H), 7.89 (d, J = 2.1 Hz, 1H), 7.78 (d, J = 2.1 Hz, 1H), 7.44–7.40 (m, 2H), 7.37–7.32 (m, 2H), 3.98 (s, 3H), 3.94 (q, J = 7.1 Hz, 2H), 2.69 (q, J = 7.6 Hz, 2H), 1.24 (t, J = 7.6 Hz, 3H), 0.76 (t, J = 7.1 Hz, 3H).

##### Ethyl-6-(3-chloro-5-fluoro-4-hydroxyphenyl)-3-(4-ethylphenyl)-1*H*-pyrazolo[3,4-b]pyridine-4-carboxylate (24)

The title compound was obtained according to general procedure A using 3-(4-ethylphenyl)-1*H*-pyrazol-5-amine 15 (200 mg, 1.07 mmol), 3-chloro-5-fluoro-4-hydroxybenzaldehyde (186 mg, 1.07 mmol) and ethyl-pyruvate (0.12 mL, 1.07 mmol). The crude was purified by flash chromatography on silica gel, eluting with 0–50% EtOAc/i-Hex and then recrystallized from EtOH/water to give 104 mg (22% yield) of the title product. LCMS m/z: 440/442 [M − H]+.

##### (2,6-Dichloropyridin-3-yl)(4-ethylphenyl)methanol (25)

The title compound was obtained according to general procedure B using 2,6-dichloropyridine (1.02 g, 6.91 mmol), LDA (3.80 mL, 7.60 mmol) and 4-ethylbenzaldehyde (1.04 mL, 7.60 mmol). The crude was purified by flash chromatography on silica gel, eluting with 0–20% i-Hex-EtOAc gradient to provide 1.68 g (82% yield) of the title product. LCMS m/z: 282/284 [M − H]+.

##### (2,6-Dichloro-4-(trifluoromethyl)pyridin-3-yl)(4-ethylphenyl)methanol (26)

The title compound was obtained according to general procedure B using 2,6-dichloro-4-(trifluoromethyl)pyridine (500 mg, 2.32 mmol), LDA (1.45 mL, 2.89 mmol) and 4-ethylbenzaldehyde (0.41 mL, 3.01 mmol). The crude was purified by flash chromatography on silica gel, eluting with 0–20% i-Hex-EtOAc gradient to provide 772 mg (95% yield) of the title product. LCMS m/z: 350/352 [M − H]+.

##### (2,6-Dichloro-4-(hydroxymethyl)pyridin-3-yl)(4-ethylphenyl)methanol (27)

The title compound was obtained according to general procedure B using (2,6-dichloropyridin-4-yl)methanol (956 mg, 5.37 mmol), LDA (8.06 mL, 16.1 mmol) and 4-ethylbenzaldehyde (1.84 mL, 13.4 mmol). The crude was purified by flash chromatography on silica gel, eluting with 0–40% i-Hex-EtOAc to provide 411 mg (23% yield) of the title product. HPLC-MS (ESI) m/z: no ionization.

##### (4-(((Tert-butyldimethylsilyl)oxy)methyl)-2,6-dichloropyridin-3-yl)(4-ethylphenyl)methanol (28)

To a stirred solution of (2,6-dichloro-4-(hydroxymethyl)pyridin-3-yl)(4-ethylphenyl)methanol 27 (394 mg, 1.26 mmol), imidazole (103 mg, 1.51 mmol) and 4-dimethylaminopyridine (15.4 mg, 0.126 mmol) in DCM (20 mL) was added TBDMS-Cl (228 mg, 1.51 mmol) and the resulting solution was stirred at rt for 16 h. The reaction mixture was diluted with DCM (20 mL) and washed with water (3 × 15 mL) and brine (10 mL). The collected organic phases were dried over MgSO4, filtered and evaporated under reduced pressure. The crude material was purified by chromatography by flash chromatography on silica gel, eluting with 0–20% i-Hex-diethyl ether to provide 489 mg (91% yield) of the title compound as a white solid. LCMS m/z: 426/428 [M − H]+.

##### (2,6-Dichloropyridin-3-yl)(4-ethylphenyl)methanone (29)

The title compound was obtained according to general procedure C, using (2,6-dichloropyridin-3-yl)(4-ethylphenyl)methanol 25 (1.58 g, 5.61 mmol) and Dess-Martin periodinane (2.86 g, 6.72 mmol). The crude was purified by flash chromatography on silica gel, eluting with 0–20% i-Hex-EtOAc) to provide 1.29 g (80% yield) of the title product. HPLC-MS m/z: 280/282 [M − H]+.

##### 2,6-Dichloro-4-(trifluoromethyl)pyridin-3-yl)(4-ethylphenyl)methanone (30)

The title compound was obtained according to general procedure C 1 using (2,6-dichloro-4-(trifluoromethyl)pyridin-3-yl)(4-ethylphenyl)methanol 26 (772 mg, 2.21 mmol) and Dess-Martin periodinane (1.12 g, 2.65 mmol). The crude was purified by flash chromatography on silica gel, eluting with 0–20% i-Hex-EtOAc to provide748 mg (99% yield) of the title product. HPLC-MS m/z: 348/350 [M − H]+.

##### (4-(((Tert-butyldimethylsilyl)oxy)methyl)-2,6-dichloropyridin-3-yl)(4-ethylphenyl)methanone (31)

The title compound was obtained according to general C using (4-(((tert-butyldimethylsilyl)oxy)methyl)-2,6-dichloropyridin-3-yl)(4-ethylphenyl)methanol 28 (488 mg, 1.14 mmol) and Dess-Martin periodinane (582 mg, 1.37 mmol). The crude was purified by flash chromatography on silica gel, eluting with 0–20% i-Hex-diethyl-ether to provide 470 mg (96% yield) of the title product. HPLC-MS m/z: 424/426 [M − H]+.

##### 6-Chloro-3-(4-ethylphenyl)-1*H*-pyrazolo[3,4-b]pyridine (32)

The title compound was obtained according to general procedure D, using (2,6-dichloropyridin-3-yl)(4-ethylphenyl)methanone 29 (1.28 g, 4.58 mmol), DIPEA (0.84 mL, 4.8 mmol) and hydrazine (5.5 mL, 5.5 mmol). The crude was purified by flash chromatography on silica gel, eluting with 0–60% i-Hex-EtOAc to provide 995 mg (83% yield) of the title product. HPLC-MS m/z: 258/260 [M − H]+.

##### 6-Chloro-3-(4-ethylphenyl)-4-(trifluoromethyl)-1*H*-pyrazolo[3,4-b]pyridine (33)

The title compound was obtained according to general procedure D, using (2,6-dichloro-4-(trifluoromethyl)pyridin-3-yl)(4-ethylphenyl)methanone 30 (603 mg, 1.73 mmol), DIPEA (0.32 mL, 1.82 mmol) and hydrazine (2.08 mL, 2.08 mmol). The crude was purified by flash chromatography on silica gel, eluting with 0–60% i-Hex-EtOAc to provide 250 mg (43% yield) of the title product. HPLC-MS m/z: 326/328 [M − H]+.

##### 4-(((Tert-butyldimethylsilyl)oxy)methyl)-6-chloro-3-(4-ethylphenyl)-1*H*-pyrazolo[3,4-b]pyridine (34)

The title compound was obtained according to general D, using (4-(((tert-butyldimethylsilyl)oxy)methyl)-2,6-dichloropyridin-3-yl)(4-ethylphenyl)methanone 31 (2.45 g, 5.77 mmol), DIPEA (1.06 mL, 6.06 mmol) and hydrazine (6.93 mL, 6.93 mmol). The crude was purified by flash chromatography on silica gel, eluting with 0–60% i-Hex-EtOAc to provide 1.03 g (43% yield) of the title product. HPLC-MS m/z: 402/404 [M − H]+.

##### Tert-butyl 4-(3-(4-ethylphenyl)-1*H*-pyrazolo[3,4-b]pyridin-6-yl)benzoate (35)

The title compound was obtained according to general procedure E using 6-chloro-3-(4-ethylphenyl)-1*H*-pyrazolo[3,4-b]pyridine 32 (313 mg, 1.22 mmol), (4-(tert-butoxycarbonyl)phenyl)boronic acid (324 mg, 1.46 mmol), K2CO3 (420 mg, 3.04 mmol) and Pd(dppf)Cl2·CH2Cl2 (99 mg, 0.12 mmol). The crude was purified by flash chromatography on silica gel, eluting with 0–30% i-Hex-EtOAc to provide 254 mg (50% yield) of the title product. HPLC-MS m/z: 400 [M − H]+.

##### Tert-butyl-4-(3-(4-ethylphenyl)-4-(trifluoromethyl)-1*H*-pyrazolo[3,4-b]pyridin-6-yl)benzoate (36)

The title compound was obtained according to general procedure E, using 6-chloro-3-(4-ethylphenyl)-4-(trifluoromethyl)-1*H*-pyrazolo[3,4-b]pyridine 33 (250 mg, 0.768 mmol), (4-(tert-butoxycarbonyl)phenyl)boronic acid (205 mg, 0.921 mmol), K2CO3 (265 mg, 1.92 mmol), and Pd(dppf)Cl2·CH2Cl2 (62.7 mg, 0.077 mmol). The crude was purified by flash chromatography on silica gel, eluting with 0–30% i-Hex-EtOAc to provide 191 mg (49% yield) of the title product. HPLC-MS m/z: 468 [M − H]+.

##### Tert-butyl-4-(4-(((tert-butyldimethylsilyl)oxy)methyl)-3-(4-ethylphenyl)-1*H*-pyrazolo[3,4-b]pyridin-6-yl)benzoate (37)

The title compound was obtained according to general procedure E, using 4-(((tert-butyldimethylsilyl)oxy)methyl)-6-chloro-3-(4-ethylphenyl)-1*H*-pyrazolo[3,4-b]pyridine 34 (388 mg, 0.965 mmol), (4-(tert-butoxycarbonyl)phenyl)boronic acid (257 mg, 1.16 mmol), K2CO3 (333 mg, 2.41 mmol), and Pd(dppf)Cl2·CH2Cl2 (79 mg, 0.097 mmol). The crude was purified by flash chromatography on silica gel, eluting with 0–30% i-Hex-EtOAc to provide 335 mg (54% yield) of the title product. HPLC-MS m/z: 544 [M − H]+.

##### Tert-butyl-4-(3-(4-ethylphenyl)-4-(hydroxymethyl)-1*H*-pyrazolo[3,4-b]pyridin-6-yl)benzoate (38)

To a stirred solution of tert-butyl-4-(4-(((tert-butyldimethylsilyl)oxy)methyl)-3-(4-ethylphenyl)-1*H*-pyrazolo[3,4-b]pyridin-6-yl)benzoate 37 (924 mg, 1.70 mmol) in THF (10 mL) was added TBAF (3.4 mL, 3.4 mmol as a 1 M solution in THF) and the resulting solution was stirred at rt for 16 h. The reaction was diluted with DCM (10 mL) and washed with brine (2 × 5 mL). The collected organic layers were dried over MgSO4, filtered, and evaporated under reduced pressure. The crude material was purified by flash chromatography on silica gel, eluting with 0–60% i-Hex-EtOAc to provide 380 mg (52% yield) of the title product as a white solid. HPLC-MS m/z: 430 [M − H]+.

##### Tert-butyl-4-(3-(4-ethylphenyl)-4-formyl-1*H*-pyrazolo[3,4-b]pyridin-6-yl)benzoate (39)

To a stirred solution of tert-butyl-4-(3-(4-ethylphenyl)-4-(hydroxymethyl)-1*H*-pyrazolo[3,4-b]pyridin-6-yl)benzoate 38 (1.01 g, 2.35 mmol) in DCM (5 mL) was added MnO2 (3.07 g, 35.3 mmol) and the resulting mixture was stirred at 95 °C for 16 h. The reaction mixture was filtered through celite. The filtrate was absorbed on silica and purified by flash chromatography on silica gel, eluting with 0–50% i-Hex-EtOAc to provide 817 mg (74% yield) of the title product as a yellow solid. LCMS m/z: 428 [M − H]+. 1H NMR in DMSO-d6 1582-97-P was consistent with product structure at 95% purity.

##### Tert-butyl-4-(3-(4-ethylphenyl)-4-vinyl-1*H*-pyrazolo[3,4-b]pyridin-6-yl)benzoate (40)

To a stirred mixture of methyltriphenylphosphonium bromide (685 mg, 1.92 mmol) in Et2O (10 mL) was added KOtBu (217 mg, 1.93 mmol) at 0 °C and the resulting mixture was stirred at rt for 3 h. Tert-butyl-4-(3-(4-ethylphenyl)-4-formyl-1*H*-pyrazolo[3,4-b]pyridin-6-yl)benzoate 39 (300 mg, 0.702 mmol) was added and the resulting mixture was stirred at rt for a further 16 h. The reaction was absorbed on silica and purified by flash chromatography on silica gel, eluting with 0–40% i-Hex-EtOAc, to provide 232 mg (78% yield) of the title product as a white solid. LCMS m/z: 426 [M − H]+.

##### Tert-butyl-4-(4-ethyl-3-(4-ethylphenyl)-1*H*-pyrazolo[3,4-b]pyridin-6-yl)benzoate (41)

A mixture of tert-butyl-4-(3-(4-ethylphenyl)-4-vinyl-1*H*-pyrazolo[3,4-b]pyridin-6-yl)benzoate 40 (86 mg, 0.20 mmol) and Pd/C (5%, type 87L) (27 mg, 0.10 mmol) in EtOH (2 mL) and AcOH (1 drop) was hydrogenated at 5 bar, rt for 5 h. The reaction was absorbed on silica and purified by flash chromatography on silica gel, eluting with 0–40% i-Hex-EtOAc to provide 86 mg (91% yield) of the title product as a white solid. LCMS m/z: 428 [M − H]+.

##### 3-(4-Ethylphenyl)-6-(4-(methoxycarbonyl)phenyl)-1*H*-pyrazolo[3,4-b]pyridine-4-carboxylic acid (42)

The title compound was obtained according to general procedure A using 3-(4-ethylphenyl)-1*H*-pyrazol-5-amine (230 mg, 1.23 mmol), methyl 4-formylbenzoate (202 mg, 1.23 mmol) and 2-oxopropanoic acid (0.085 mL, 1.23 mmol). A precipitate was filtered off from the reaction mixture and washed with ice-cold EtOH (3 × 1 mL) to provide 91 mg (18% yield) of the title product as a yellow solid. LCMS m/z: 402 [M + H]+. 1H NMR (400 MHz, DMSO-d6): δ 14.20 (s, 1H), 13.76 (s, 1H), 8.47–8.33 (m, 2H), 8.19–8.10 (m, 2H), 8.06 (s, 1H), 7.57–7.48 (m, 2H), 7.31 (d, J = 8.2 Hz, 2H), 3.91 (s, 3H), 2.69 (q, J = 7.5 Hz, 2H), 1.25 (t, J = 7.6 Hz, 3H).

##### Methyl 4-(4-carbamoyl-3-(4-ethylphenyl)-1*H*-pyrazolo[3,4-b]pyridin-6-yl)benzoate (43)

To a solution of 3-(4-ethylphenyl)-6-(4-(methoxycarbonyl)phenyl)-1*H*-pyrazolo[3,4-b]pyridine-4-carboxylic acid 42 (90 mg, 0.22 mmol) and DIPEA (0.196 mL, 1.12 mmol) in DMF (5 mL) was added HATU (102 mg, 0.269 mmol). After 30 min, NH4Cl (36.0 mg, 0.673 mmol) was added and the mixture was stirred at rt for 64 h. HATU (102 mg, 0.269 mmol) was added, and, after stirring for 60 min, NH4Cl (36.0 mg, 0.673 mmol) and DIPEA (0.196 mL, 1.12 mmol) were added sequentially and the mixture was stirred for a further 20 h. The reaction mixture was diluted with water (20 mL) and the resulting precipitate was collected by filtration and washed with water (5 mL). The resulting solid was dissolved in EtOAc (25 mL), dried over MgSO4, filtered and concentrated under reduced pressure. The crude material was purified by flash chromatography on silica gel, eluting with 0–50% THF/Hexane to provide 70 mg (74% yield) of the title product as a white solid. LCMS m/z: 401 [M + H]+. 1H NMR (400 MHz, DMSO-d6): δ 14.14 (s, 1H), 8.53–8.39 (m, 2H), 8.20 (dd, J = 8.6, 1.9 Hz, 3H), 7.94 (s, 1H), 7.87 (s, 1H), 7.73–7.64 (m, 2H), 7.35 (d, J = 8.2 Hz, 2H), 3.97 (s, 3H), 2.74 (q, J = 7.6 Hz, 2H), 1.31 (t, J = 7.6 Hz, 3H).

##### 6-(4-Carboxy-3-fluorophenyl)-3-(4-ethylphenyl)-1*H*-pyrazolo[3,4-b]pyridine-4-carboxylic acid (1)

The title compound was obtained according to general procedure F, using ethyl-3-(4-ethylphenyl)-6-(3-fluoro-4-(methoxycarbonyl)phenyl)-1*H*-pyrazolo[3,4-b]pyridine-4-carboxylate 16 (67 mg, 0.15 mmol) and LiOH (17.93 mg, 0.749 mmol). The resulting solid was collected by filtration, washed with water and dried under vacuum to provide 49 mg (77% yield) of the title product. 1H NMR (DMSO-d6, 500 MHz): δ 14.19 (br s, 1H), 13.58 (br s, 2H), 8.20–8.10 (m, 2H), 8.07 (s, 1H), 8.03 (t, J = 7.9 Hz, 1H), 7.52 (d, J = 7.9 Hz, 2H), 7.30 (d, J = 7.9 Hz, 2H), 2.68 (q, J = 7.6 Hz, 2H), 1.24 (t, J = 7.6 Hz, 3H); 13C NMR (125 MHz, DMSO-d6): δ 168.0, 165.2, 162.0, 154.0, 153.5, 144.8, 144.4, 144.0, 138.1, 133.1, 131.5, 128.9, 127.9, 123.4, 120.5, 115.8, 113.7, 108.1, 28.4, 16.0; HPLC-MS m/z: 406 [M + H]+; HRMS m/z: calcd for C22H16FN3O4 [M + H]+ 406.1198, found 406.1201.

##### 6-(4-Carboxy-2-fluorophenyl)-3-(4-ethylphenyl)-1*H*-pyrazolo[3,4-b]pyridine-4-carboxylic acid (2)

The title compound was obtained according to general procedure F, using ethyl-3-(4-ethylphenyl)-6-(2-fluoro-4-(methoxycarbonyl)phenyl)-1*H*-pyrazolo[3,4-b]pyridine-4-carboxylate 17 (72 mg, 0.16 mmol) and LiOH (40 mg, 1.7 mmol). The resulting solid was collected by filtration, washed with water/TBME/i-Hex (2.5:1:2.5) and dried under vacuum to provide 62 mg (94% yield) of the title product. 1H NMR (500 MHz, DMSO-d6): δ 14.27 (br s, 1H), 13.58 (br s, 1H), 8.16 (t, J = 7.9 Hz, 1H), 7.96 (dd, J = 1.4, 8.2 Hz, 1H), 7.89–7.80 (m, 2H), 7.52 (d, J = 7.9 Hz, 2H), 7.31 (d, J = 8.1 Hz, 2H), 2.69 (q, J = 7.5 Hz, 2H), 1.25 (t, J = 7.6 Hz, 3H); 13C NMR (125 MHz, DMSO-d6): δ 167.8, 166.4, 160.2, 153.9, 151.6, 144.8, 144.0, 137.3, 134.3, 132.0, 131.6, 130.8, 129.0, 127.8, 126.1, 117.6, 116.8, 107.7, 28.4, 16.0; HPLC-MS m/z: 406 [M + H]+; HRMS m/z: calcd for C22H16FN3O4 [M + H]+ 406.1198, found 406.1204.

##### 6-(3-Carboxyphenyl)-3-(4-ethylphenyl)-1*H*-pyrazolo[3,4-b]pyridine-4-carboxylic acid (3)

The title compound was obtained according to general procedure F, using ethyl-3-(4-ethylphenyl)-6-(3-(methoxycarbonyl)phenyl)-1*H*-pyrazolo[3,4-b]pyridine-4-carboxylate 18 (122 mg, 0.273 mmol) and LiOH (6.5 mg, 0.27 mmol). The resulting solid was collected by filtration, washed with water/TBME (2:1) and dried under vacuum to provide 90.5 mg (85% yield) of the title product. 1H NMR (500 MHz, DMSO-d6): δ 14.16 (br s, 1H), 13.94–12.94 (m, 2H), 8.83 (s, 1H), 8.47 (d, J = 7.9 Hz, 1H), 8.08 (d, J = 7.8 Hz, 1H), 8.04 (s, 1H), 7.70 (t, J = 7.8 Hz, 1H), 7.52 (d, J = 8.1 Hz, 2H), 7.31 (d, J = 8.1 Hz, 2H), 2.69 (q, J = 7.6 Hz, 2H), 1.25 (t, J = 7.6 Hz, 3H); 13C NMR (125 MHz, DMSO-d6): δ 168.0, 167.6, 155.1, 154.2, 144.7, 143.9, 138.7, 137.6, 132.0, 131.9, 131.7, 130.9, 129.9, 128.9, 128.4, 127.9, 113.4, 107.7, 28.4, 16.0; HPLC-MS m/z: 388 [M + H]+; HRMS m/z: calcd for C22H17N3O4 [M + H]+ 388.1292, found 388.1287.

##### 6-(4-(Carboxymethyl)phenyl)-3-(4-ethylphenyl)-1*H*-pyrazolo[3,4-b]pyridine-4-carboxylic acid (4)

The title compound was obtained according to general procedure F, using ethyl-3-(4-ethylphenyl)-6-(4-(2-methoxy-2-oxoethyl)phenyl)-1*H*-pyrazolo[3,4-b]pyridine-4-carboxylate 19 (94 mg, 0.21 mmol) and LiOH (30.5 mg, 1.27 mmol). The resulting solid was collected by filtration, washed with water and dried under vacuum to provide 74.8 mg (87% yield) of the title product. 1H NMR (500 MHz, DMSO-d6): δ 14.07 (br s, 1H), 13.80–12.23 (m, 2H), 8.18 (d, J = 8.1 Hz, 2H), 7.95 (s, 1H), 7.51 (d, J = 8.1 Hz, 2H), 7.45 (d, J = 8.2 Hz, 2H), 7.30 (d, J = 7.9 Hz, 2H), 3.68 (s, 2H), 2.68 (q, J = 7.5 Hz, 2H), 1.25 (t, J = 7.6 Hz, 3H); 13C NMR (125 MHz, DMSO-d6): δ 173.0, 168.1, 156.0, 154.2, 144.6, 143.9, 137.4, 137.3, 136.8, 131.8, 130.5, 128.9, 127.8, 127.6, 113.3, 107.3, 40.9, 28.4, 16.0; HPLC-MS m/z: 402 [M + H]+; HRMS m/z: calcd for C23H19N3O4 [M + H]+ 402.1448, found 402.1439.

##### 6-(2,5-Difluoro-4-hydroxyphenyl)-3-(4-ethylphenyl)-1*H*-pyrazolo[3,4-b]pyridine-4-carboxylic acid (5)

The title compound was obtained according to general procedure F, using ethyl-6-(2,5-difluoro-4-hydroxyphenyl)-3-(4-ethylphenyl)-1*H*-pyrazolo[3,4-b]pyridine-4-carboxylate 20 (35 mg, 0.083 mmol) and LiOH (19.8 mg, 0.827 mmol). The resulting solid was collected by filtration, washed with water/Hex (1:2) and dried under vacuum to provide 25 mg (75% yield) of the title product. 1H NMR (500 MHz, DMSO-d6): δ 14.11 (br s, 1H), 13.68 (br s, 1H), 10.95 (br s, 1H), 7.84 (dd, J = 7.3, 11.9 Hz, 1H), 7.77 (d, J = 0.9 Hz, 1H), 7.49 (d, J = 8.1 Hz, 2H), 7.30 (d, J = 8.1 Hz, 2H), 6.95 (dd, J = 7.3, 12.4 Hz, 1H), 2.68 (q, J = 7.6 Hz, 2H), 1.24 (t, J = 7.6 Hz, 3H); 13C NMR (125 MHz, DMSO-d6): δ 167.8, 156.8, 153.9, 151.7, 148.1, 148.5, 144.7, 143.9, 136.8, 131.7, 128.9, 127.8, 117.3, 117.1, 116.3, 107.1, 106.1, 28.4, 16.0; HPLC-MS m/z: 396 [M + H]+; HRMS m/z: calcd for C21H15N3O3 [M + H]+ 396.1154, found 396.1153.

##### 6-(3,5-Difluoro-4-hydroxyphenyl)-3-(4-ethylphenyl)-1*H*-pyrazolo[3,4-b]pyridine-4-carboxylic acid (6)

The title compound was obtained according to general procedure F, using ethyl-6-(3,5-difluoro-4-hydroxyphenyl)-3-(4-ethylphenyl)-1*H*-pyrazolo[3,4-b]pyridine-4-carboxylate 21 (191 mg, 0.451 mmol) and LiOH (108 mg, 4.51 mmol). The crude was crystallized from EtOH to provide 51 mg (27% yield) of the title product. 1H NMR (300 MHz, DMSO-d6): δ 14.03 (br s, 1H), 13.65 (br s, 1H), 10.72 (s, 1H), 8.02–7.87 (m, 3H), 7.50 (d, J = 8.1 Hz, 2H), 7.30 (d, J = 8.3 Hz, 2H), 2.68 (q, J = 8.0 Hz, 1H), 1.24 (t, J = 7.6 Hz, 3H); 13C NMR (125 MHz, DMSO-d6): δ 168.1, 153.9, 153.0, 144.6, 143.9, 137.5, 135.9, 131.6, 129.2, 128.8, 128.4, 127.9, 112.8, 111.1, 107.3, 28.4, 16.0; HPLC-MS m/z: 396 [M + H]+; HRMS m/z: calcd for C21H15N3O3 [M + H]+ 396.1154, found 396.1152.

##### 6-(2,3-Difluoro-4-hydroxyphenyl)-3-(4-ethylphenyl)-1*H*-pyrazolo[3,4-b]pyridine-4-carboxylic acid (7)

The title compound was obtained according to general procedure F, using ethyl-6-(2,3-difluoro-4-hydroxyphenyl)-3-(4-ethylphenyl)-1*H*-pyrazolo[3,4-b]pyridine-4-carboxylate 22 (85 mg, 0.20 mmol) and LiOH (48.1 mg, 2.01 mmol). The resulting solid was collected by filtration, washed with water/ i-Hex (1:2) and dried under vacuum to provide 62.5 mg (78% yield) of the title product. 1H NMR (400 MHz, DMSO-d6): δ 14.21 (br s, 1H), 13.77 (br s, 1H), 11.00 (br s, 1H), 7.80 (d, J = 1.8 Hz, 1H), 7.75 (dt, J = 2.2, 8.7 Hz, 1H), 7.55 (d, J = 8.2 Hz, 2H), 7.36 (d, J = 8.3 Hz, 2H), 7.10–6.98 (m, 1H), 2.74 (q, J = 7.6 Hz, 2H), 1.30 (t, J = 7.6 Hz, 3H); 13C NMR (125 MHz, DMSO-d6): δ 167.8, 153.9, 152.1, 150.0, 148.5, 144.7, 143.9, 140.4, 136.8, 131.7, 128.9, 127.8, 125.2, 118.8, 116.2, 113.8, 107.1, 28.4, 16.0; HPLC-MS m/z: 396 [M + H]+; HPLC-MS m/z: 396 [M + H]+; HRMS m/z: calcd for C21H15N3O3 [M + H]+ 396.1154, found 396.1153.

##### 6-(3-Chloro-4-hydroxy-5-methoxyphenyl)-3-(4-ethylphenyl)-1*H*-pyrazolo[3,4-b]pyridine-4-carboxylic acid (8)

The title compound was obtained according to general F, using ethyl-6-(3-chloro-4-hydroxy-5-methoxyphenyl)-3-(4-ethylphenyl)-1*H*-pyrazolo[3,4-b]pyridine-4-carboxylate 23 (53 mg, 0.12 mmol) and LiOH (28 mg, 1.169 mmol). The resulting solid was collected by filtration, washed with water/Hex (1:2) and dried under vacuum to provide 41 mg (82% yield) of the title product. 1H NMR (400 MHz, DMSO-d6): δ 14.03 (s, 1H), 13.70 (br s, 1H), 9.97 (s, 1H), 8.01 (s, 1H), 7.89 (d, J = 2.0 Hz, 1H), 7.78 (d, J = 2.0 Hz, 1H), 7.51 (d, J = 8.2 Hz, 2H), 7.31 (d, J = 8.3 Hz, 2H), 3.99 (s, 3H), 2.68 (q, J = 7.6 Hz, 2H), 1.25 (t, J = 7.6 Hz, 3H); 13C NMR (125 MHz, DMSO-d6): δ 168.2, 154.9, 154.0, 149.4, 145.1, 144.5, 143.9, 137.4, 131.7, 129.7, 128.8, 127.9, 121.1, 120.9, 112.8, 109.6, 107.0, 56.9, 28.4, 16.0; HPLC-MS m/z: 424.0 [M + H]+; HRMS m/z: calcd for C22H18ClN3O4 [M + H]+ 424.1059, found 424.1054.

##### 6-(3-Chloro-5-fluoro-4-hydroxyphenyl)-3-(4-ethylphenyl)-1*H*-pyrazolo[3,4-b]pyridine-4-carboxylic acid (9)

The title compound was obtained according to general procedure F, using ethyl-6-(3-chloro-5-fluoro-4-hydroxyphenyl)-3-(4-ethylphenyl)-1*H*-pyrazolo[3,4-b]pyridine-4-carboxylate 24 (104 mg, 0.236 mmol) and LiOH (57.6 mg, 2.36 mmol). The resulting solid was collected by filtration, washed with water and dried under vacuum to provide 86 mg (87% yield) of the title product. 1H NMR (500 MHz, DMSO-d6): δ 14.05 (br s, 1H), 13.70 (br s, 1H), 10.95 (br s, 1H), 8.14 (s, 1H), 8.08 (dd, J = 2.1, 12.0 Hz, 1H), 7.99 (s, 1H), 7.50 (d, J = 8.1 Hz, 2H), 7.30 (d, J = 8.1 Hz, 2H), 2.68 (q, J = 7.6 Hz, 2H), 1.24 (t, J = 7.6 Hz, 3H); 13C NMR (125 MHz, DMSO-d6): δ 168.1, 153.9, 153.7, 152.6, 144.6, 143.9, 143.6, 137.6, 131.6, 130.0, 128.8, 127.9, 124.5, 123.1, 114.0, 112.8, 107.3, 28.4, 16.0; HPLC-MS m/z: 412 [M + H]+.

##### 4-(3-(4-Ethylphenyl)-1*H*-pyrazolo[3,4-b]pyridin-6-yl)benzoic acid (10)

The title compound was obtained according to general procedure G, using tert-butyl 4-(3-(4-ethylphenyl)-1*H*-pyrazolo[3,4-b]pyridin-6-yl)benzoate 35 (252 mg, 0.631 mmol), TFA/DCM (1:1, 4 mL). The crude was purified by flash chromatography on silica gel, eluting with 0–100% i-Hex-EtOAc with1% AcOH, to provide 95 mg (43% yield) of the title product. 1H NMR (300 MHz, DMSO-d6): δ 13.86 (br s, 1H), 13.10 (br s, 1H), 8.67 (d, J = 8.6 Hz, 1H), 8.32 (d, J = 8.7 Hz, 2H), 8.11 (d, J = 8.6 Hz, 2H), 7.98 (d, J = 8.3 Hz, 2H), 7.94 (d, J = 8.6 Hz, 1H), 7.39 (d, J = 8.3 Hz, 2H), 2.69 (q, J = 7.6 Hz, 2H), 1.25 (t, J = 7.6 Hz, 3H); 13C NMR (75 MHz, DMSO-d6): δ 167.1, 154.2, 153.2, 144.0, 142.8, 142.4, 131.6, 131.5, 130.6, 129.9, 128.4, 127.3, 126.5, 114.9, 111.5, 28.0, 15.6; HPLC-MS m/z: 344 [M + H]+; HRMS m/z: calcd for C21H17N3O2 [M + H]+ 344.1393, found 344.1381.

##### 4-(3-(4-Ethylphenyl)-4-(trifluoromethyl)-1*H*-pyrazolo[3,4-b]pyridin-6-yl)benzoic acid (11)

The title compound was obtained according to general procedure G, using tert-butyl-4-(3-(4-ethylphenyl)-4-(trifluoromethyl)-1*H*-pyrazolo[3,4-b]pyridin-6-yl)benzoate 36 (190 mg, 0.406 mmol), TFA/DCM (1:1, 4 mL). The crude was purified by flash chromatography on silica gel, eluting with 0-100% i-Hex-EtOAc with 1% AcOH to provide 58 mg (34% yield) of the title product. 1H NMR (300 MHz, DMSO-d6): δ 14.49 (br s, 1H), 13.17 (br s, 1H), 8.46–8.34 (m, J = 8.6 Hz, 2H), 8.18 (s, 1H), 8.16–8.08 (m, J = 8.6 Hz, 2H), 7.47–7.40 (m, 2H), 7.36–7.30 (m, 2H), 2.71 (q, J = 7.7 Hz, 2H), 1.26 (t, J = 7.6 Hz, 3H); 13C NMR (75 MHz, DMSO-d6): δ 166.9, 154.8, 153.5, 143.9, 141.0, 132.1, 130.8, 130.7, 129.8, 129.4, 128.0, 127.5, 127.2, 122.0, 111.6, 106.5, 27.9, 15.4; HPLC-MS m/z: 412 [M + H]+; HRMS m/z: calcd for C22H16F3N3O2 [M + H]+ 412.1267, found 412.1264.

##### 4-(3-(4-Ethylphenyl)-4-(hydroxymethyl)-1*H*-pyrazolo[3,4-b]pyridin-6-yl)benzoic acid (12)

The title compound was obtained according to general procedure H, using tert-butyl-4-(3-(4-ethylphenyl)-4-(hydroxymethyl)-1*H*-pyrazolo[3,4-b]pyridin-6-yl)benzoate 38 (70 mg, 0.16 mmol) and 4 M HCl in 1,4-dioxane (407 µL, 1.63 mmo). The crude was purified by flash chromatography on silica gel, eluting with 0-80% i-Hex-EtOAc with 1% AcOH to provide 41 mg (65% yield) of the title product. 1H NMR (300 MHz, DMSO-d6): δ 13.82 (br s, 1H), 13.09 (br s, 1H), 8.27 (d, J = 8.4 Hz, 2H), 8.11 (d, J = 8.4 Hz, 2H), 7.94 (s, 1H), 7.58 (d, J = 8.1 Hz, 2H), 7.35 (d, J = 8.2 Hz, 2H), 5.50 (br s, 1H), 4.73 (s, 2H), 2.71 (q, J = 7.6 Hz, 2H), 1.26 (t, J = 7.6 Hz, 3H); 13C NMR (125 MHz, DMSO-d6): δ 167.6, 155.0, 153.4, 148.4, 144.9, 144.4, 143.2, 132.3, 131.9, 130.4, 129.7, 128.1, 127.6, 111.5, 110.2, 60.5, 28.4, 16.0; HPLC-MS m/z: 374 [M + H]+; HRMS m/z: calcd for C22H19N3O3 [M + H]+ 374.1499, found 374.1497.

##### 4-(4-Ethyl-3-(4-ethylphenyl)-1*H*-pyrazolo[3,4-b]pyridin-6-yl)benzoic acid (13)

The title compound was obtained according to general procedure H, using tert-butyl-4-(4-ethyl-3-(4-ethylphenyl)-1*H*-pyrazolo[3,4-b]pyridin-6-yl)benzoate 41 (85 mg, 0.20 mmol) and 4 M HCl in 1,4-dioxane (0.5 mL, 2.0 mmol). The crude was purified by flash chromatography on silica gel, eluting with 0–100% i-Hex-EtOAc with 1% AcOH to provide 23 mg (31% yield) of the title product. 1H NMR (300 MHz, DMSO-d6): δ 13.77 (br s, 1H), 13.07 (br s, 1H), 8.31 (d, J = 8.5 Hz, 2H), 8.09 (d, J = 8.4 Hz, 2H), 7.72 (s, 1H), 7.55 (d, J = 8.1 Hz, 2H), 7.36 (d, J = 8.1 Hz, 2H), 2.88 (q, J = 7.6 Hz, 2H), 2.71 (q, J = 7.5 Hz, 2H), 1.26 (t, J = 7.6 Hz, 3H), 1.05 (t, J = 7.5 Hz, 3H); 13C NMR (125 MHz, DMSO-d6): δ 167.6, 154.9, 153.6, 149.8, 145.2, 144.3, 143.2, 132.5, 131.9, 130.2, 129.9, 128.1, 127.7, 114.4, 112.0, 28.4, 26.0, 16.0, 14.6; HPLC-MS m/z: 372 [M + H]+; HRMS m/z: calcd for C23H21N3O2 [M + H]+ 372.1706, found 372.1708.

##### 4-(4-Carbamoyl-3-(4-ethylphenyl)-1*H*-pyrazolo[3,4-b]pyridin-6-yl)benzoic acid (14)

To a solution of methyl 4-(4-carbamoyl-3-(4-ethylphenyl)-1*H*-pyrazolo[3,4-b]pyridin-6-yl)benzoate 43 (70 mg, 0.18 mmol) in MeOH (1 mL), THF (1 mL), water (1 mL) was added LiOH (32 mg, 1.4 mmol). After stirring at rt for 4 h, the mixture was diluted with water (5 mL), acidified to pH 2 by addition of 2 M aqueous HCl, filtered and washed with water (2 × 1 mL). The crude product was slurred in H2O/EtOH (1:2, 5 mL) for 4 h then filtered and washed with ice cold EtOH (2 × 1 mL) to provide 15.5 mg (23% yield) of the title product as a white solid. 1H NMR (400 MHz, DMSO-d6): δ 14.07 (s, 1H), 13.14 (s, 1H), 8.45–8.29 (m, 2H), 8.18–8.05 (m, 3H), 7.87 (s, 1H), 7.81 (s, 1H), 7.66–7.58 (m, 2H), 7.29 (d, J = 8.2 Hz, 2H), 2.68 (q, J = 7.6 Hz, 2H), 1.25 (t, J = 7.6 Hz, 3H); 13C NMR (125 MHz, DMSO-d6): δ 168.8, 167.5, 154.7, 154.1, 144.6, 143.9, 142.5, 141.7, 132.0, 131.4, 130.4, 128.8, 127.8, 127.8, 113.0, 107.9, 28.4, 15.9; LCMS m/z: 387 [M + H]+; HRMS m/z: calcd for C22H18N4O3 [M + H]+ 387.1452, found 387.1430.

### 3.3. Protein Production and Purification

The blaOXA gene coding for the OXA-48 ß-lactamase, originally cloned from the *K. pneumonia* 11978 (a clinical isolate from Istanbul Faculty Hospital, Istanbul, Turkey) was overexpressed via a T7 expression system in *E. coli* BL21(DE3) [74,75], and its product, native protein, (final yield of 30 mg/L of culture) was subjected to several purification steps, including ion-exchange chromatography with Q-Sepharose and 20 mM Tris-HCl buffer (pH 9.0), followed by chromatography with S-Sepharose columns and 100 mM sodium phosphate buffer (pH 5.8). Elution of the beta-lactamase was performed with a K_2_SO_4_ gradient. Peaks of beta-lactamase activity were concentrated and were dialyzed with 100 mM phosphate buffer (pH 7.0). The OXA-48 enzyme was purified to homogeneity (with a degree of purity around 95.4%.)-as measured by 12% SDS PAGE [23]. OXA-48 protein stock solution (2 mg/mL) was finally prepared in 20 mM Hepes (pH 8,1). OXA-48 β-lactamase specific activity was measured by following the hydrolysis of 100 μM nitrocefin (ε: 17,000 M-1 cm^−1^ at 482 nm; Δε: 15,000 M-1 cm^−1^ at 482 nm) and corresponding to 60.45 μmoles min^−1^ mg^−1^ of OXA-48 (kcat = 28.85 s^−1^) enzyme. A MW of 28,147 g/mol, calculated from the enzyme sequence was confirmed by MALDI-TOF analysis with a Mass error of ± 2.8 Da.

### 3.4. X-ray Crystallography

OXA-48 crystals with **ID2** and **ID3** compounds were grown using the sitting drop vapor diffusion method. The protein, starting from a solution Hepes 20 mM pH 8.1, was concentrated up to 7.5–8.5 mg/mL using Amicon Ultra (Merck) centrifugal concentrators and the ligands **ID2** and **ID3** added in large excess (6.5:1) and incubated overnight at 4 °C. A further step of purification was performed by gel filtration (Superdex 75 10/300 GL column; GE Healthcare) in buffer 20 mM HEPES pH 7.5 to remove the excess of the ligands. Crystals of the two complexes were obtained after few days by the vapor diffusion method at 18 °C using a sitting-drop setup made by mixing 2 μL of protein solution (7.5–8.5 mg/mL, in 20mM HEPES, pH 7.5) with 2 μL of reservoir solution (0.1 M HEPES, pH 7.5, 12% PEG8000, 8% 1-butanol). The best crystals were flash cooled in liquid nitrogen after cryoprotection. X-ray data were collected at 100 K on the ID29 beamlines at the European Synchrotron Radiation Facility (ESRF; Grenoble; France). Crystals of OXA-48 in complex with **ID2** and **ID3** belong to the space group P2_1_2_1_2_1_ and diffracted to a maximum resolution of 1.65 and 2.1 Å, respectively (Supplementary Table S3). The collected data were processed using the programs MOSFLM/SCALA [76]. Crystal structures were solved with AMoRe [77], using as starting model the coordinates of OXA-48 apo structure (PDB code: 3HBR). The coordinates were then refined with CNS [78] and a final step of refinement was performed with the software Phenix [79]. Statistics of crystallographic data and refinement are summarized in Table S3. The atomic coordinates and structure factors have been deposited in the Protein Data Bank, www.pdb.org (PDB code 7AUX and 7AW5 for **ID2** and **ID3**, respectively).

The two complexes were processed using the Protein Preparation Wizard tool supplied with the Schrödinger modeling suite [80,81]. Fully atom-typed structures were obtained with default setting calculation. Figure 13 and Figure 14, and Supplementary Figures S2 and S3 were made using VMD 1.9.4 [82].

### 3.5. Primary Enzymatic Assay for OXA-48

The primary assay was developed to monitor the enzymatic activity of OXA-48 taking advantage of the spectroscopic properties of white microtiter plates (Optiplate-384, PerkinElmer, #6007290), as indicated by Zuck et al. [83]. Nitrocefin is chromogenic substrate for beta-lactamases with λ_max_ = 395 nm (yellow). Upon hydrolysis by OXA-48, nitrocefin yields (2R)-2-[(R)-carboxy[2-(thiophen-2-yl)acetamido]methyl]-5-[(E)-2-(2,4-dinitrophenyl)ethenyl]-3,6-dihydro-2*H*-1,3-thiazine-4-carboxylic acid, with increased absorbance properties at λ_max_ = 495 nm. Spectroscopic features of the reaction product are exploited to quench the plate intrinsic fluorescence by absorbing the plate’s emission light, which can be monitored by exciting the reaction at 485 nm and recording the emission at 520 nm. Therefore, the primary assay for HTS was an ON-assay, in which compounds targeting OXA-48 were predicted to elicit an increase of the fluorescence signal.

The primary assay was performed in reaction buffer (20 mM Hepes pH 8.0, 12.5 mM NaCl, 0.005% BSA, 0.0005% Brij-35) in the presence of 0.0014 ng/µL OXA-48 and 25 µM nitrocefin in a final volume of 20 µL/well in 384 MTP format. If not otherwise indicated, compounds (50 mM stock solutions in 100% DMSO) were stepwise diluted to 0.5% DMSO in reaction buffer and preincubated with OXA-48 for 15 min before the reaction start with nitrocefin. Reactions were read in kinetics or as endpoint after 60 min at rt. The addition steps of the automated protocol applied for the screening campaign were accomplished by a CyBi^®^-Well Vario dispensing units integrated in the automated screening station.

### 3.6. Counter-Screening Assay

The counter-screening was implemented as negative selection of true-positive hit compounds targeting OXA-48 with the identification of putative false-positive hits interfering with the corresponding readout system, i.e., compounds displaying an autofluorescent signal which was not quenched by the reaction product of nitrocefin hydrolysis. Counter-screening assay was run after the HC phase and also after the HE phase, to filter out compounds with possible off-target interfering behavior.

Basically, the enzymatic reaction was assembled by adding first the OXA-48 enzyme mix and the nitrocefin substrate mix under the same working concentrations and experimental conditions described for the primary assay for HTS, but in the absence of the compounds to be analyzed. After 60 min of enzymatic reaction, plate fluorescence was measured at 520 nm upon excitation at 485 nm before and after the injection of selected compounds at the equivalent concentration applied with the primary assay. The assay was performed with interplate triplicates. Hit compounds which confirmed their activity with the counter-screening assay were flagged as putative false-positive hits and excluded from the subsequent selection.

### 3.7. HTS Campaign Mathematical Background

Raw data from the primary enzymatic assay were normalized versus neutral and inhibitor controls in order to obtain the activity [%] using the following formula (1)

(1) $$Activity\;[\%]=-100*(\frac{x-<NeutralControls>}{<InhibitorControls>-<NeutralControls>})$$

where:

x is the calculated signal value of a well

< NeutralControl > is the median of the calculated signal values for the central reference wells of a plate (median of enzymatic reaction occurred in presence of DMSO)

< InhibitorControl > is the median of the calculated signal values for the scale reference wells of a plate (median of enzymatic reaction occurred in presence of tazobactam IC_100_)

The activity [%] was used for computation of Robust Z Prime Factor (RZ’ Factor) using formula (2). It is based on the same formulas as the Z’ Factor [51], but standard deviations and means are replaced by the Robust Standard Deviations (RSD) and medians (“< >”), respectively.

(2) $$RZ^{\prime }=1-\frac{3*(RSD_{NeutralControls}+RSD_{inhibitorControls}}{Abs(\langle NeutralConttrols\rangle -\langle InhibitorControls\rangle }$$

RZ’ Factor was used as plate quality criteria, including in the analysis only those plates having a RZ’ > 0.5.

### 3.8. Mechanism of Action of OXA-48 Inhibiotors

The nature of the inhibition of the compounds was studied on the OXA-48 primary assay to assess the reversibility of the inhibition mechanism by analyzing a subset of test compounds for their time-dependent inhibition of OXA-48 and their ability to retain their inhibition properties in dilution-after-preincubation experiments.

#### 3.8.1. Time-Dependent Inhibition of OXA-48

In order to investigate whether the compounds acted as reversible or non-reversible inhibitors of the OXA-48 catalytic activity, dose–response curve of reference inhibitor tazobactam (10 concentrations from 46.2 μM to 0.001 μM; fold dilution 1:3.16) and test compounds **ID1** and **ID2** (10 concentrations from 100 μM to 0.0032 μM; fold dilution 1:3.16) were preincubated with OXA-48 enzyme to measure the time-dependent inhibition of each compound. Experimental conditions were identical to the ones applied for the primary assay for HTS: 20 μL of 2X compounds with 1% DMSO; 10 μL 4X enzyme (0.0014 ng/µL OXA-48 final concentration) were dispensed into preincubation plate. The reaction buffer was: 20 mM Hepes pH 8.0, 12.5 mM NaCl, 0.005% BSA-pf, 0.0005% Brij-35.

Preincubation time was for a period spanning from 0 up to 240′ at rt between enzyme and compounds and for 15′ at rt between enzyme and control wells. The reactions were started by transferring of 15 μL of enzyme-compounds or enzyme-control from preincubation plate into assay plate adding 5 μL 4X nitrocefin substrate (25 µM final concentration) at different time points, at 0 (i.e., no preincubation)–15–30–60–90–120–180–240 min of preincubation between OXA-48 and compounds. Reading was at PHERAstar (BMG Labtechnologies), in kinetic for up to 60′ (λex: 485 nm; λem: 520 nm). Data were analyzed to determine the respective AC_50_ values obtained at all the tested preincubation time points.

a. If the AC_50_ value was independent on the preincubation time with OXA-48, the compound was assigned as reversible inhibitor.

b. If the AC_50_ value was inversely proportional to the preincubation time with OXA-48, the compound was assigned as non-reversible inhibitor.

#### 3.8.2. Dilution after Preincubation of OXA-48 with Selected Inhibitors

In order to validate the results obtained in the time-dependent inhibition assay, dose–response curves of reference inhibitor tazobactam, test compounds and enzyme mix were prepared as above described and 5-fold concentrated. Compounds (20 μL of 10X compounds) and enzyme (10 μL 20X enzyme in order to have a 0.0014 ng/µL OXA-48 final concentration) were preincubated for a time period of 60 min with the selected compounds and 90 min with tazobactam. The preincubation time was identified in the time-dependent inhibition experiment as suitable to detect a shift of the IC50 value. At the end of preincubation, the mix was diluted 1:5 times to restore the standard assay conditions as described for time-dependent inhibition. The reactions were started by transferring of 15 μL of enzyme-compounds or control diluted from preincubation plate into assay plate and adding 5 μL 4X nitrocefin substrate (25 µM final concentration) after 60 or 90 min. In parallel, a control experiment was assembled under the same conditions, but omitting the preincubation time between compounds and OXA-48.

The following criteria were applied to assign the mechanism of action of the compounds:

a. If the AC50 value observed in the standard assay conditions was substantially unaffected by the preincubation with the 5X samples, the corresponding compound was classified as putative reversible inhibitor.

b. If the AC50 value was significantly decreased by the preincubation with the 5X samples, the corresponding compound was classified as putative non-reversible inhibitor.

#### 3.8.3. Determination of the Apparent K_m_ and V_max_ of Nitrocefin in the Presence of AC_30_, AC_50_ or AC_70_ of the Selected Inhibitors

In this study, the characterization of the type of reversible inhibition was performed by the determination of the apparent *K*_m_ and V_max_ of nitrocefin in the presence of three concentrations of the selected inhibitors.

Stock AC_30_, AC_50_, and AC_70_ solutions for selected compounds were prepared at 200X in 100% DMSO. 2X compounds were prepared by diluting stock AC_30_, AC_50_ and AC_70_ solutions 1:100 in 1X reaction buffer.

20 μL of 2X compounds with 1% DMSO; 10 μL 4X enzyme (0.0014 ng/µL OXA-48 final concentration) were dispensed into preincubation plate and kept for 15′ at rt. The reactions were started by transferring 15 μL of enzyme-compounds or enzyme-control from preincubation plate into assay plate and adding 5 μL 4X nitrocefin substrate solutions (50–25–12.5–6.25–3.125–1.56–0.78–0 μM final concentrations) ranging from limiting concentrations (typically ≤ 3-fold *K*_m_ value) up to saturating concentrations (typically ≥ 5-fold *K*_m_ value). Each compound AC_n_ was tested in duplicate against each dose of nitrocefin. Compound activity was quantified on the fluorescence signal measured at 520 nm (kinetic measurement for 60 min of reaction). The initial velocity (V_0_) of the reaction was computed in the first 300′’ of measurements as robust slope. The slopes computed by Genedata Screener 13.0.5. were copied in GraphPad Prism (version 7.00, GraphPad Software Inc.) for the Michaelis-Menten analysis and the Lineweaver-Burk analysis. The average of slope replicates (*n* = 2) was transformed into double-reciprocal and linear regression analysis was applied.

The following criteria were applied to assign the type of reversible inhibition of the compounds:

a. When the V_max_ value was independent from inhibitor concentration and the *K*_m_ value of the substrate was directly proportional to the inhibitor concentration, the corresponding compound was assigned as competitive inhibitor.

b. When the V_max_ value was inversely proportional to the inhibitor concentration and the *K*_m_ value of the substrate was substantially independent from inhibitor concentration, the corresponding compound was assigned as non-competitive inhibitor.

c. When the V_max_ value and the *K*_m_ value of the substrate were both inversely proportional to the inhibitor concentration, the corresponding compound was assigned as uncompetitive inhibitor.

#### 3.8.4. Determination of the K_i_ and a Values

V_0_ data obtained for the determination of apparent *K*_m_ and V_max_ of nitrocefin in the presence of three concentrations of the selected inhibitors, were used for the computation of Ki and a values using in GraphPad Prism (version 8.00, GraphPad Software Inc.) the mixed model inhibition/nonlinear regression curve fit method.

### 3.9. MICs Assays

All BLIs were dissolved in 100% DMSO and they were tested at a fixed concentration of 32 µg/mL and 1% of DMSO final concentration. Avibactam was used as reference β-lactamase inhibitor. All antimicrobials were prepared in line with CLSI susceptibility testing standards [66]. AMP (USP # 1033000; batch no. K1M493) was dissolved at 5.12 mg/mL, IMP (USP # 1337809; batch no. R038R0) and avibactam (Biochem # NXL 104; batch no. 20180726) were dissolved at 1.28 mg/mL in the respective solvents recommended by CLSI (2). Solutions at 4X the final concentrations of the test range were made in Cation adjusted Mueller-Hinton broth (CA-MHB, BD # 212322; batch no. 6117994; Expiry date: 29.02.2020) by serial dilutions, and then diluted 2-fold with CA-MHB (for antimicrobials alone), or with the BLIs/avibactam (for combinations of new/reference inhibitors and antimicrobials). Moreover, all BLIs were tested alone at a fixed concentration of 32 μg/mL. The final concentration of DMSO in the test tubes was 1% for each different combination.

50 μL of each well were then transferred from the masterblock into 96-well plates to whom were then added 50 μL of the final inoculum. MIC tests were performed by broth microdilution in line with CLSI susceptibility testing standards [65,66]. Bacterial inocula were prepared at ca. 1 × 106 CFU/mL by diluting 100-fold a 0.5 McFarland suspension in CA-MHB with TES (TREK Diagnostic Systems, Product code: T3462; Batch: 356347; Expiry date: 12-Jun-2019). 50 μL of antibacterial solutions at 2 × the final concentrations were diluted 2-fold with 50 μL of inoculum to give a final inoculum of ca. 5 × 105 CFU/mL and desired test concentrations of antibacterial agents. Test plates were incubated according to CLSI guidelines and read visually [65,66]. MIC values corresponded to the first well with no visible growth.

### 3.10. Aqueous Solubility Assay (PBS, pH 7.4)

Compound was dissolved in DMSO as stock solution and then added to isotonic buffer (pH 7.4) at a concentration of 200 μM (2% DMSO final concentration). After a 24 h incubation under shaking at room temperature, dissolved compound is measured by a chromatographic procedure with photodiode array detection. Metoprolol tartrate, rifampicin, ketoconazole, phenytoin, haloperidol, simvastatin, diethylstilbestrol, and tamoxifen were used as reference compounds for different solubility degrees.

### 3.11. Cytotoxicity Assay

Compound **ID2** was tested in cell viability assays with HepG2 cells. The viability of cultured cells was evaluated using alamarBlue staining as described by Nociari et al. [69]. Concentration–response curves were prepared for each compound by testing eight concentrations in triplicate with three-fold dilutions starting from 100 µM to 0.030 µM. After incubation for 48 h at 37 °C, the percentage of control activity was calculated by comparing the fluorescence reading in the presence of the test compound to that in the absence of a test compound (control). IC_50_ values were determined by non-linear regression analysis of the concentration–response curves. These parameters were obtained by Hill equation curve fitting. Chlorpromazine was used as a reference compound.

## 4. Conclusions

Nowadays, the combination of susceptible β-lactams with BLIs, which protect them from degradation and restore their antibacterial activity, serves as the major strategy for overcoming antibiotic resistance. Nevertheless, the emergence of carbapenemases like OXA-48 that significantly contribute to the global burden of antibiotic resistance, has highlighted the limited utility of current clinical treatments and the need for novel specific BLIs. In this respect, we screened a proprietary compound collection in HTS against *K. pneumoniae* OXA-48 and identified several compounds belonging to 20 different chemotypes able to inhibit the enzyme at micromolar concentration. The favorable hit rate of the screening campaign (0.5% considering 176 hits out of 36426 tested compounds) and the good quality of identified hits (77% of final hits with LE > 0.2) proved the potential of the tested library and the success of HTS triage strategy in identifying OXA-48 inhibitors.

We then prioritized the 4-ideneamino-4*H*-1,2,4-triazole (SC_2) and pyrazolo[3,4-b]pyridine (SC_7) derivatives for further investigation and demonstrated by biochemical and X-ray studies a reversible and competitive binding mode within OXA-48 active site for both series. Such an MoA might represent an alternative to the covalent inhibition of clinically available BLIs.

We analyzed the OXA-48 inhibitory data of both commercial and synthesized analogs of SC_2 and SC_7 families and built up a robust SAR around the two series, which highlighted the key role of negatively ionizable chemical features, such as carboxylate and thiolate groups, for target inhibition.

The most promising derivative of SC_7 (**ID2**, AC_50_ = 0.99 μM), tested in combination with AMP and IMP in β-lactam resistant bacteria, lowered the MIC of IMP of two-fold in *E. coli* ATCC BAA-2523. Despite this modest activity, we envisage that **ID2** could be amenable to structural changes to allow for optimization of the whole cell potency of later stage compounds.

**ID2** also showed favorable properties in a preliminary in vitro ADMET assessment. Overall, these findings suggest SC_7 as a promising starting point for further hit optimization programs toward novel and effective non-β-lactam BLIs with an innovative MoA.

## Figures and Tables

**Table 1:** Analysis of the hit rate and AC_50_ distribution across scaffolds.

Scaffold	Confirmed Hit	Tot Tested Cmpd ^a^	Hit Rate ^b^	AC_50_ (µM) ^c^					
				Most Active	≤5	5–10	10–30	30–60	>60
SC_2	38	287	13.2%	0.72	4		15	11	8
SC_4	5	76	6.6%	45.80				1	4
SC_5	2	136	1.5%	69.40					2
SC_7	2	37	5.4%	0.99	1				1
SC_9	8	206	3.9%	31.90				4	4
SC_11	6	174	3.4%	35.10				1	5
SC_12	11	24	45.8%	37.10				5	6
SC_15	22	133	16.5%	29.30			1	11	10
SC_16	3	41	7.3%	62.40					3
SC_17	24	77	31.2%	13.40			6	5	13
SC_18	10	45	22.2%	9.15		3	3	1	3
SC_19	4	76	5.3%	26.60			1	1	2
SC_20	4	14	28.6%	4.14	1	1		1	1
SC_21	2	156	1.3%	3.21	1				1
SC_22	3	401	0.7%	1.81	2		1		
SC_23	18	233	7.7%	20.60			2	4	12
SC_24	1	13	7.7%	373.85					1
SC_26	1	33	3.0%	651.84					1
SC_30	8	105	7.6%	79.50					8
SC_31	4	42	9.5%	21.40			1		3

^a^ This number refers to all compounds tested in the primary screening, HC, HE and activity determination steps. ^b^ The hit rate was calculated as ratio between the confirmed hits and the total number of tested compounds per chemical class. ^c^ Five AC_50_ ranges were considered, each range was considered as half-closed interval of activity percent in which the largest endpoint was included.

**Table 2:** Dilution after preincubation with **ID1**, **ID2**, and **tazobactam**.

Compound	Structure	Time [Min] ^a^	OXA-48 AC_50_ (µM)	
			Standard Preincubation ^b^	High Dose Preincubation ^c^
ID1	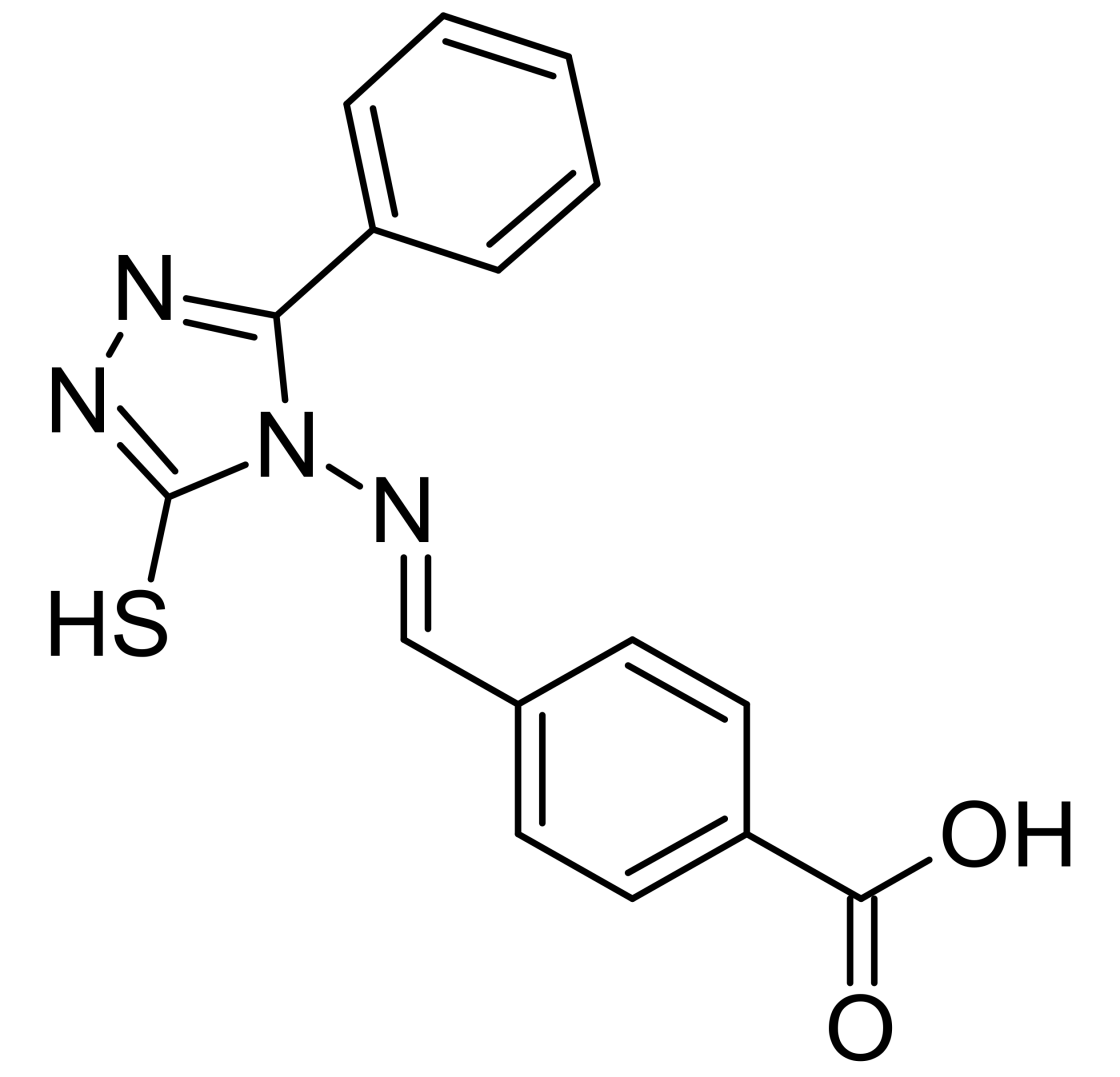	0	1.73	0.63
		60	1.93	1.09
ID2	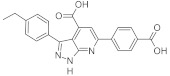	0	2.31	1.01
		60	2.44	1.45
tazobactam		0	0.82	0.79
		90	0.26	0.097

^a^ Dose–response curves of the selected compounds were preincubated with OXA-48 enzyme for 0 and 60 min. ^b^ A control experiment was assembled in the standard concentration condition. ^c^ The experiment was conducted using 10X-concentrated doses of the compounds and 20X concentrated OXA-48 in the preincubation step. At the end of the preincubation time, the samples were diluted 1:5 to restore the standard assay conditions and the reactions were started by adding the nitrocefin substrate.

**Table 3:** Derived K_m_ and V_max_ Average Values +/− Standard Error (SE) of nitrocefin and OXA-48 Enzyme respectively Varying the Concentration of OXA-48 Inhibitors **ID1** and **ID2**.

Conc (µM)	ID1		ID2	
	V_max_ ± SE ^a^	K_m_ ± SE ^a^	V_max_ ± SE ^a^	K_m_ ± SE ^a^
0	116.8 ± 4.62	9.6 ± 1.06	116.8 ± 4.62	9.6 ± 1.06
0.126	113.4 ± 8.72	12.7 ± 2.51	110.8 ± 7.80	10.7 ± 2.04
0.632	104.0 ± 7.50	17.3 ± 2.91	106.5 ± 7.88	15.0 ± 2.70
3.16	71.9 ± 12.50	29.2 ± 10.00	84.3 ± 10.91	29.2 ± 7.44

^a^ Vmax and Km values were calculated as median of triplicate and the standard error (SE) is reported.

**Table 4:** SAR Exploration at R_1_ and R_2_ positions of the 4-Ideneamino 4H-1,2,4-triazole-3-thiol Core.

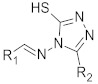						
**Compound**	**R_1_**	**R_2_**	**OXA-48 Activity% ± SE ^a^**	**OXA-48 AC_50_ (μM) ^b^**	**Lower 95% CL**	**Upper 95% CL**
ID1	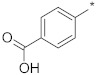	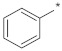	−92.19 ± 3.86	1.14	1.03	1.27
ID3	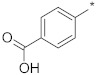	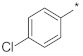	−94.10 ± 0.48	0.72	0.65	0.80
ID4	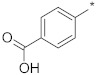	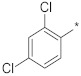	−85.80 ± 0.09	3.17	2.86	3.51
ID5	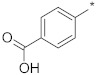	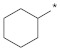	−88.29 ± 2.58	2.88	2.57	3.24
ID6	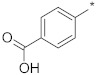	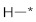	−61.30 ± 0.08	15.70	14.01	17.60
ID7	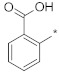	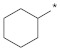	−1.24 ± 0.05	N.A. ^c^		
ID8	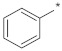	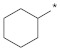	−1.78 ± 0.268	N.A. ^c^		
ID9	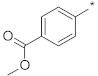	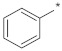	−23.84 ± 1.45	35.60	32.03	39.64
ID10	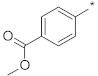	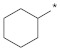	−6.64 ± 1.05	N.A. ^c^		
ID11	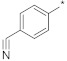	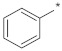	−58.40 ± 0.96	12.00	9.82	14.60
ID12	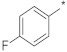	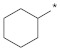	−1.33 ± 1.30	N.A. ^c^		
ID13	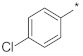	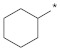	0.02 ± 1.18	N.A. ^c^		
ID14	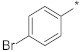	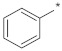	−9.43 ±1.35	N.A. ^c^		
ID15	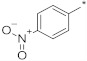	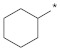	9.13 ± 5.58	N.A. ^c^		
ID16	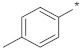	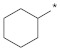	−0.68 ± 2.27	N.A. ^c^		
ID17	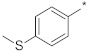	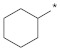	−6.76 ± 0.02	N.A. ^c^		
ID18	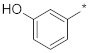	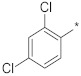	−5.55 ± 0.73	N.A. ^c^		
ID19	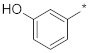	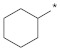	−18.85 ± 1.53	N.A. ^c^		
ID20	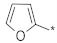	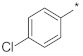	−15.40 ± 1.77	N.A. ^c^		
ID21	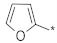	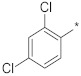	−6.90 ± 0.43	N.A. ^c^		
ID22	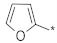	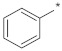	−5.12 ± 0.84	N.A. ^c^		
ID23	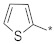	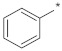	−29.46 ± 3.40	24.80	22.26	27.57
ID24	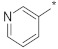	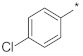	−2.71 ± 0.78	N.A. ^c^		
ID25	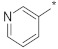	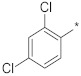	1.40 ± 1.52	N.A. ^c^		
ID26	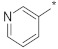	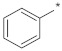	−9.29 ± 1.75	N.A. ^c^		
ID27	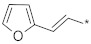	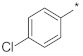	1.32 ± 1.03	N.A. ^c^		
ID28	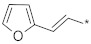	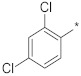	1.10 ± 1.56	N.A.^c^		
ID29	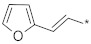	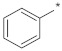	0.08 ± 0.16	N.A. ^c^		
tazobactam				0.84	0.78	0.91

^a^ Activity percent values at 20 μM were calculated as median of triplicate wells and the standard error (SE) is reported. ^b^ AC_50_ values were calculated from data points obtained as a median of triplicate wells. ^c^ N.A. = data not available.

**Table 5:** SAR exploration at R1 and R2 positions of the pyrazolo[3,4-b]pyridine core.

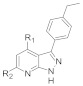						
**Compound**	**R_1_**	**R_2_**	**OXA-48 Activity% ± SE ^a^**	**OXA-48 AC_50_ (** **μ** **M) ^b^**	**Lower 95% CL**	**Upper 95% CL**
ID2	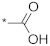	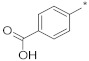	−93.92 ± 0.54	0.99	0.80	1.22
ID30	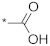	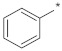	−13.17 ± 1.07	248.59	176.58	349.95
ID31	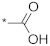	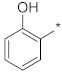	−25.86 ± 0.57	N.C. ^c^		
1	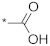	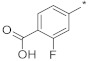	−93.15 ± 0.46	0.92	0.86	0.99
2	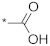	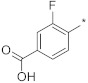	−86.38 ± 0.35	1.48	1.35	1.62
3	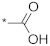	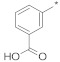	−49.91 ± 1.58	16.79	15.41	18.29
4	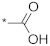	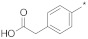	−87.36 ± 0.60	4.78	4.46	5.13
5	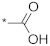	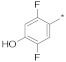	−55.90 ± 0.64	16.21	14.64	17.95
6	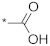	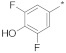	−46.92 ± 1.73	24.38	21.88	27.15
7	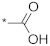	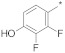	−28.26 ± 0.31	51.21	45.08	58.17
8	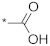	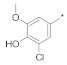	−64.34 ± 1.55	13.69	12.23	15.33
9	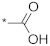	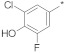	−14.72 ± 2.19	63.77	58.40	69.63
10	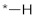	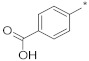	−10.57 ± 1.45	>100		
11	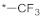	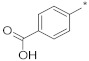	−12.66 ± 0.87	>100		
12	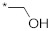	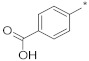	−4.62 ± 1.13	>100		
13	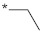	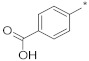	−9.28 ± 1.06	>100		
14	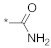	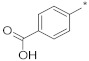	−0.74 ± 0.97	N.C. ^c^		
tazobactam				0.84	0.78	0.91

^a^ Activity percent values at 20 μM were calculated as median of triplicate wells and the standard error (SE) is reported. ^b^ AC_50_ values were calculated from data points obtained as a median of triplicate wells. ^c^ N.C. = not calculated.

**Table 6:** Antibacterial activity of AMP and IMP in combination with **ID2** and **ID3**.

Bacterial Strain	Strain Code	β-lactam	MIC β-lactam (µg/mL)	MIC (µg/mL) (β-lactam + BLI)			
				Avibactam (4 µg/mL)	Tazobactam (32 µg/mL)	ID2 (32 µg/mL)	ID3 (32 µg/mL)
*E.coli*	ATCC25922	AMP	4	2	4	4	8
		IMP	0.12	0.06	0.12	0.12	0.12
		no	-	-	-	>32	>32
*E.coli*	ATCC BAA-2523	AMP	>512	16	256	>512	>512
		IMP	2	≤0.03	0.12	0.5	1
		no	-	-	>32	>32	>32
*K.pneumoniae*	ATCC BAA-2524	AMP	>512	128	>512	>512	>512
		IMP	4	0.5	1	2	2
		no	-	-	>32	>32	>32

## Data Availability

The data presented in this study are available in the main text and Supplementary Materials.

## Supplementary Materials

The following are available online at https://www.mdpi.com/article/10.3390/ph14070612/s1, Figure S1: (a) Bar chart showing the distribution of the hits across the AC_50_ ranges. (b) Pie chart showing the frequency of the confirmed OXA-48 inhibitors across the chemical classes; Figure S2: Detailed binding mode of cocrystal structure of **ID3** in complex with OXA48 enzyme from different perspectives; Figure S3: Detailed binding mode of cocrystal structure of **ID2** in complex with OXA-48 enzyme from different perspectives; Table S1: 2D structures of the thirty-eight compounds belonging to SC_2 group showing measurable OXA-48 AC_50_ (μM); Table S2: 2D structures of the thirty-seven compounds belonging to SC_7 group tested in the HTS; Table S3: Statistics of crystallographic data and refinement for crystals of OXA-48 in complex with **ID2** and **ID3,** 1H and 13C NMR spectra of final ligands **1**–**14**, HPLC and LC/MS analysis of final ligands **1**–**14**. (PDF).

## Abbreviations

AMP: ampicillin; AUC, area under the curve; BLI, β-lactamase inhibitor; CHDLs, Carbapenem-Hydrolyzing class D β-Lactamases; CLSI, Clinical and Laboratory Standards Institute; CSR, cyclic systems retrieval; *E. coli*, *Escherichia coli*; EI, enzyme-inhibitor; HAC, heavy atom count; HC, hit confirmation; HE, hit expansion; HTS, high throughput screening; IMP, imipenem; IP, intellectual property; *K. pneumoniae, Klebsiella pneumoniae*; SAR, structure-activity relationships; SE, standard error; WGS, whole genome sequencing.
