# Supplementary material for: Discovery of Novel Chemical Series of OXA-48 β-Lactamase Inhibitors by High-Throughput Screening
Source: Pharmaceuticals (Basel). 2021 Jun 25;14(7):612. doi: 10.3390/ph14070612 (PMC8308845; doi:10.3390/ph14070612)
Supplement: Supplementary file 1 [file pharmaceuticals-14-00612-s001.zip › pharmaceuticals-1244286-supplementary.pdf]

Discovery of Novel Chemical Series of OXA-48 β-Lactamase Inhibitors by High-Throughput Screening

Barbara Garofalo [1], Federica Prati [1], Rosa Buonfiglio [1], Isabella Coletta [1], Noemi D'Atanasio [1], Angela Molteni [2], Daniele Carettoni [2], Valeria Wanke [2], Giorgio Pochetti [3], Roberta Montanari [3], Davide Capelli [3], Claudio Milanese [1], Francesco Paolo Di Giorgio [1], and Rosella Ombrato [1,*]

[1] Angelini Pharma S.p.A., Global R&D External Innovation, Viale Amelia 70, 00181 Rome, Italy; barbara.garofalo@angelinipharma.com (B.G.); federica.prati@angelinipharma.com (F.P.); rosa.buonfiglio@angelinipharma.com (R.B.); isabella.coletta@angelinipharma.com (I.C.); noemi.datanasio@angelinipharma.com (N.D.); claudio.milanese@angelinipharma.com (C.M.); francescopaolo.digiorgio@angelinipharma.com (F.P.D.G.)

[2] Axxam SpA Via Meucci 3, Bresso, 20091 Milan, Italy; Angela.Molteni.AM@axxam.com (A.M.); Daniele.Carettoni.DC@axxam.com (D.C.); Valeria.Wanke.VW@axxam.com (V.W.)

[3] Consiglio Nazionale delle Ricerche—Istituto di Cristallografia, Via Salaria—km 29.300, Monterotondo, 00015 Rome, Italy; giorgio.pochetti@ic.cnr.it (G.P.); roberta.montanari@ic.cnr.it (R.M.);
davide.capelli@ic.cnr.it (D.C.)

* Correspondence: rosella.ombrato@angelinipharma.com

This section provides information on additional tables, schemes and figures mentioned in the main text.

Table of contents

Figure S1. a) Bar chart showing the distribution of the hits across the AC_{50} ranges. b) Pie chart showing the frequency of the confirmed OXA-48 inhibitors across the chemical classes. S2
Figure S2. Detailed binding mode of cocrystal structure of **ID3** in complex with OXA48 enzyme from different perspectives. S2
Figure S3. Detailed binding mode of cocrystal structure of **ID2** in complex with OXA48 enzyme from different perspectives. S2

Table S1. 2D structures of the thirty-eight compounds belonging to SC_2 group showing measurable OXA-48 AC_{50} (μM) S3-S8
Table S2. 2D structures of the thirty-seven compounds belonging to SC_7 group tested in the HTS S9-S15
Table S3. Statistics of crystallographic data and refinement for crystals of OXA-48 in complex with **ID2** and **ID3** S16

1H and 13C NMR spectra of final ligands **1-14** S17-S30

HPLC and LC/MS analysis of final ligands **1-14** S31-S44

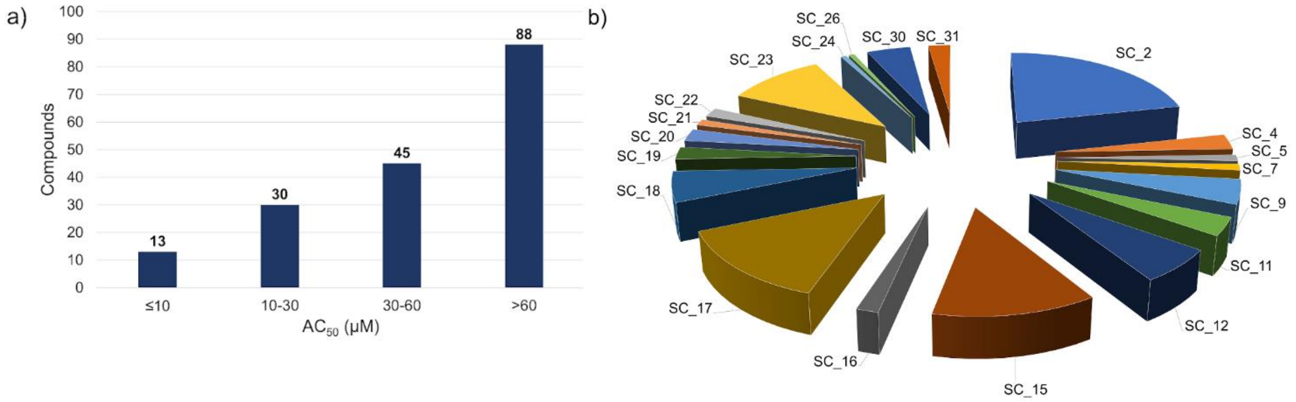

Figure S1. a) Bar chart showing the distribution of the hits across the AC_{50} ranges. b) Pie chart showing the frequency of the confirmed OXA-48 inhibitors across the chemical classes.

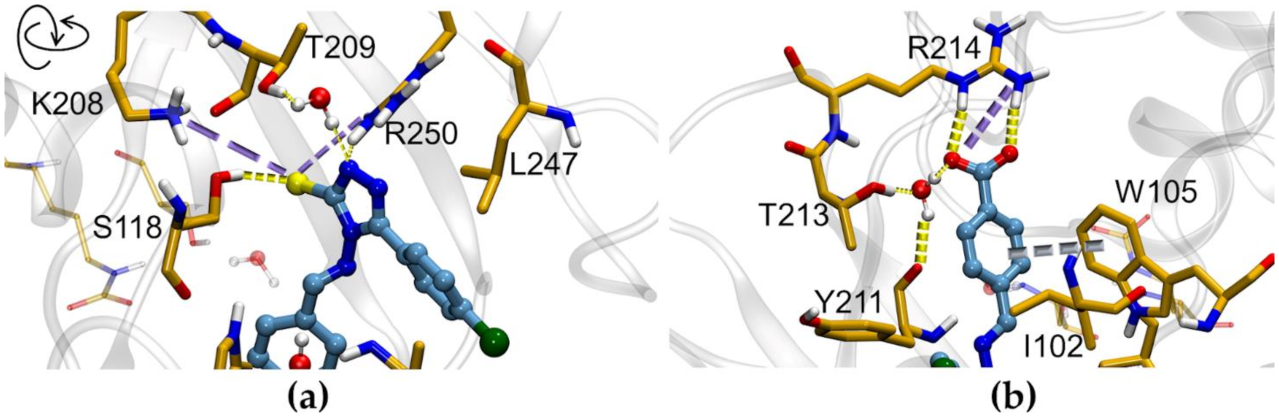

Figure S2. Detailed binding mode of cocrystal structure of **ID3** in complex with OXA48 enzyme from different perspectives. a) thiolate side (rotation with respect to Figure 13b); b) benzoic acid side. Hydrogen bonds, electrostatic and π-π interactions are represented as yellow, purple and grey dashed lines. For the sake of clarity, some portions of the protein have been omitted.

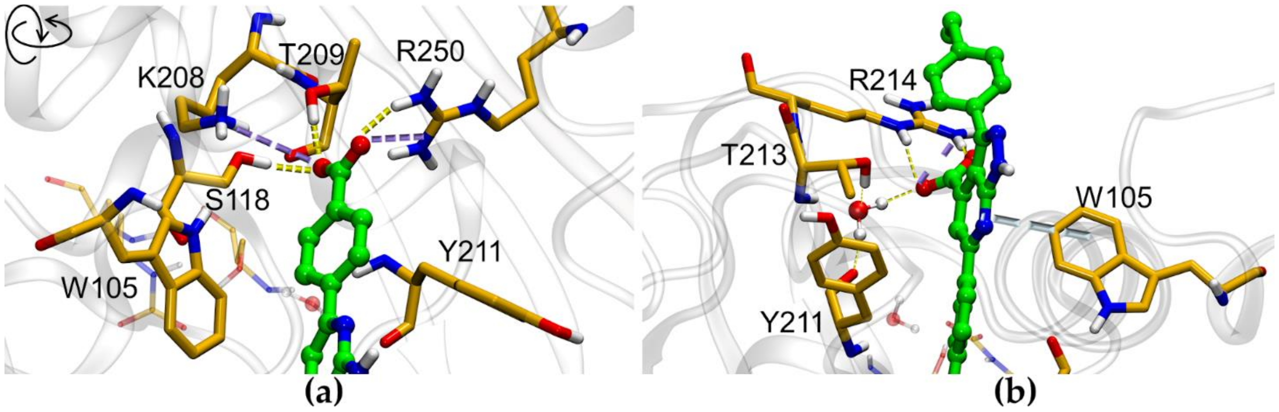

Figure S3. Detailed binding mode of cocrystal structure of **ID2** in complex with OXA48 enzyme from different perspectives. a) benzoic acid side (rotation with respect to Figure 14); b) 4-COOH group side. Hydrogen bonds, electrostatic and π-π interactions are represented as yellow, purple and grey dashed lines. For the sake of clarity, some portions of the protein have been omitted.

Table S1. 2D structures of the thirty-eight compounds belonging to SC_2 group showing measurable OXA-48 AC_{50} (μM)

Structure	ID	OXA-48 AC_{50} (μM)[a]	Lower 95% CL	Upper 95% CL	OXA-48 Activity%±SE[b]
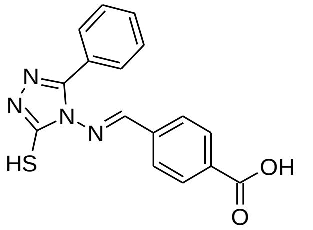	**ID1**	1.14	1.033	1.267	-92.19±3.86
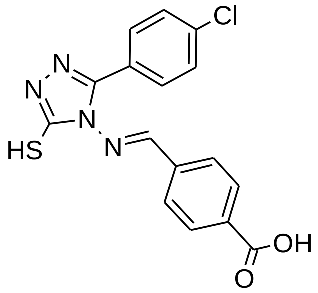	**ID3**	0.723	0.653	0.801	-94.1±0.48
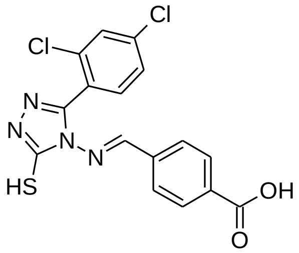	**ID4**	3.17	2.861	3.510	-85.8±0.09
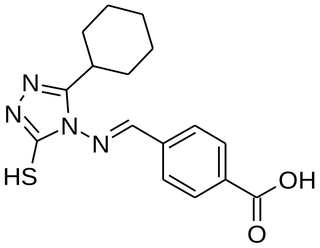	**ID5**	2.88	2.571	3.235	-88.29±2.58
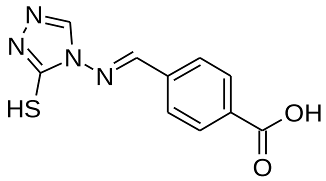	**ID6**	15.7	14.005	17.603	-61.3±0.08
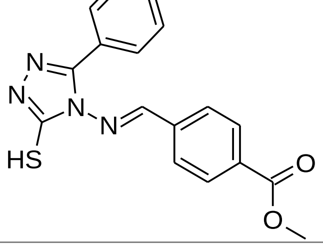	**ID9**	35.6	32.031	39.637	-23.84±1.45

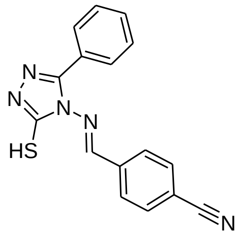	**ID11**	12	9.821	14.600	-58.4±0.96
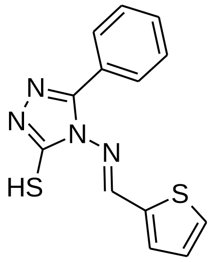	**ID23**	24.8	22.263	27.568	-29.46±3.40
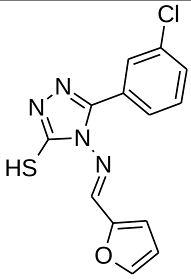	**ID32**	16.6	14.556	18.834	-47.99±2.92
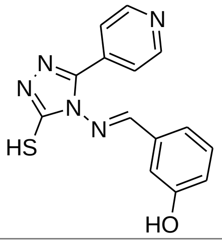	**ID33**	16.7	15.319	18.167	-62.46±2.46
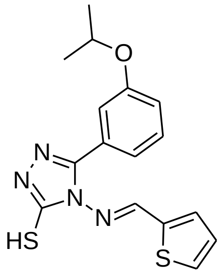	**ID34**	17.1	14.319	20.371	-42.4±1.83
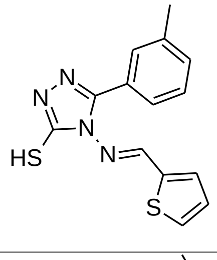	**ID35**	18	16.537	19.498	-39.58±0.79
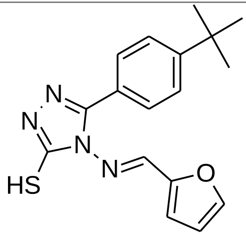	**ID36**	21.3	20.106	22.560	-28.58±1.13

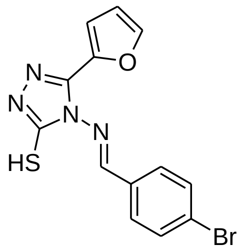	**ID37**	22.8	18.568	28.044	-53±0.95
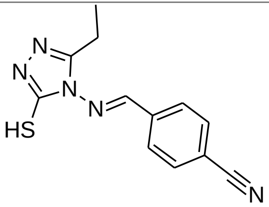	**ID38**	24	22.175	25.925	-44.71±2.01
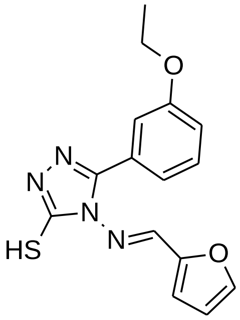	**ID39**	24.8	23.157	26.621	-32.57±2.09
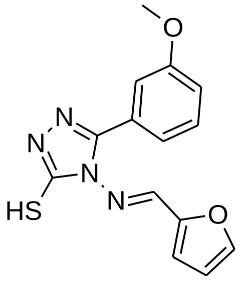	**ID40**	25.2	23.317	27.217	-40.31±2.06
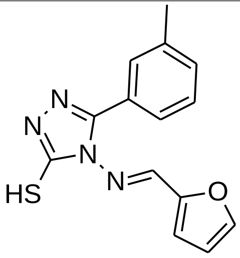	**ID41**	25.7	23.844	27.599	-49.33±0.40
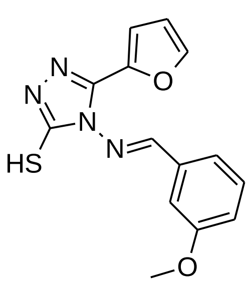	**ID42**	27.1	24.536	29.830	-44.91±1.84

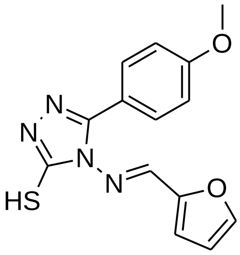	**ID43**	29.2	27.509	31.066	-31.49±0.61
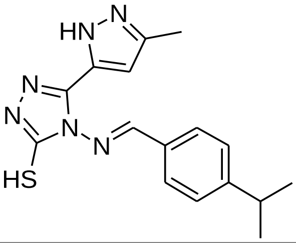	**ID44**	32.6	29.950	35.562	-24.72±2.13
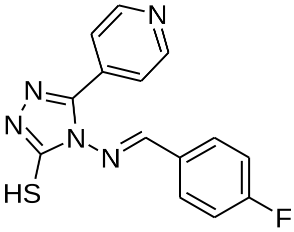	**ID45**	35.3	29.825	41.715	-42.84±1.26
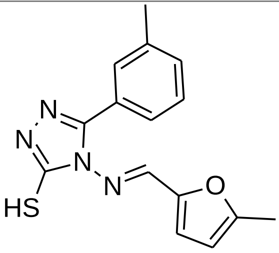	**ID46**	39	36.935	41.132	-22.94±1.81
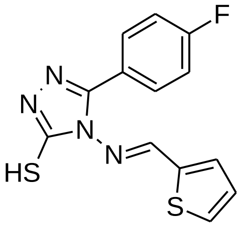	**ID47**	41.4	32.830	52.201	-30.23±1.23
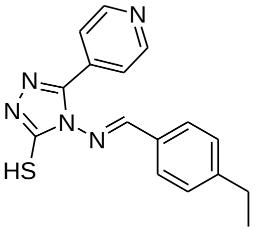	**ID48**	43.3	40.817	45.964	-21.52±2.12
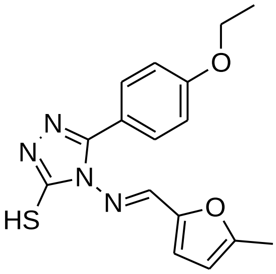	**ID49**	44.1	37.385	52.077	-40.47±1.99

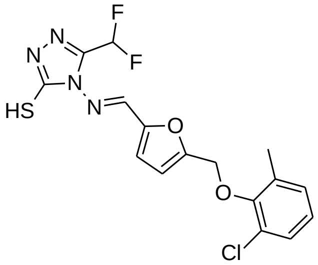	**ID50**	45.2	41.727	48.970	-23.88±0.67
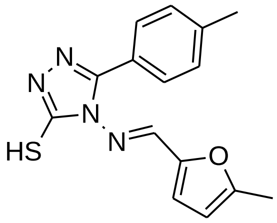	**ID51**	47.7	44.752	50.856	-20.12±0.38
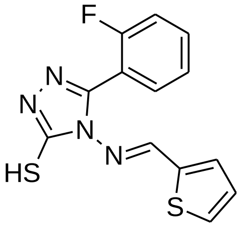	**ID52**	49.5	44.878	54.659	-22.2±0.09
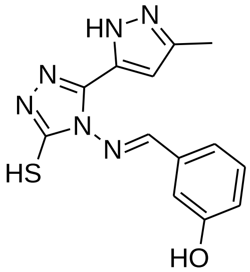	**ID53**	50.5	45.531	55.978	-20.8±2.70
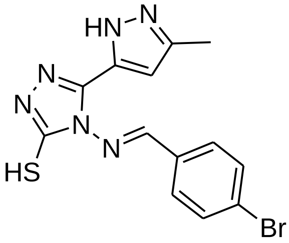	**ID54**	61.1	55.378	67.443	-24.39±1.23
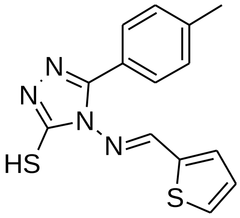	**ID55**	64.2	55.954	73.696	-26.31±0.64

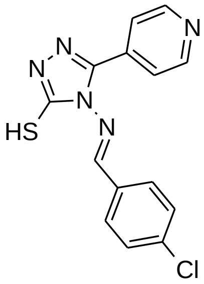	**ID56**	66	61.749	70.617	-28.62±0.26
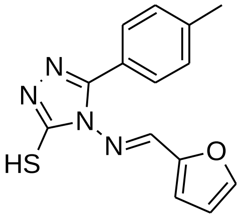	**ID57**	67.1	62.548	71.956	-26.07±0.13
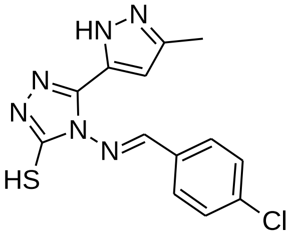	**ID58**	76.9	65.211	90.604	-27.29±0.35
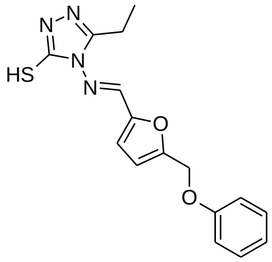	**ID59**	84	44.511	158.498	-24.82±0.10
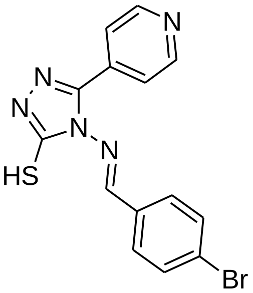	**ID60**	90	78.204	103.519	-27.4±0.52
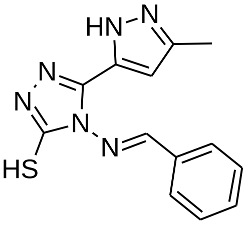	**ID61**	126.81	110.409	145.643	-17.13±2.75

[a]AC_{50} values were calculated from data points obtained as median of triplicate wells. [b]Activity percent values at 20 μM were calculated as median of triplicate wells and the standard error (SE) is reported.

Table S2. 2D structures of the thirty-seven compounds belonging to SC_7 group tested in the HTS

	ID	OXA-48 AC_{50} (μM)[a]	Lower 95% CL	Upper 95% CL	OXA-48 Activity%±SE[b]
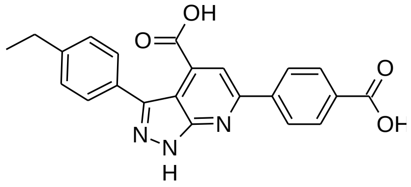	**ID2**	0.99	0.80	1.22	-93.92±0.54
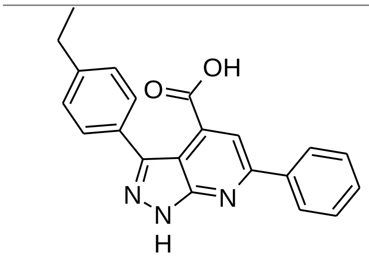	**ID30**	248.59	176.58	349.95	-13.17±1.07
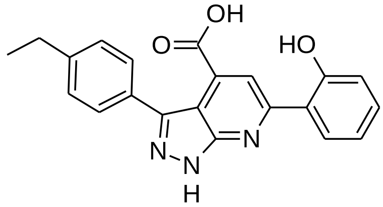	**ID31**				-25.86±0.57
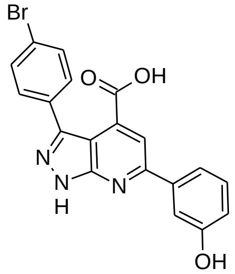	**ID62**				-16.36±0.70
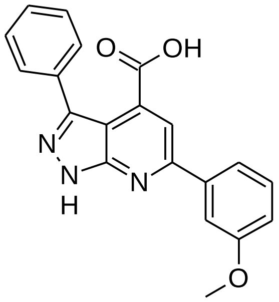	**ID63**				-12.8±0.75
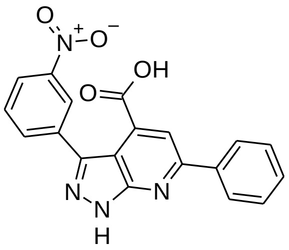	**ID64**				-12.16±1.44

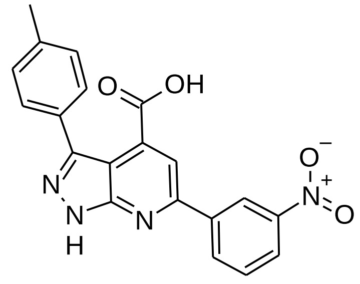	**ID65**	-10.6±0.52
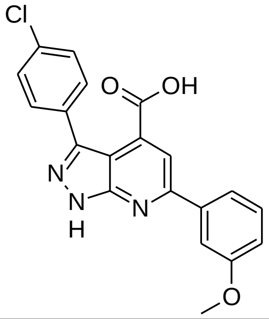	**ID66**	-10.34±0.88
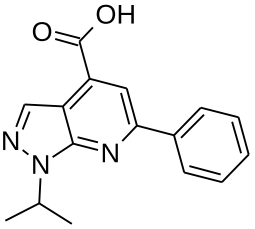	**ID67**	-9.89±1.75
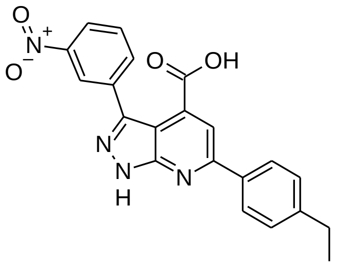	**ID68**	-9.1±0.90
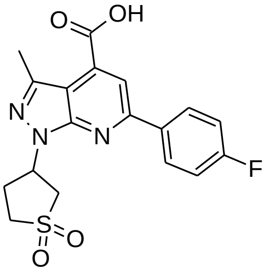	**ID69**	-8.72±0.82
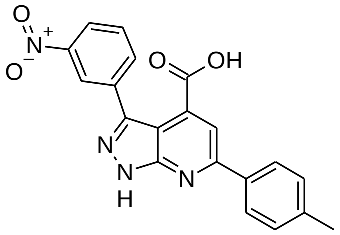	**ID70**	-8.16±0.64

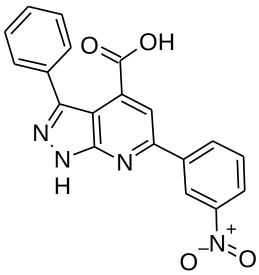	**ID71**	-7.7±0.67
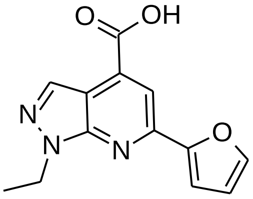	**ID72**	-5.66±3.92
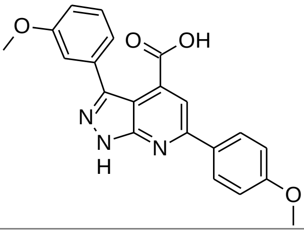	**ID73**	-5.39±0.010
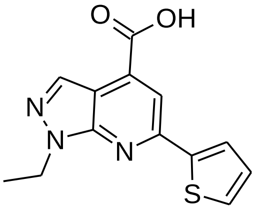	**ID74**	-5.25±0.76
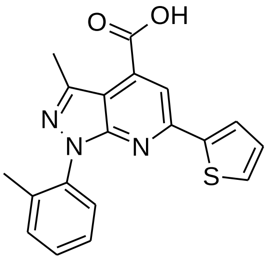	**ID75**	-4.62±2.42
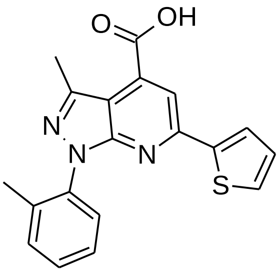	**ID76**	-4.41±0.70

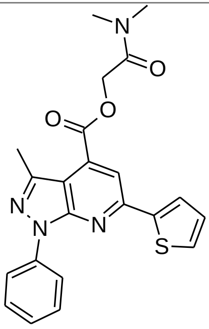	**ID77**	-4.4±0.05
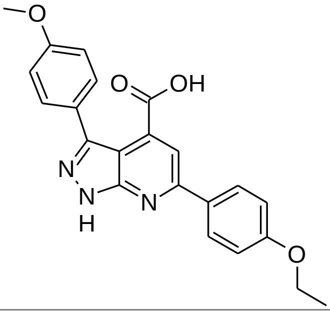	**ID78**	-3.72±1.43
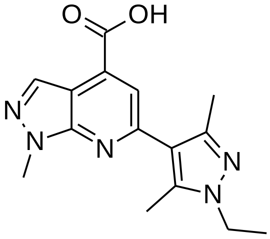	**ID79**	-3.64±0.49
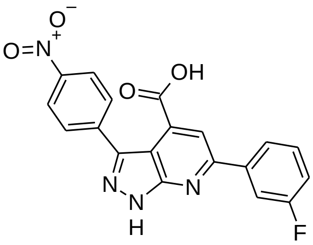	**ID80**	-3.48±0.69
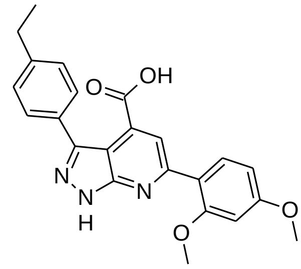	**ID81**	-3.46±3.98
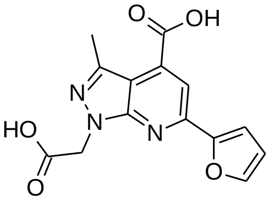	**ID82**	-3.2±0.27

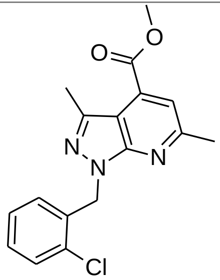	**ID83**	-2.87±1.47
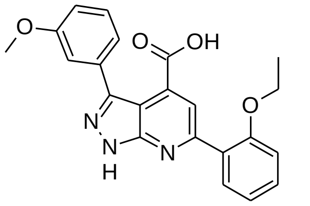	**ID84**	-2.72±0.11
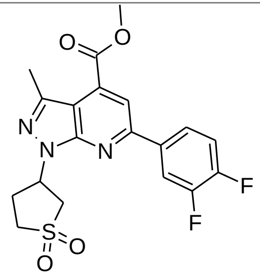	**ID85**	-2.61±0.99
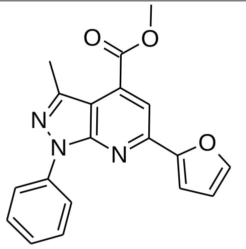	**ID86**	-2.51±1.74
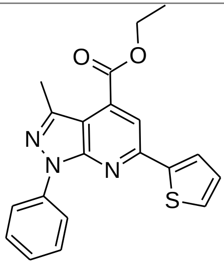	**ID87**	-2.27±0.81
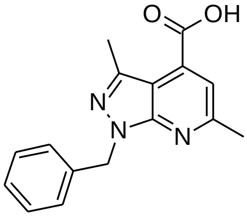	**ID88**	-1.6±0.89

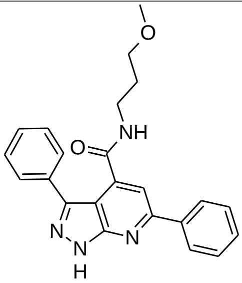	**ID89**	-1.44±2.67
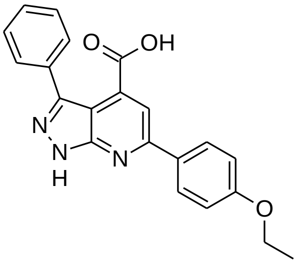	**ID90**	-1.03±0.97
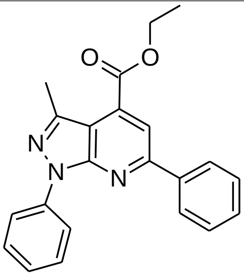	**ID91**	-0.543±0.39
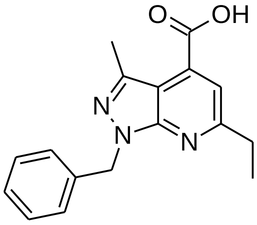	**ID92**	-0.41±1.14
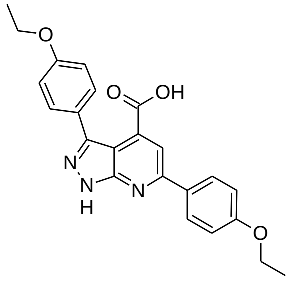	**ID93**	0.07±0.01565712
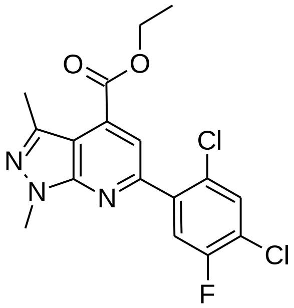	**ID94**	0.39±0.14

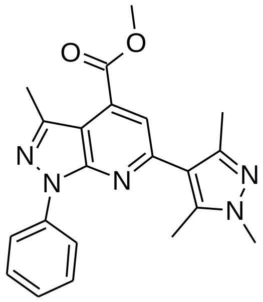	**ID95**	1.19±1.74
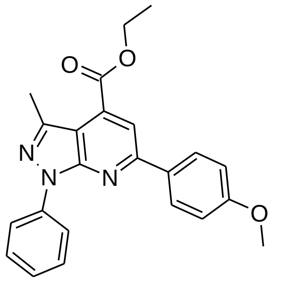	**ID96**	3.62±0.001

[a]AC50 values were calculated from data points obtained as median of triplicate wells. [b]Activity percent values at 20 μM were calculated as median of triplicate wells and the standard error (SE) is reported.

Table S3. Statistics of crystallographic data and refinement for crystals of OXA-48 in complex with **ID2** and **ID3**

	**ID2**	**ID3**
**Data collection**		
**space group**	$P2_12_12_1$	$P2_12_12_1$
**cell dimension *a*, *b*, *c* [Å]**	71.41, 72.63, 125.66	72.47, 73.68, 124.79
**wavelenght [Å]**	1.072	1.072
**resolution range [Å]**	62.88 - 2.05	51.67 - 1.65
**last shell [Å]**	2.05 - 2.11	1.65 - 1.68
R_{merge} **[%]**	8.6 (76.2) [a]	6.8 (32.8) [a]
**unique reflections**	40846	80899
**mean (*I*)/σ(*I*)**	8.2 (1.8) [a]	11.5 (4.1) [a]
**completeness**	98.2 (97.7) [a]	99.8 (99.7) [a]
**No. of molecules in asymmetric unit**	2	2
**Refinement**		
**resolution range [Å]**	62.88 - 2.05	51.67 - 1.65
R_{work} **[%]**	20.6	17.6
R_{free} **[%]**	24.5	20.7
**Bond lenghts r.m.s.d. [Å]**	0.008	0.009
**Bond angles r.m.s.d. [deg]**	1.147	1.156
**PDB code**	7AUX	7AW5

[a] The values in parenthesis refer to the outer shell.

1H and 13C NMR spectra of final ligands **1**-**14**

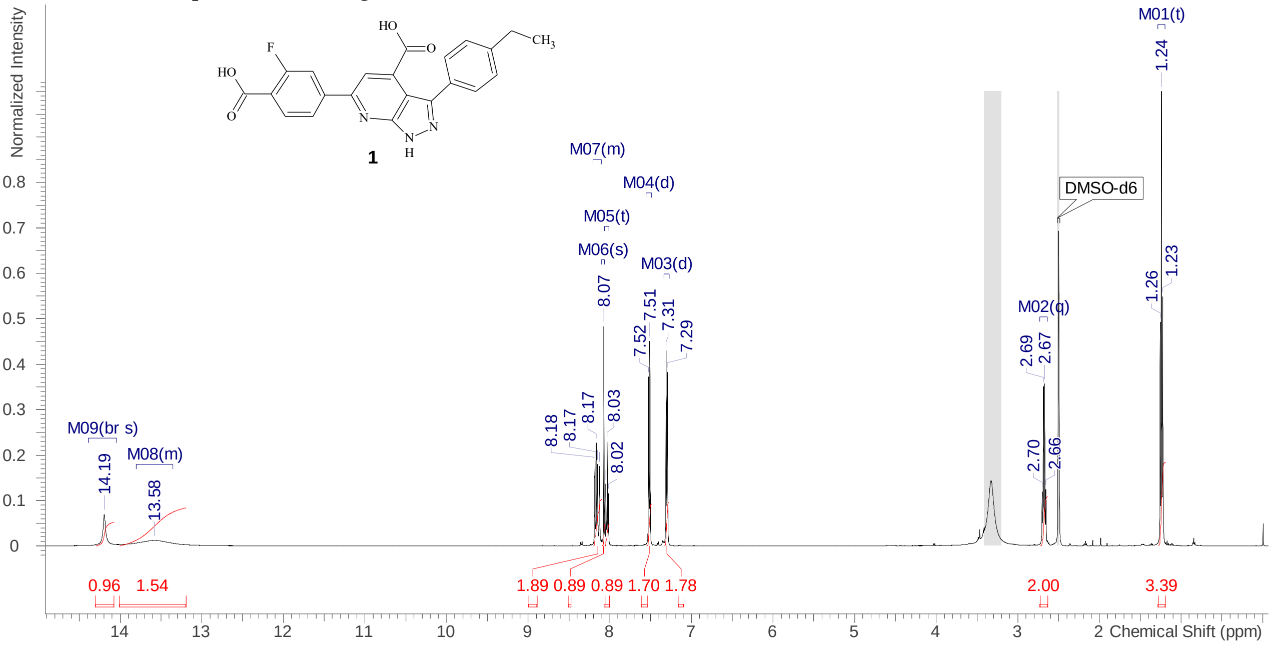

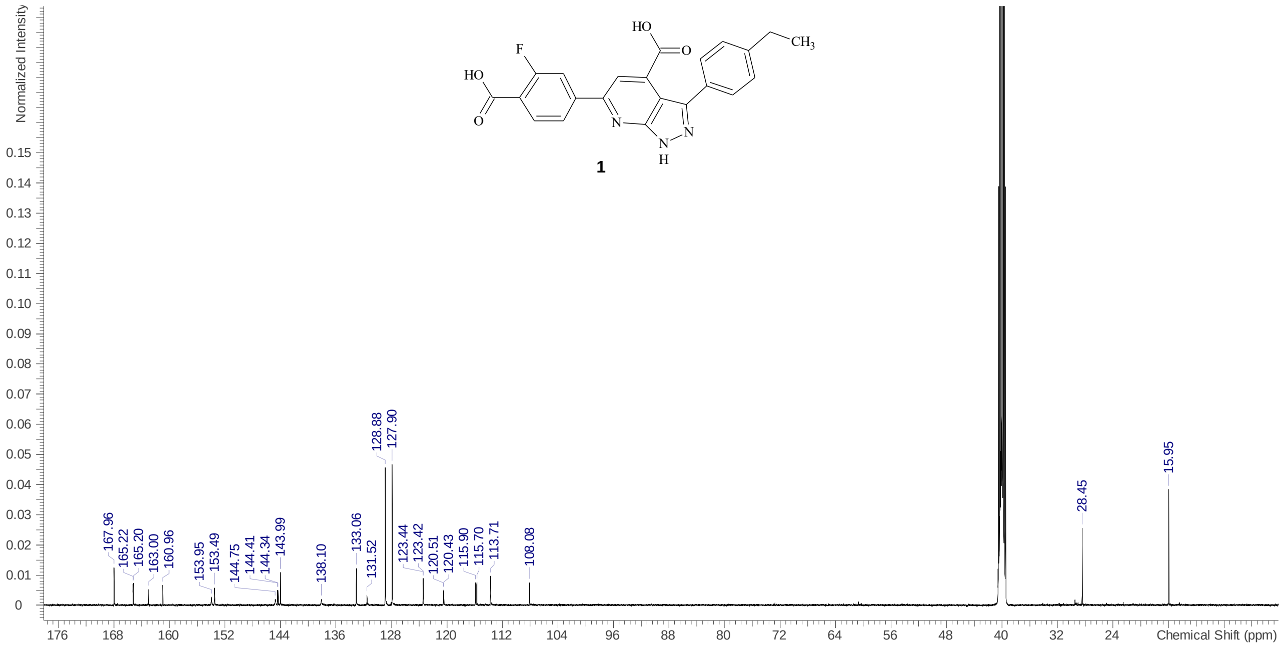

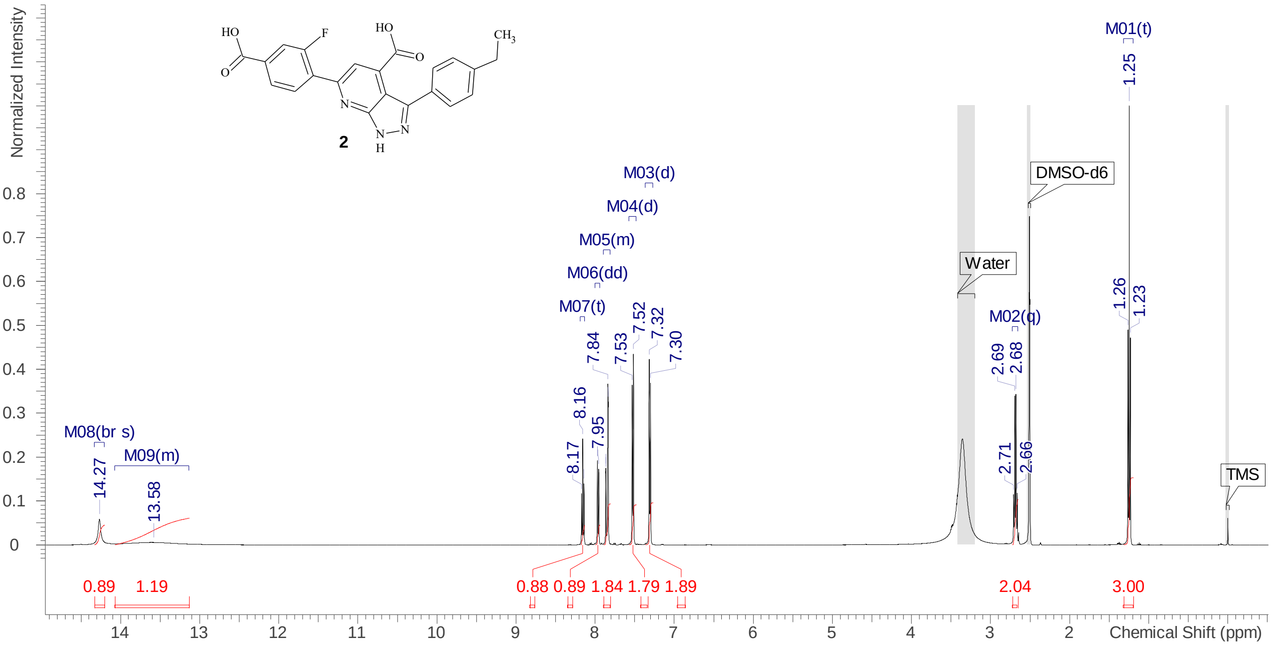
Normalized Intensity
Chemical Shift (ppm)
M01(t)
M02(q)
M03(d)
M04(d)
M05(m)
M06(dd)
M07(t)
M08(br s)
M09(m)
Water
DMSO-d6
TMS
14.27
13.58
8.17
8.16
7.95
7.84
7.53
7.52
7.32
7.30
2.71
2.69
2.68
2.66
1.26
1.25
1.23
0.89 1.19 0.88 0.89 1.84 1.79 1.89 2.04 3.00
2

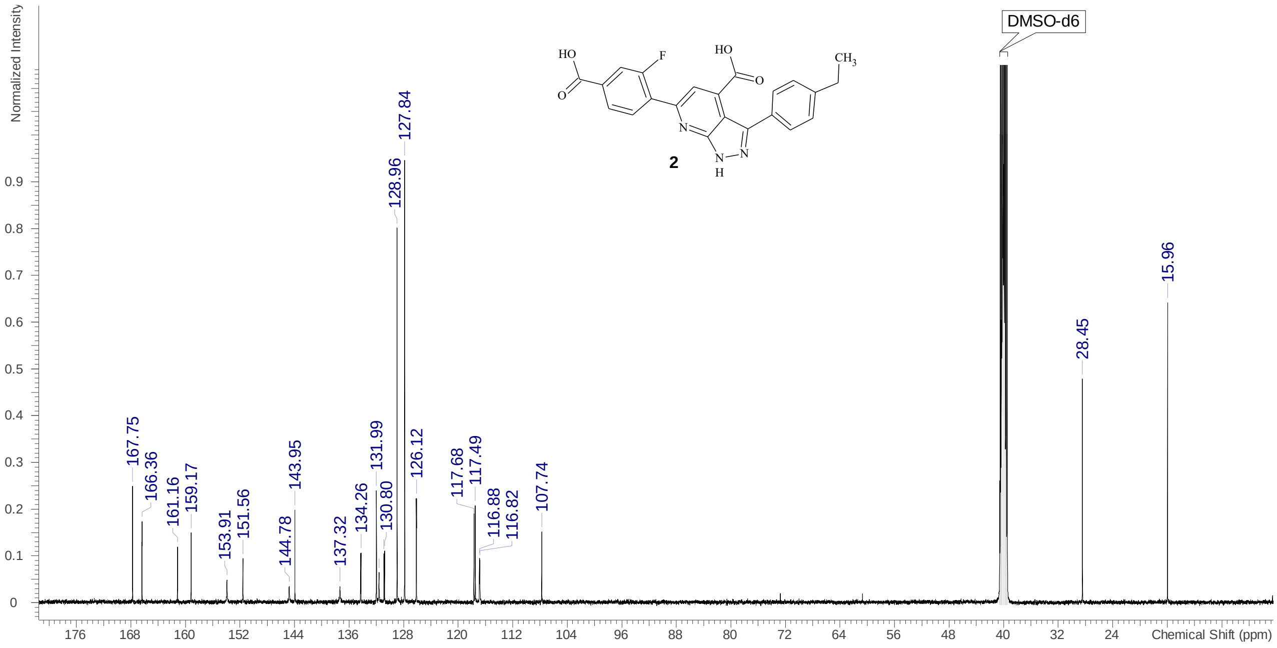
Normalized Intensity
Chemical Shift (ppm)
DMSO-d6
167.75
166.36
161.16
159.17
153.91
151.56
144.78
143.95
137.32
134.26
131.99
130.80
128.96
127.84
126.12
117.68
117.49
116.88
116.82
107.74
28.45
15.96
2

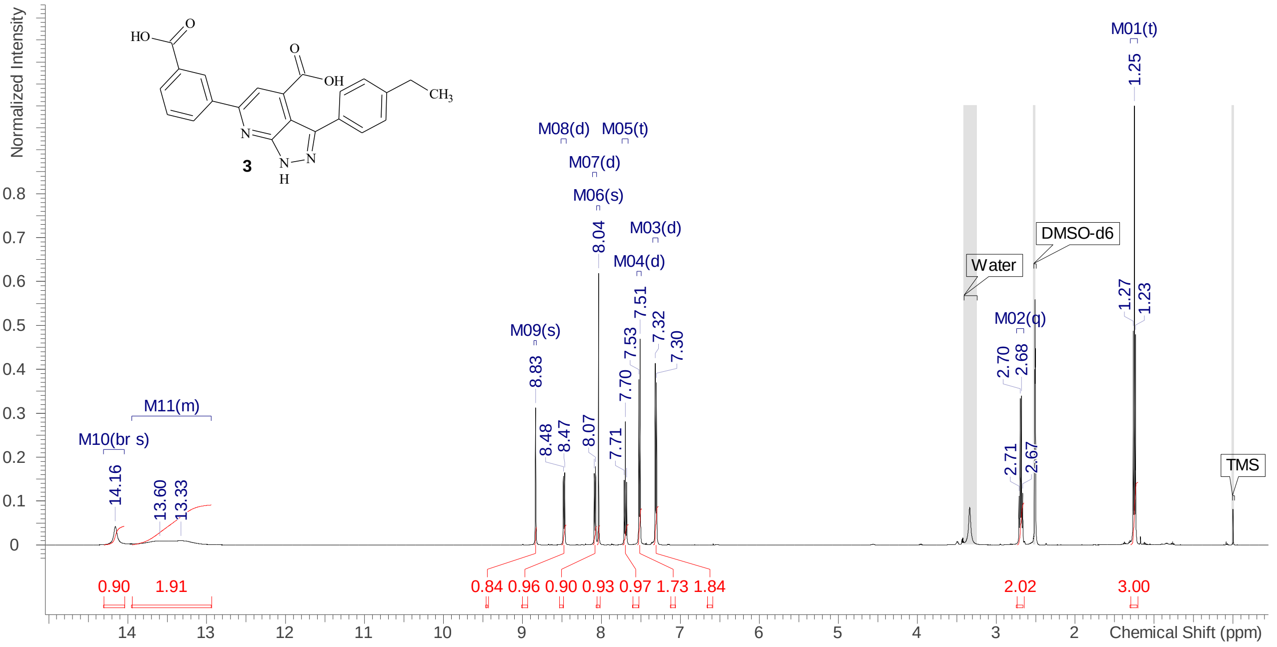
HO
O
O
OH
CH3
N
N
N
H
3
Normalized Intensity
M01(t)
1.25
M08(d)
M05(t)
M07(d)
M06(s)
8.04
M03(d)
M04(d)
7.51
7.53
7.32
7.30
M09(s)
8.83
7.70
8.48
8.47
8.07
7.71
M11(m)
M10(br s)
14.16
13.60
13.33
Water
DMSO-d6
M02(q)
2.70
2.68
2.71
2.67
1.27
1.23
TMS
0.90
1.91
0.84
0.96
0.90
0.93
0.97
1.73
1.84
2.02
3.00
Chemical Shift (ppm)

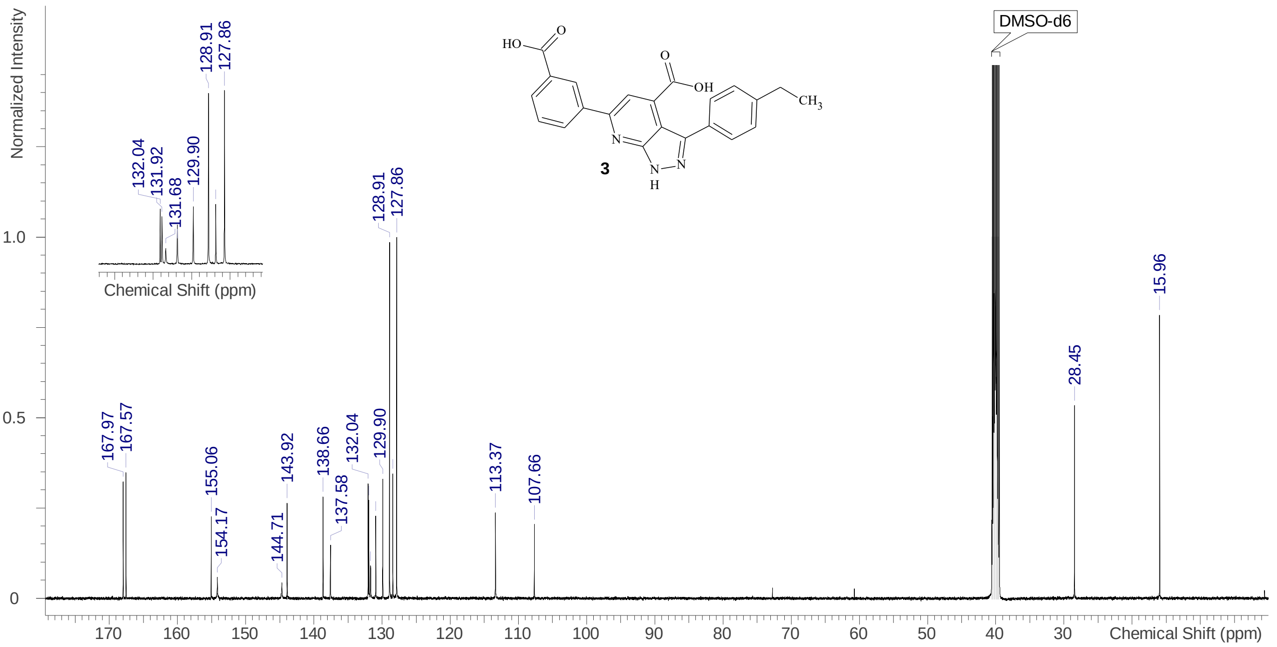
Normalized Intensity
DMSO-d6
128.91
127.86
132.04
131.92
131.68
129.90
Chemical Shift (ppm)
HO
O
O
OH
CH3
N
N
N
H
3
167.97
167.57
155.06
154.17
144.71
143.92
138.66
137.58
132.04
129.90
128.91
127.86
113.37
107.66
28.45
15.96
Chemical Shift (ppm)

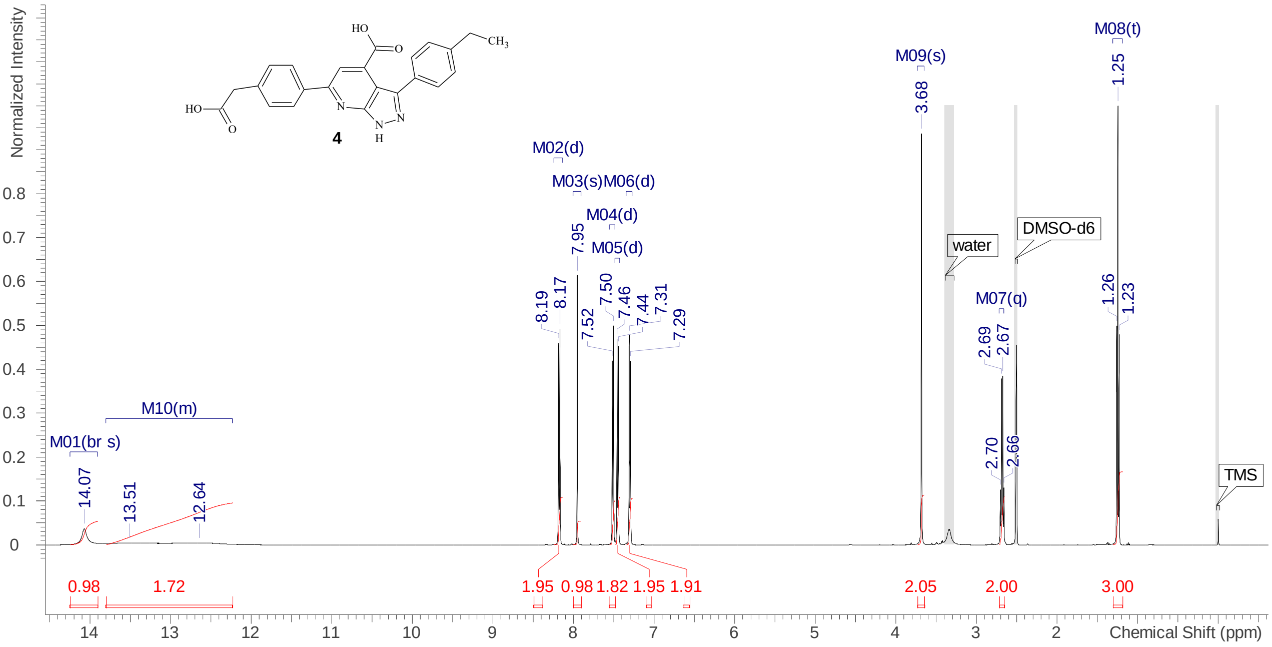
4
M01(br s)
M10(m)
M02(d)
M03(s)
M06(d)
M04(d)
M05(d)
M09(s)
M07(q)
M08(t)
water
DMSO-d6
TMS
14.07
13.51
12.64
8.19
8.17
7.95
7.52
7.50
7.46
7.44
7.31
7.29
3.68
2.69
2.67
2.70
2.66
1.26
1.25
1.23
0.98
1.72
1.95
0.98
1.82
1.95
1.91
2.05
2.00
3.00
Normalized Intensity
Chemical Shift (ppm)

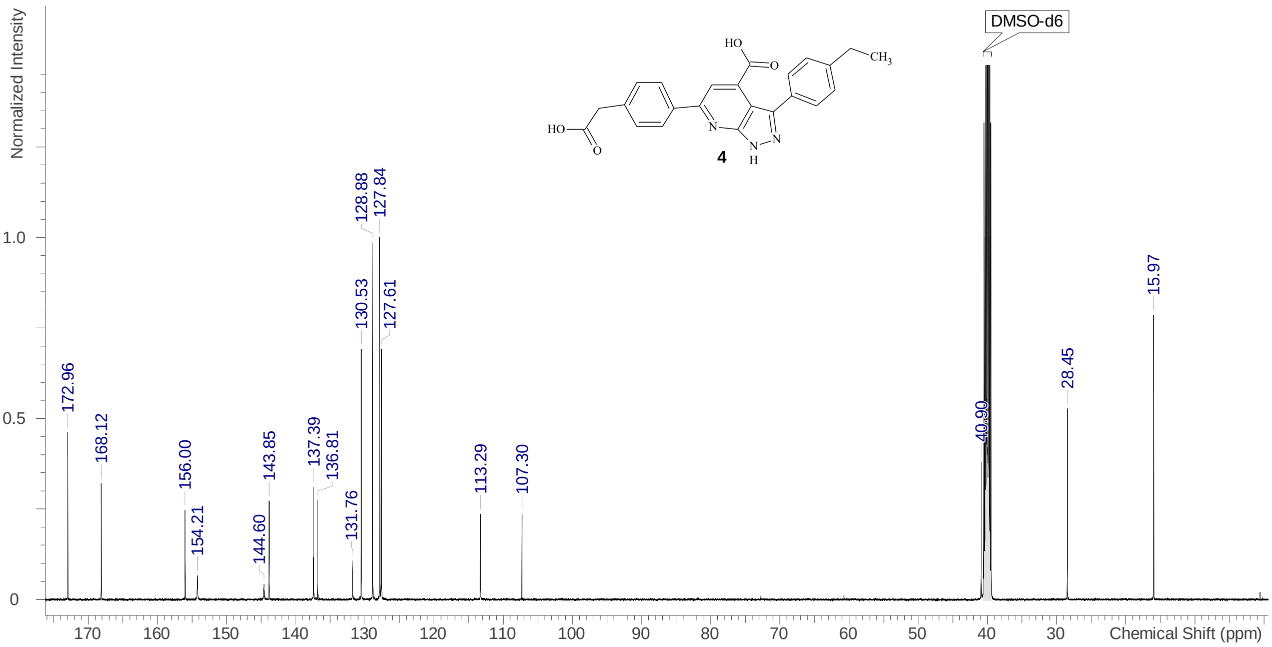
4
DMSO-d6
172.96
168.12
156.00
154.21
144.60
143.85
137.39
136.81
131.76
130.53
128.88
127.84
127.61
113.29
107.30
40.90
28.45
15.97
Normalized Intensity
Chemical Shift (ppm)

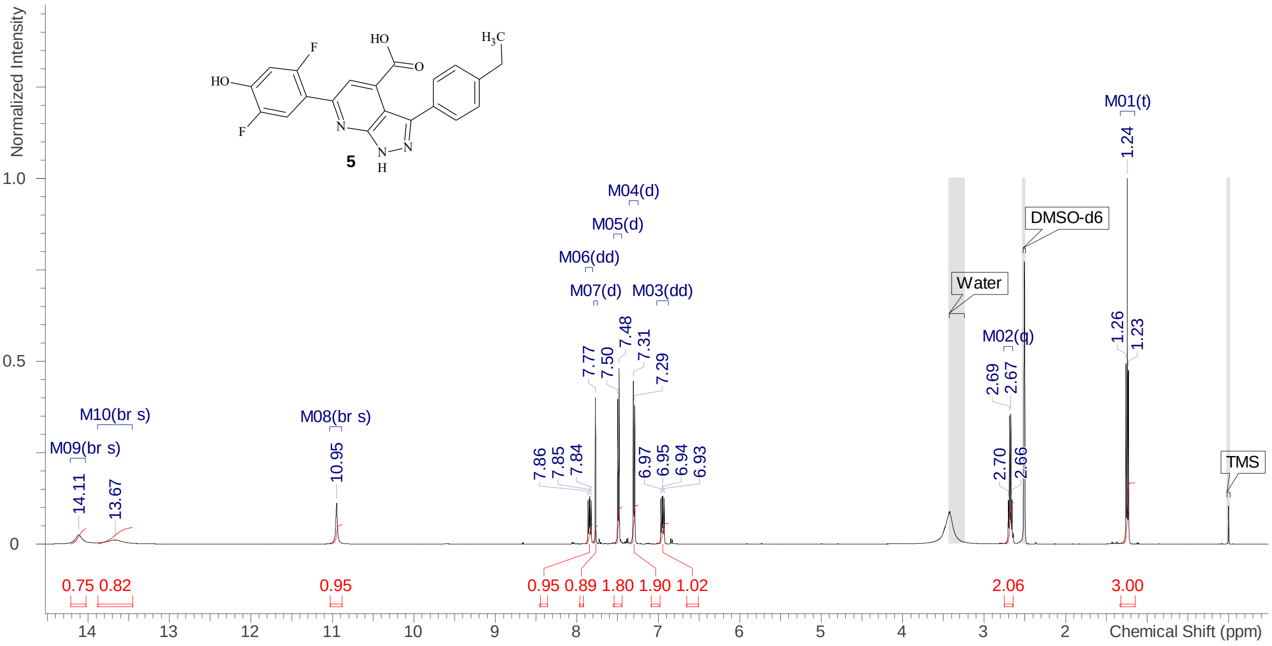
Normalized Intensity
5
M01(t)
1.24
M04(d)
M05(d)
M06(dd)
M07(d) M03(dd)
DMSO-d6
Water
M02(q)
M10(br s)
M09(br s)
M08(br s)
14.11
13.67
10.95
7.86
7.85
7.84
7.77
7.50
7.48
7.31
7.29
6.97
6.95
6.94
6.93
2.69
2.67
2.70
2.66
1.26
1.23
TMS
0.75 0.82
0.95
0.95 0.89 1.80 1.90 1.02
2.06
3.00
Chemical Shift (ppm)

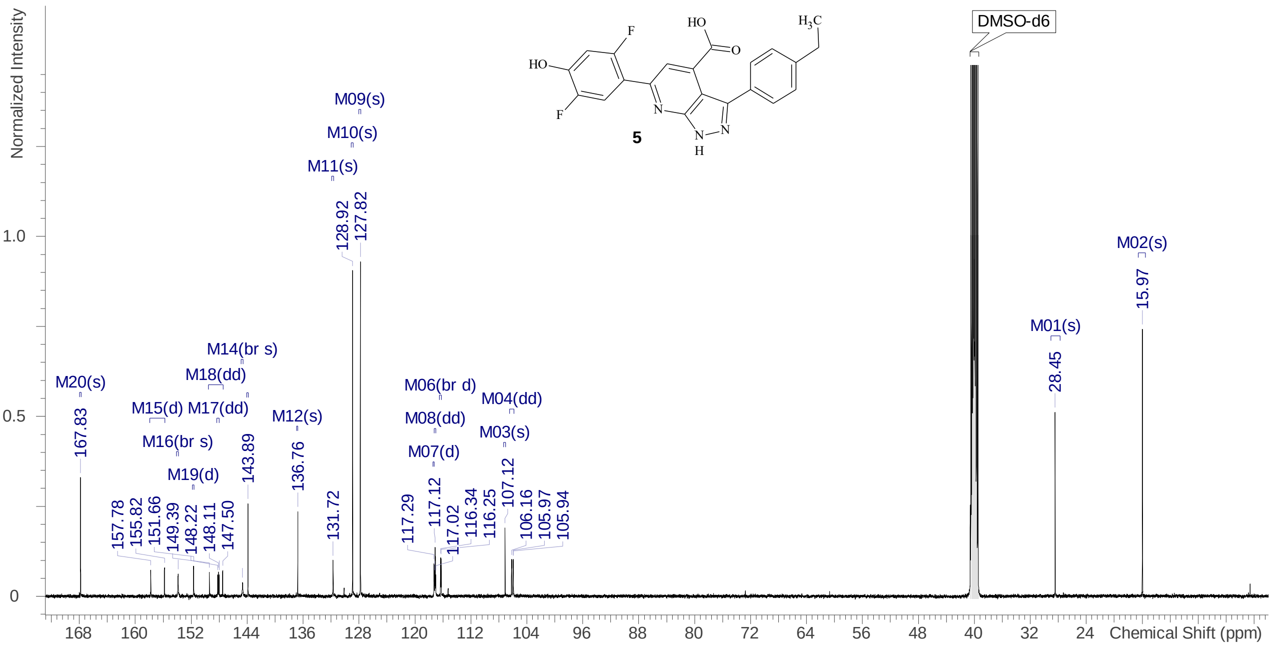
Normalized Intensity
5
DMSO-d6
M09(s)
M10(s)
M11(s)
128.92
127.82
M02(s)
15.97
M01(s)
28.45
M14(br s)
M18(dd)
M20(s)
M15(d) M17(dd)
M12(s)
M16(br s)
M19(d)
167.83
143.89
136.76
157.78
155.82
151.66
149.39
148.22
148.11
147.50
131.72
M06(br d)
M08(dd)
M07(d)
M04(dd)
M03(s)
117.29
117.12
117.02
116.34
116.25
107.12
106.16
105.97
105.94
Chemical Shift (ppm)

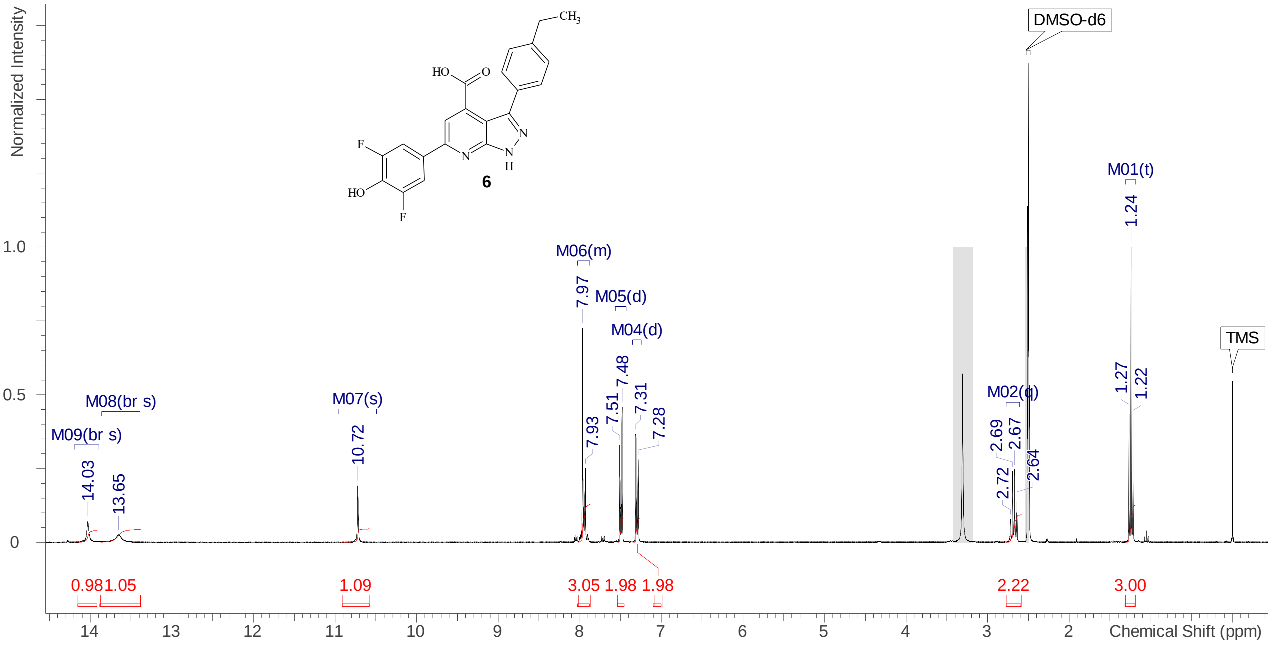
6
DMSO-d6
TMS
M01(t)
M02(q)
M04(d)
M05(d)
M06(m)
M07(s)
M08(br s)
M09(br s)
14.03
13.65
10.72
7.97
7.93
7.51
7.48
7.31
7.28
2.72
2.69
2.67
2.64
1.27
1.24
1.22
0.98
1.05
1.09
3.05
1.98
1.98
2.22
3.00
Normalized Intensity
Chemical Shift (ppm)

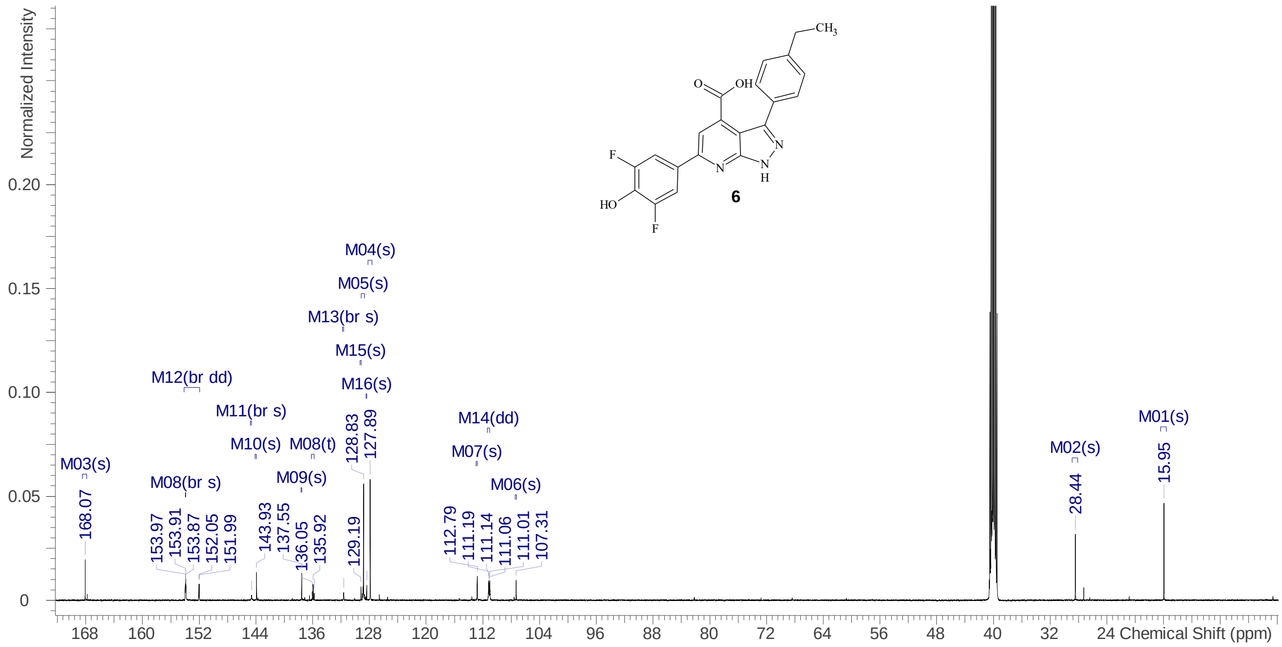
6
M01(s)
M02(s)
M03(s)
M04(s)
M05(s)
M06(s)
M07(s)
M08(br s)
M08(t)
M09(s)
M10(s)
M11(br s)
M12(br dd)
M13(br s)
M14(dd)
M15(s)
M16(s)
168.07
153.97
153.91
153.87
152.05
151.99
143.93
137.55
136.05
135.92
129.19
128.83
127.89
112.79
111.19
111.14
111.06
111.01
107.31
28.44
15.95
Normalized Intensity
Chemical Shift (ppm)

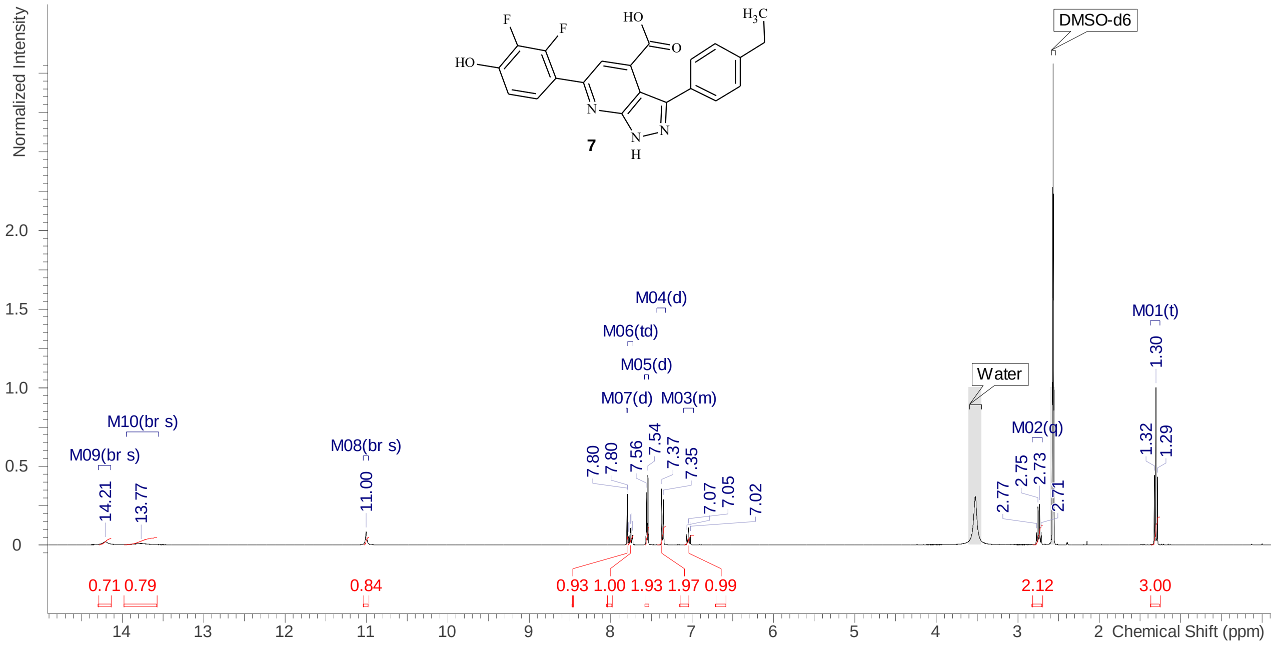

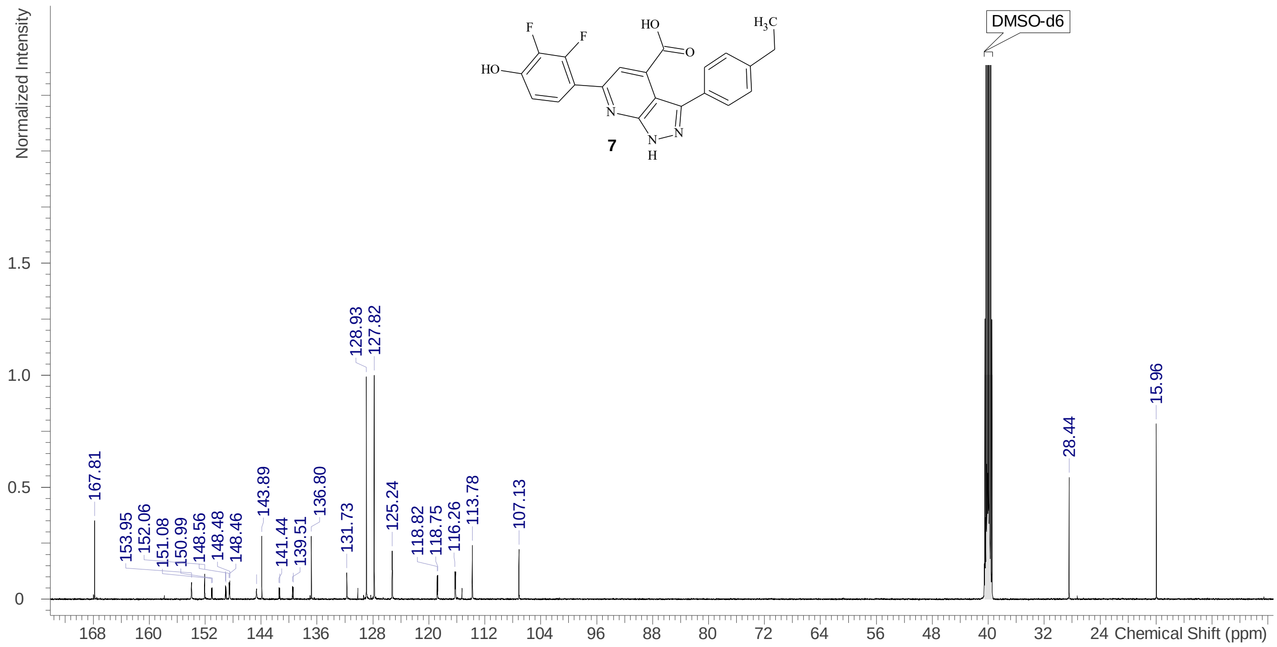

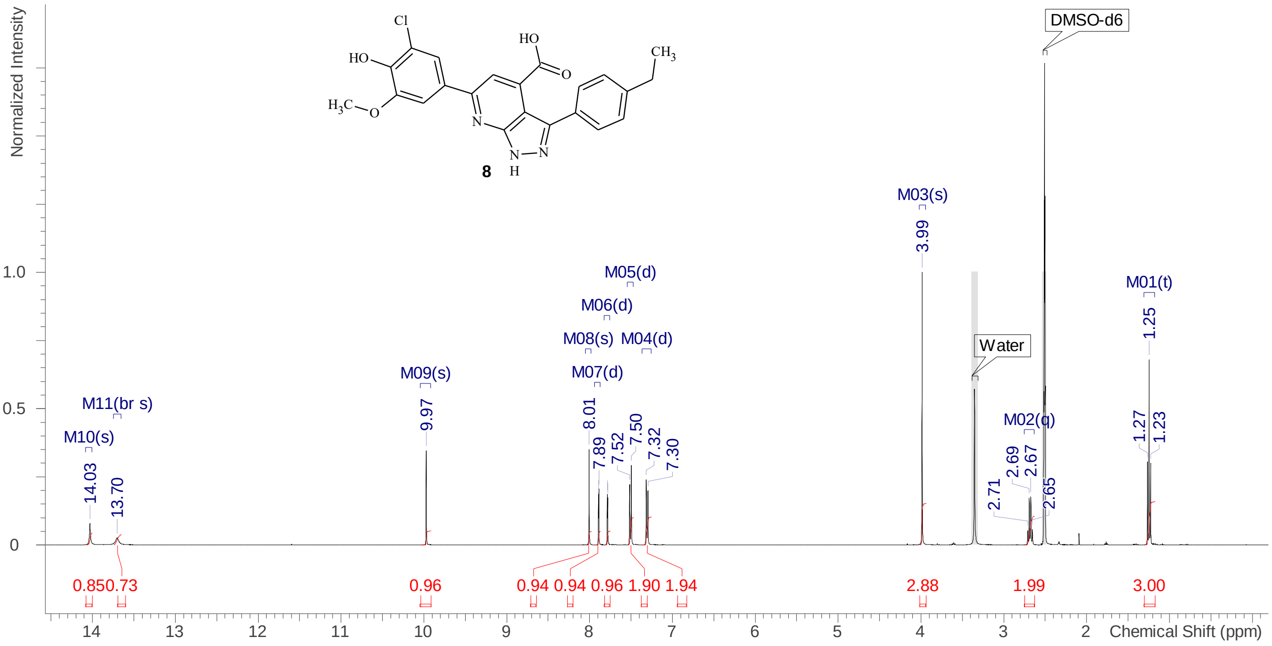
Cl
HO
H3C
O
HO
O
CH3
N
N
N
H
8
Normalized Intensity
Chemical Shift (ppm)
DMSO-d6
Water
M01(t)
M02(q)
M03(s)
M04(d)
M05(d)
M06(d)
M07(d)
M08(s)
M09(s)
M10(s)
M11(br s)
14.03
13.70
9.97
8.01
7.89
7.52
7.50
7.32
7.30
3.99
2.71
2.69
2.67
2.65
1.27
1.25
1.23
0.85
0.73
0.96
0.94
0.94
0.96
1.90
1.94
2.88
1.99
3.00

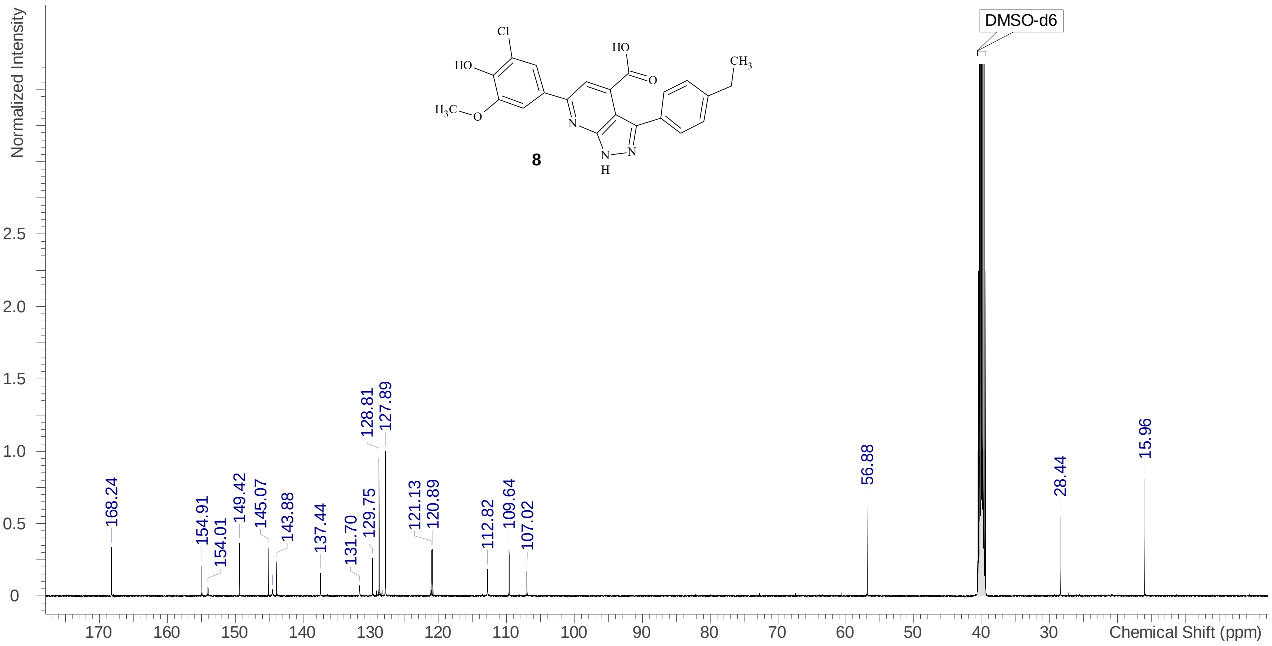
Cl
HO
H3C
O
HO
O
CH3
N
N
N
H
8
Normalized Intensity
Chemical Shift (ppm)
DMSO-d6
168.24
154.91
154.01
149.42
145.07
143.88
137.44
131.70
129.75
128.81
127.89
121.13
120.89
112.82
109.64
107.02
56.88
28.44
15.96

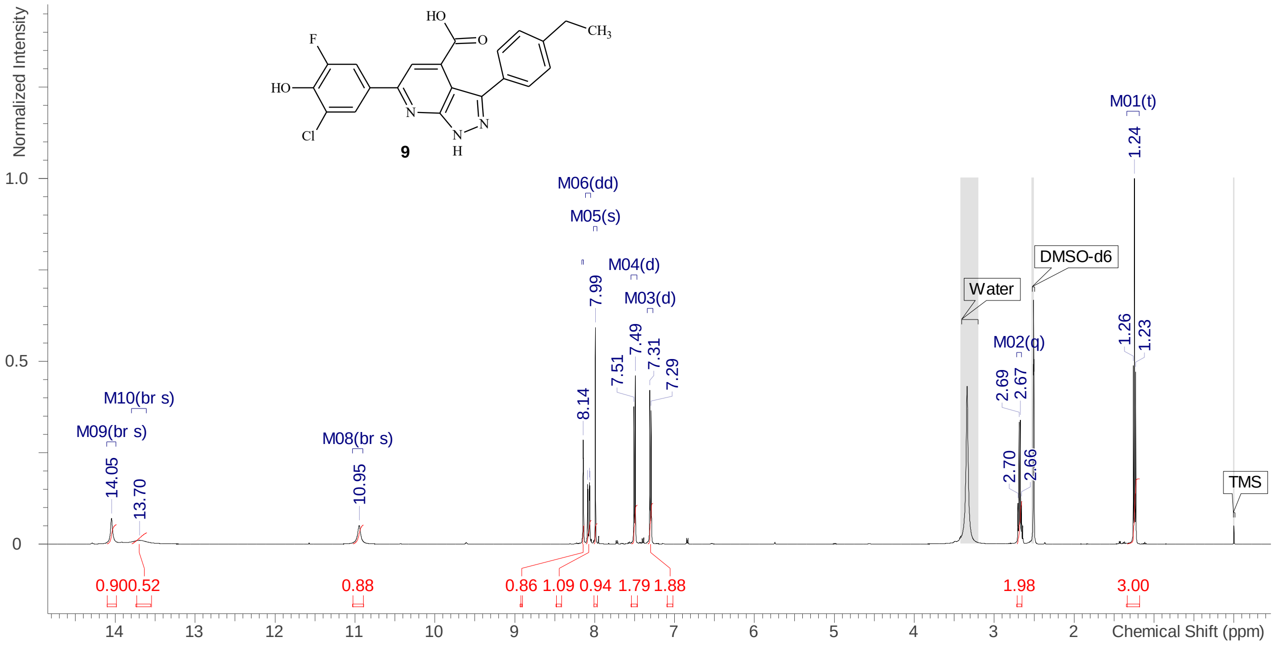

Normalized Intensity
9
M01(t)
M02(q)
M03(d)
M04(d)
M05(s)
M06(dd)
M08(br s)
M09(br s)
M10(br s)
Water
DMSO-d6
TMS
14.05
13.70
10.95
8.14
7.99
7.51
7.49
7.31
7.29
2.70
2.69
2.67
2.66
1.26
1.24
1.23
0.90
0.52
0.88
0.86
1.09
0.94
1.79
1.88
1.98
3.00
Chemical Shift (ppm)

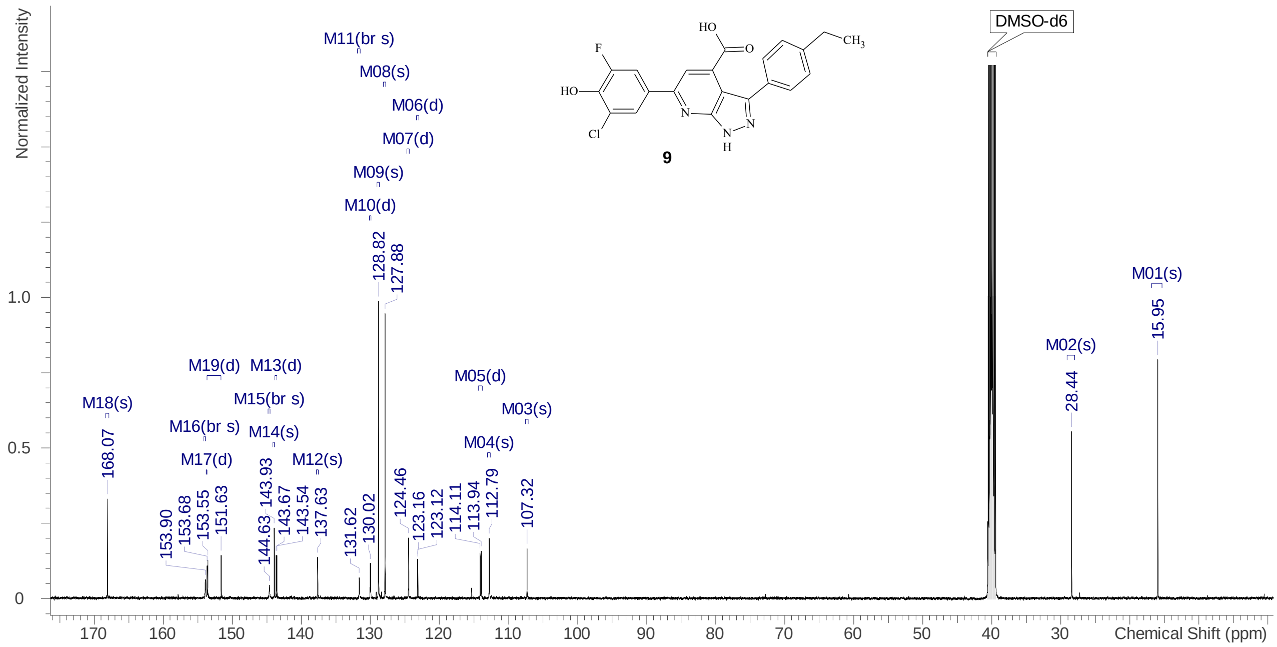

Normalized Intensity
9
DMSO-d6
M01(s)
M02(s)
M03(s)
M04(s)
M05(d)
M06(d)
M07(d)
M08(s)
M09(s)
M10(d)
M11(br s)
M12(s)
M13(d)
M14(s)
M15(br s)
M16(br s)
M17(d)
M18(s)
M19(d)
168.07
153.90
153.68
153.55
151.63
144.63
143.93
143.67
143.54
137.63
131.62
130.02
128.82
127.88
124.46
123.16
123.12
114.11
113.94
112.79
107.32
28.44
15.95
Chemical Shift (ppm)

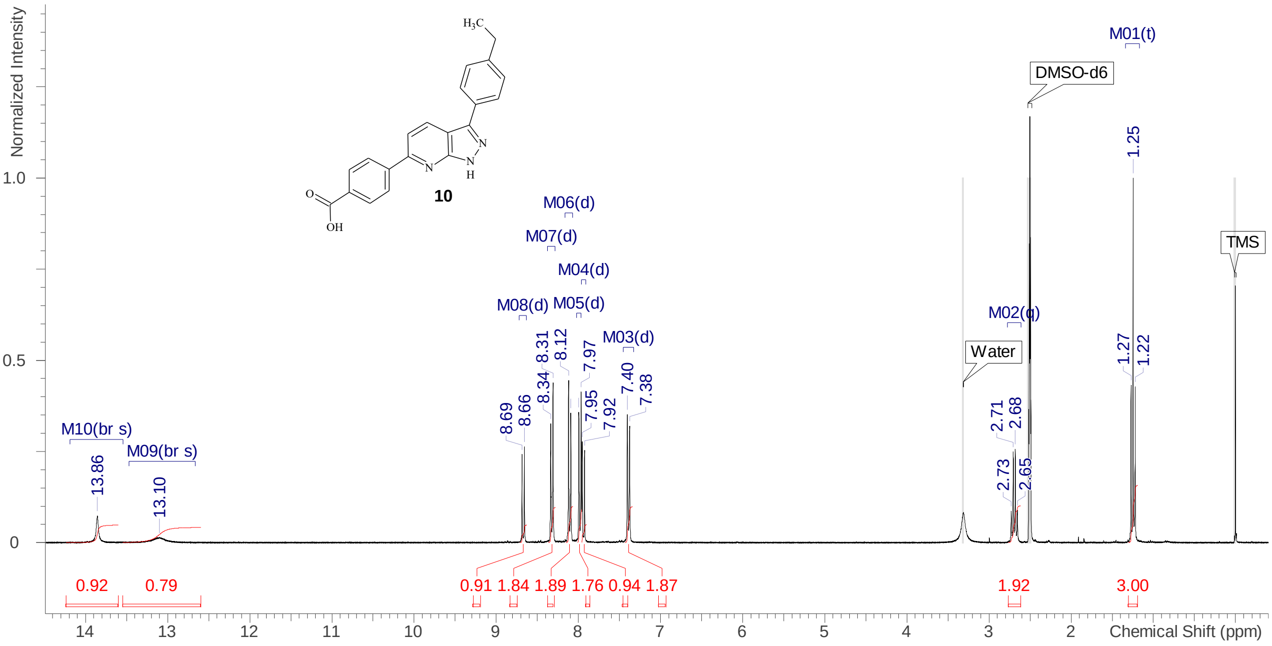
Normalized Intensity
H3C
N
N
N
H
O
OH
10
M06(d)
M07(d)
M04(d)
M08(d) M05(d)
M03(d)
M10(br s)
M09(br s)
13.86
13.10
8.69
8.66
8.34
8.31
8.12
7.97
7.95
7.92
7.40
7.38
Water
M02(q)
2.71
2.68
2.73
2.65
DMSO-d6
M01(t)
1.25
1.27
1.22
TMS
0.92 0.79 0.91 1.84 1.89 1.76 0.94 1.87 1.92 3.00
Chemical Shift (ppm)

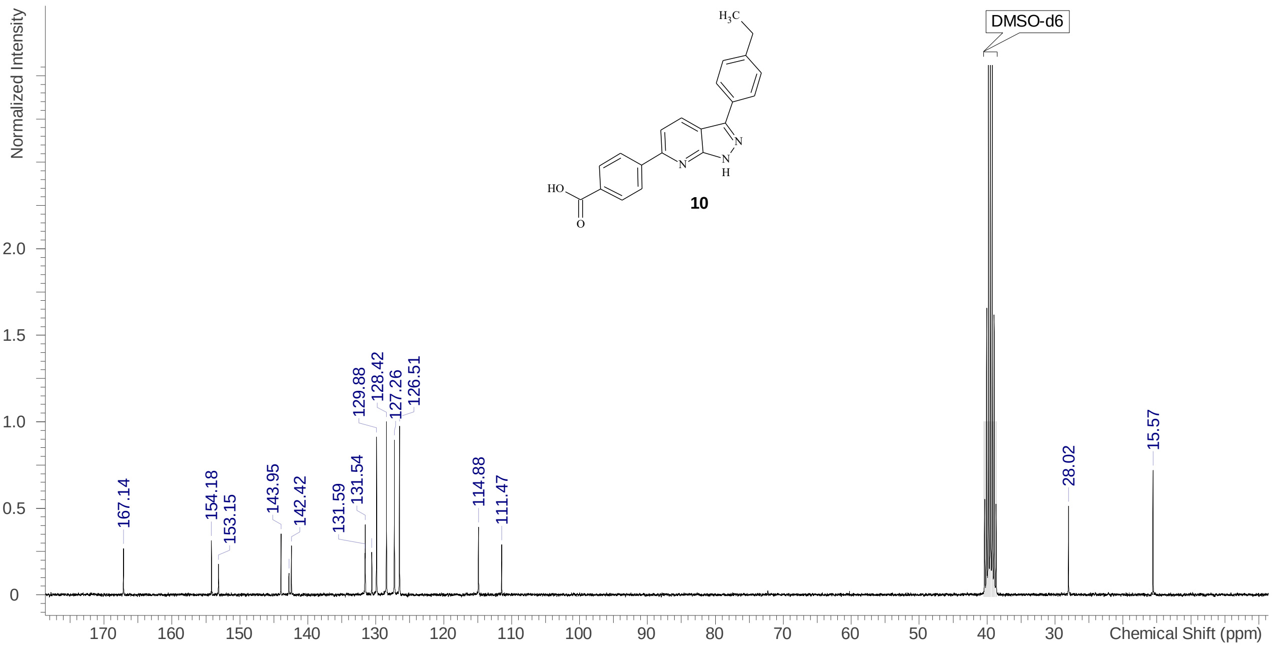
Normalized Intensity
H3C
N
N
N
H
HO
O
10
DMSO-d6
167.14
154.18
153.15
143.95
142.42
131.59
131.54
129.88
128.42
127.26
126.51
114.88
111.47
28.02
15.57
Chemical Shift (ppm)

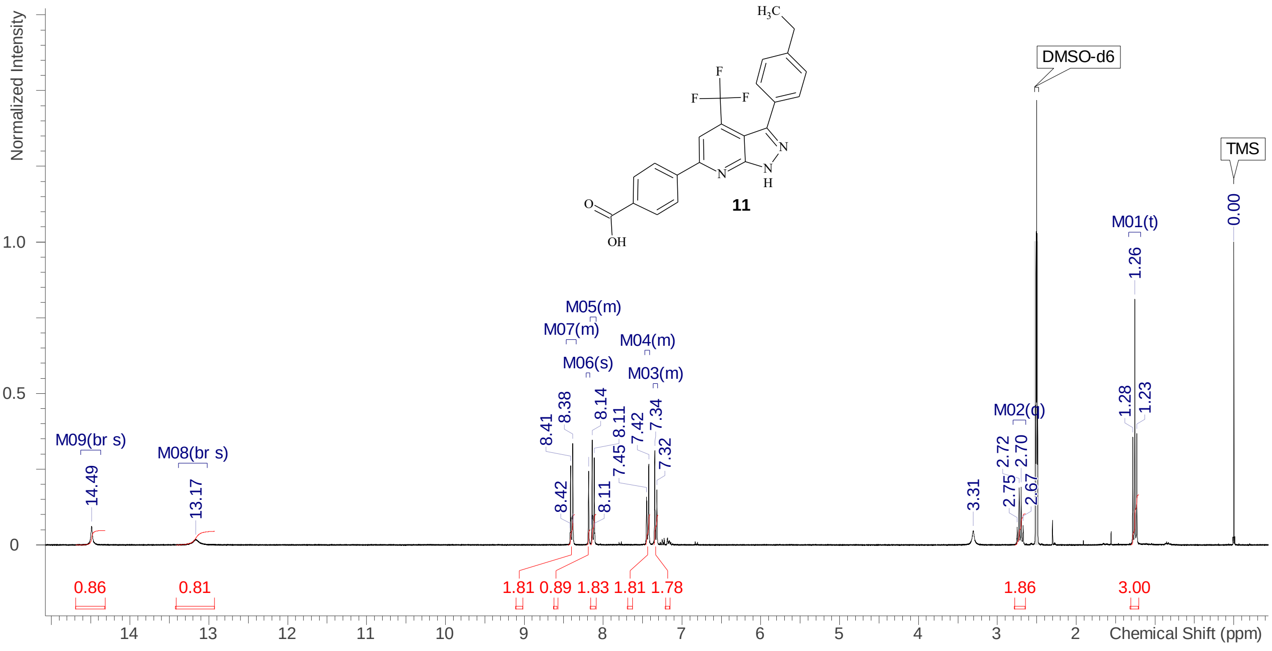

Normalized Intensity
Chemical Shift (ppm)
DMSO-d6
TMS
11
M01(t)
M02(q)
M03(m)
M04(m)
M05(m)
M06(s)
M07(m)
M08(br s)
M09(br s)
14.49
13.17
8.42
8.41
8.38
8.14
8.11
8.11
7.45
7.42
7.34
7.32
3.31
2.75
2.72
2.70
2.67
1.28
1.26
1.23
0.00
0.86
0.81
1.81
0.89
1.83
1.81
1.78
1.86
3.00

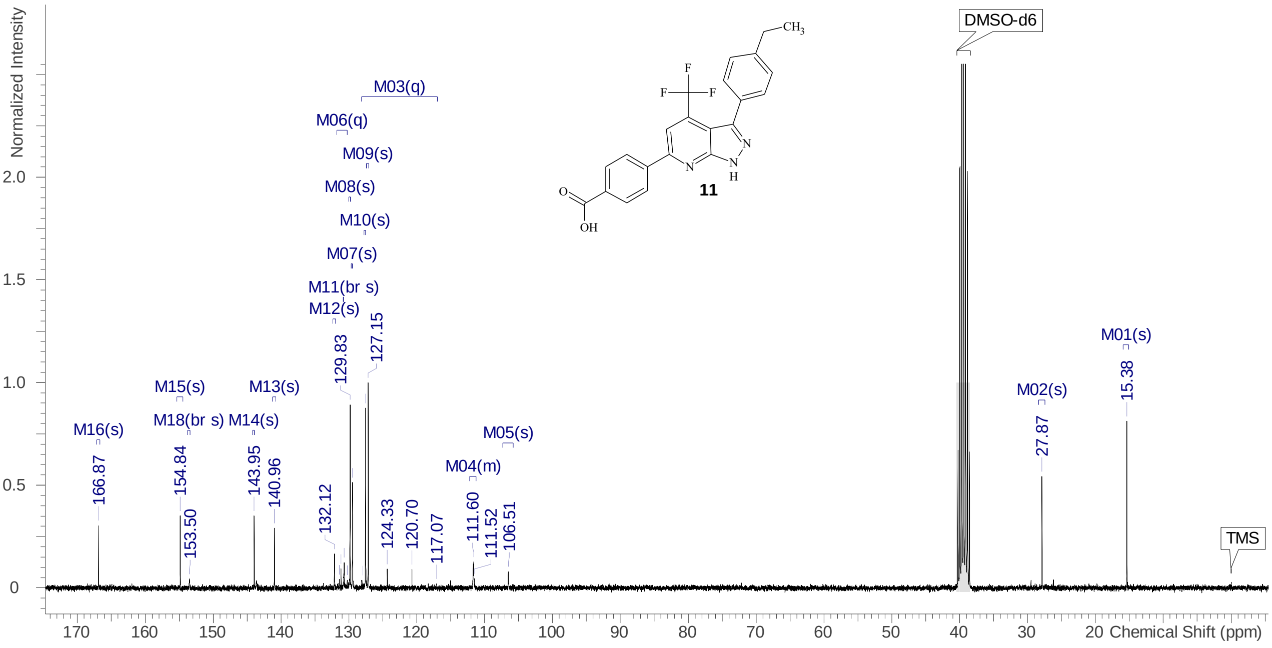

Normalized Intensity
Chemical Shift (ppm)
DMSO-d6
TMS
11
M01(s)
M02(s)
M03(q)
M04(m)
M05(s)
M06(q)
M07(s)
M08(s)
M09(s)
M10(s)
M11(br s)
M12(s)
M13(s)
M14(s)
M15(s)
M16(s)
M18(br s)
166.87
154.84
153.50
143.95
140.96
132.12
129.83
127.15
124.33
120.70
117.07
111.60
111.52
106.51
27.87
15.38

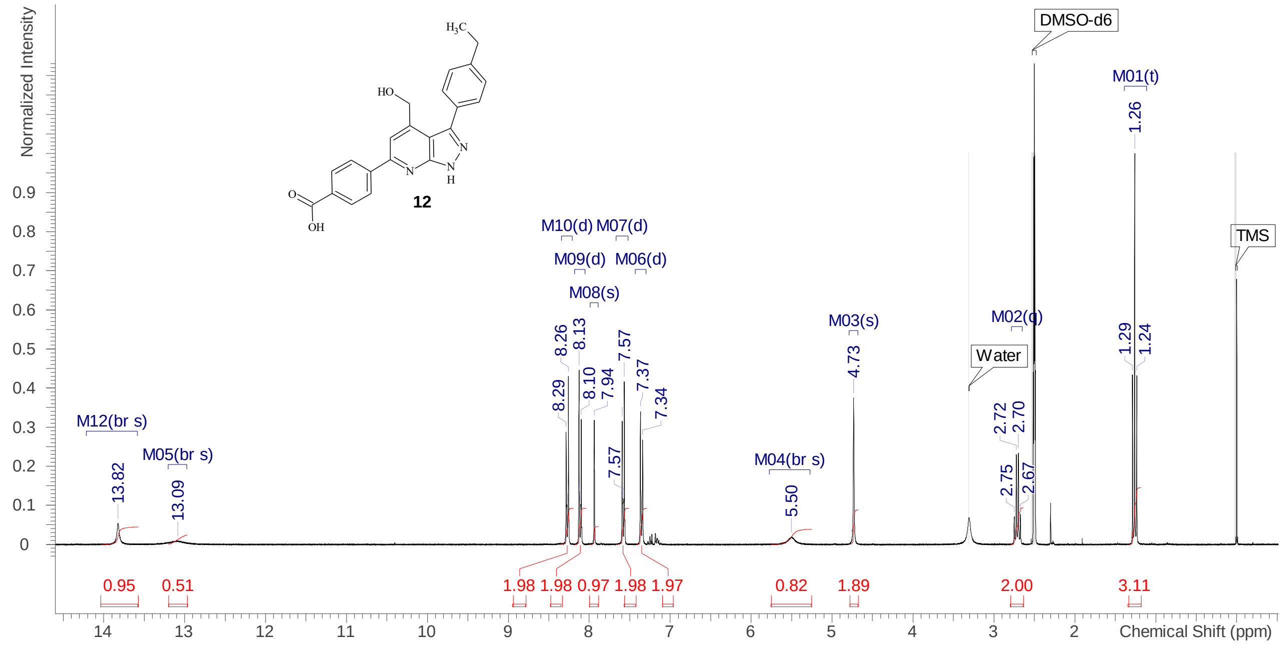

12
M12(br s)
M05(br s)
M10(d) M07(d)
M09(d) M06(d)
M08(s)
M04(br s)
M03(s)
M02(q)
M01(t)
DMSO-d6
Water
TMS
Normalized Intensity
Chemical Shift (ppm)

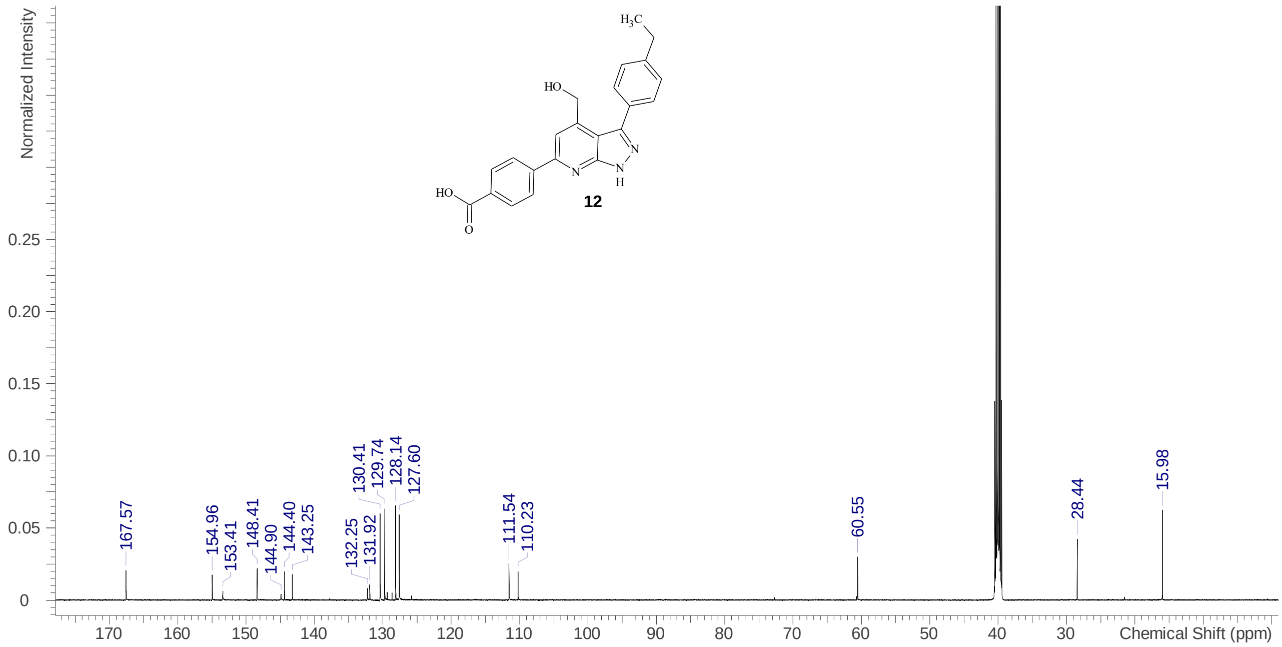

12
Normalized Intensity
Chemical Shift (ppm)

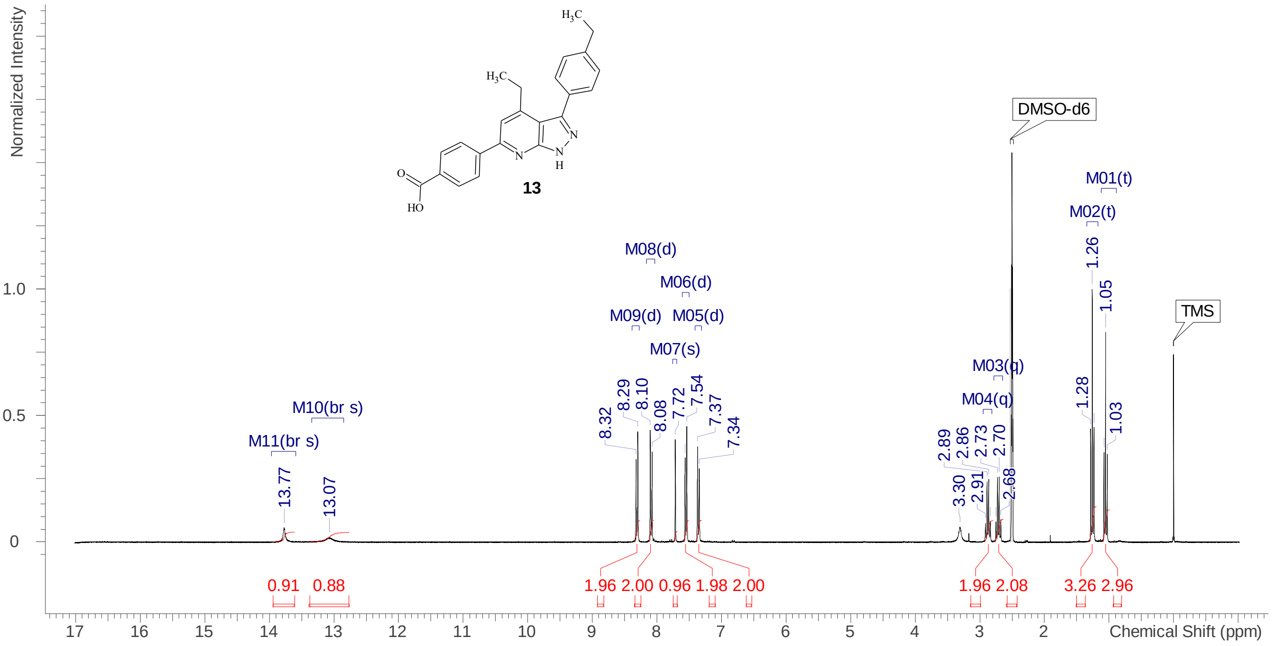
13
DMSO-d6
TMS
M01(t)
M02(t)
M03(q)
M04(q)
M05(d)
M06(d)
M07(s)
M08(d)
M09(d)
M10(br s)
M11(br s)
Normalized Intensity
Chemical Shift (ppm)
0.91 0.88 1.96 2.00 0.96 1.98 2.00 1.96 2.08 3.26 2.96

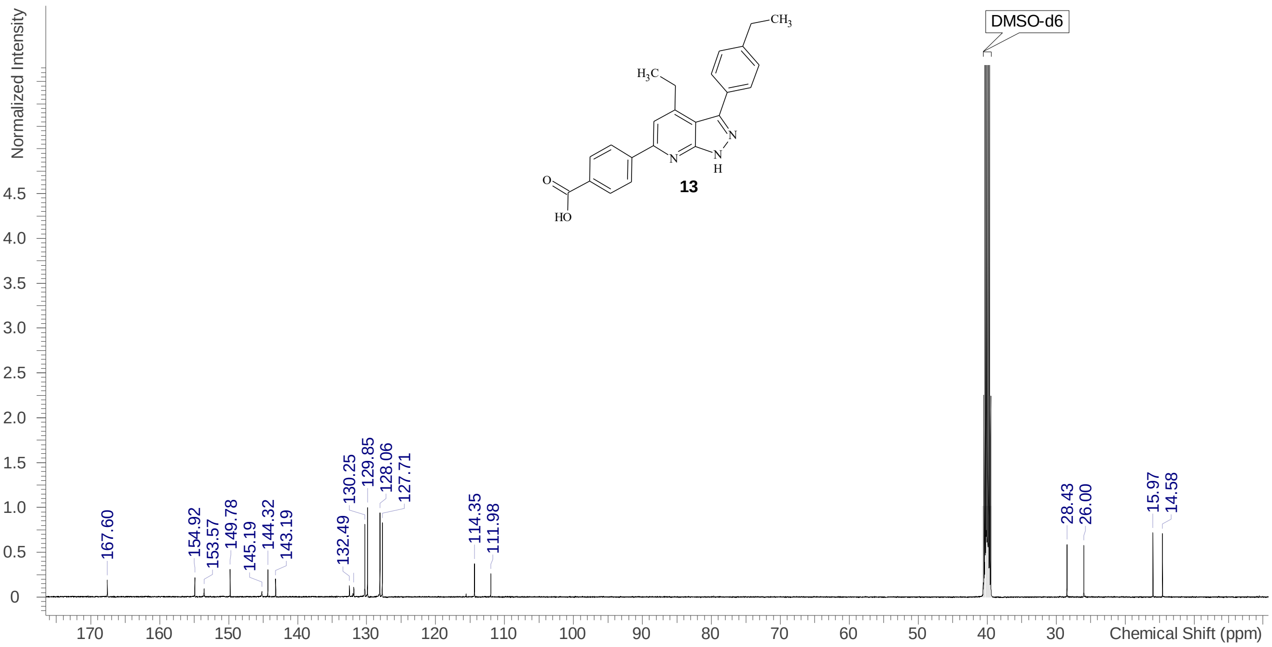
13
DMSO-d6
Normalized Intensity
Chemical Shift (ppm)
167.60
154.92
153.57
149.78
145.19
144.32
143.19
132.49
130.25
129.85
128.06
127.71
114.35
111.98
28.43
26.00
15.97
14.58

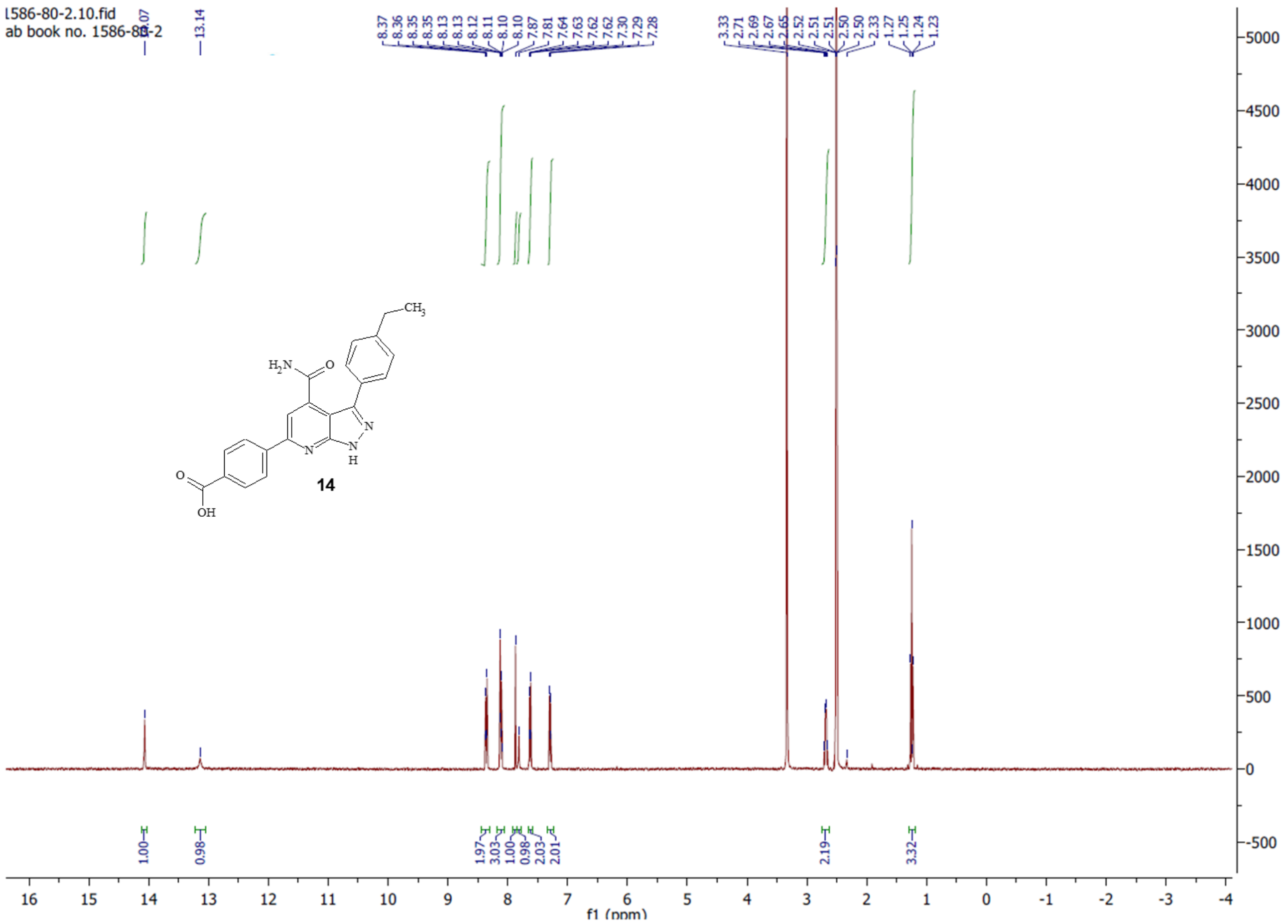
1586-80-2.10.fid
ab book no. 1586-80-2
14.07
13.14
8.37 8.36 8.35 8.35 8.13 8.13 8.12 8.11 8.10 8.10 7.87 7.81 7.64 7.63 7.62 7.62 7.30 7.29 7.28
3.33 2.71 2.69 2.67 2.65 2.52 2.51 2.51 2.50 2.50 2.33 1.27 1.25 1.24 1.23
CH_3
H_2N
O
N
N
H
OH
14
1.00
0.98
1.97 3.03 1.00 0.98 2.03 2.01
2.19
3.32
f1 (ppm)

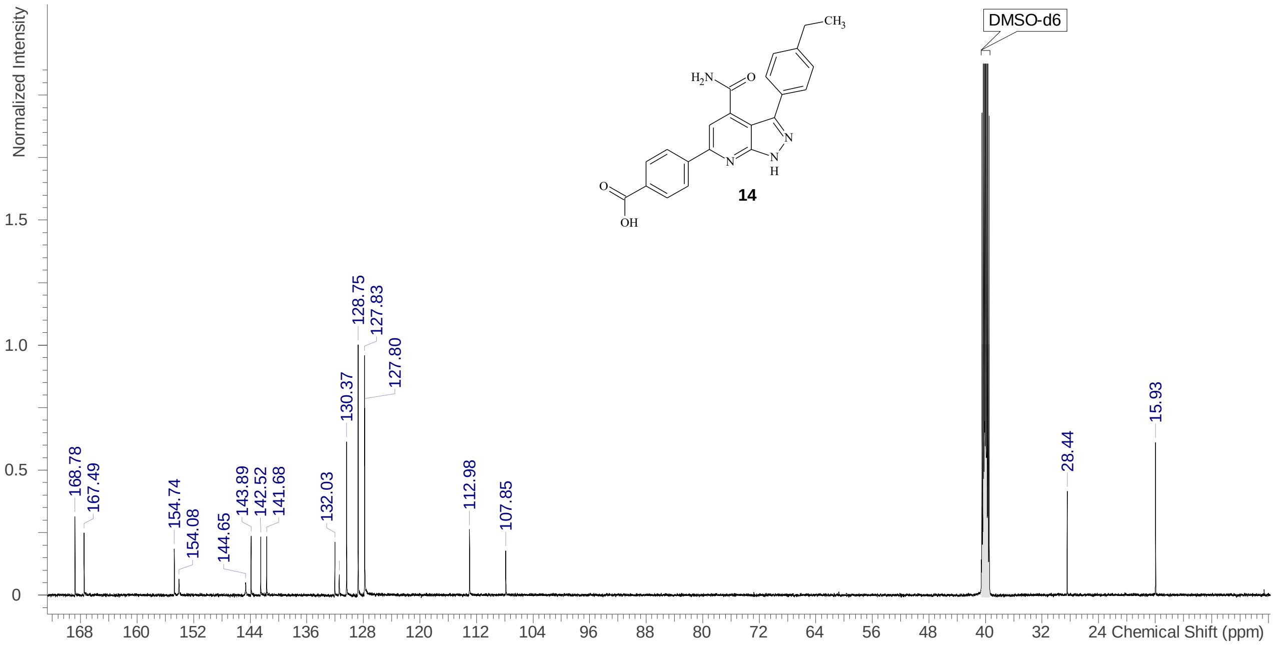
Normalized Intensity
DMSO-d6
CH_3
H_2N
O
N
N
H
OH
14
168.78
167.49
154.74
154.08
144.65
143.89
142.52
141.68
132.03
130.37
128.75
127.83
127.80
112.98
107.85
28.44
15.93
Chemical Shift (ppm)

HPLC and LC/MS analysis of final ligands **1**-**14**

Compound 1

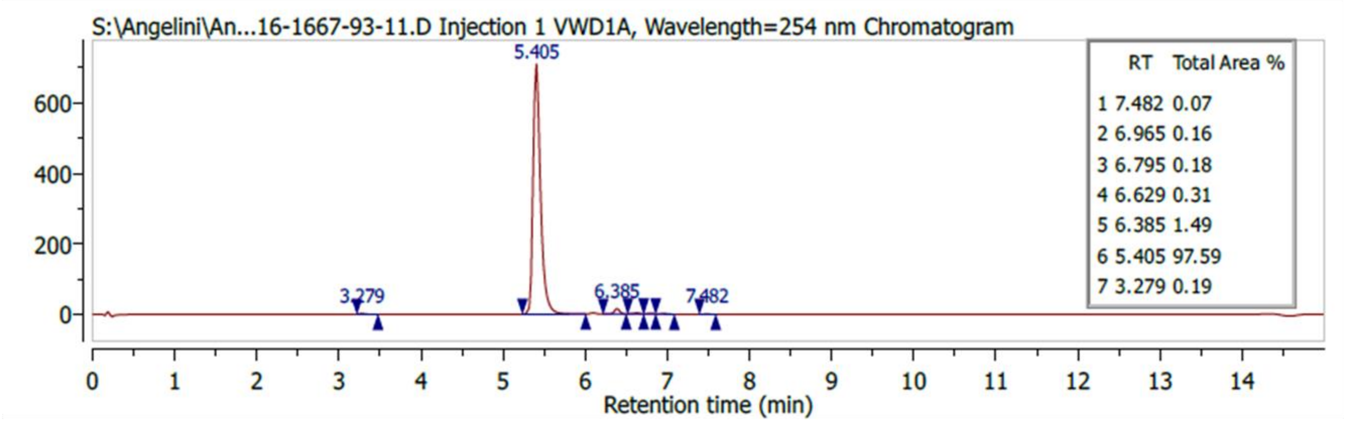

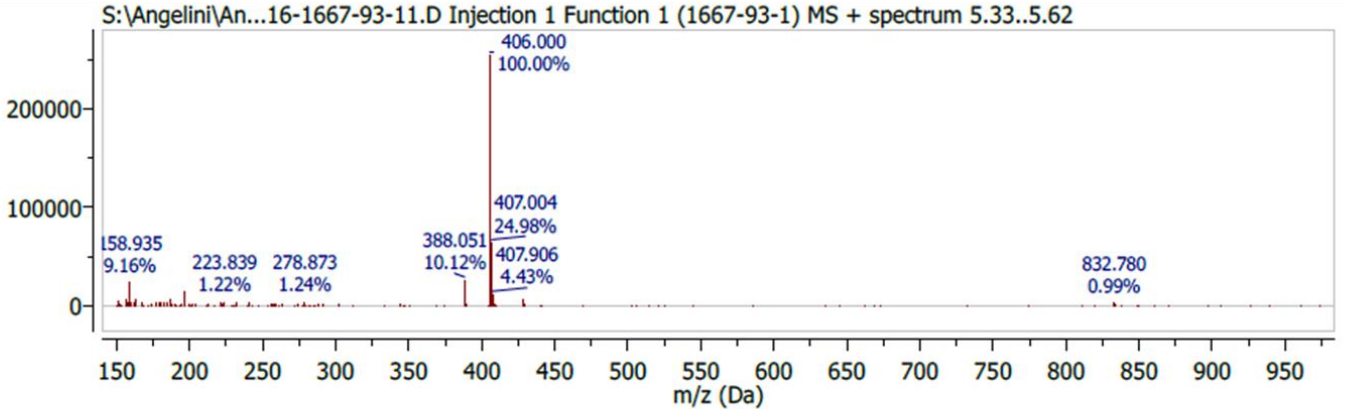

Compound 2

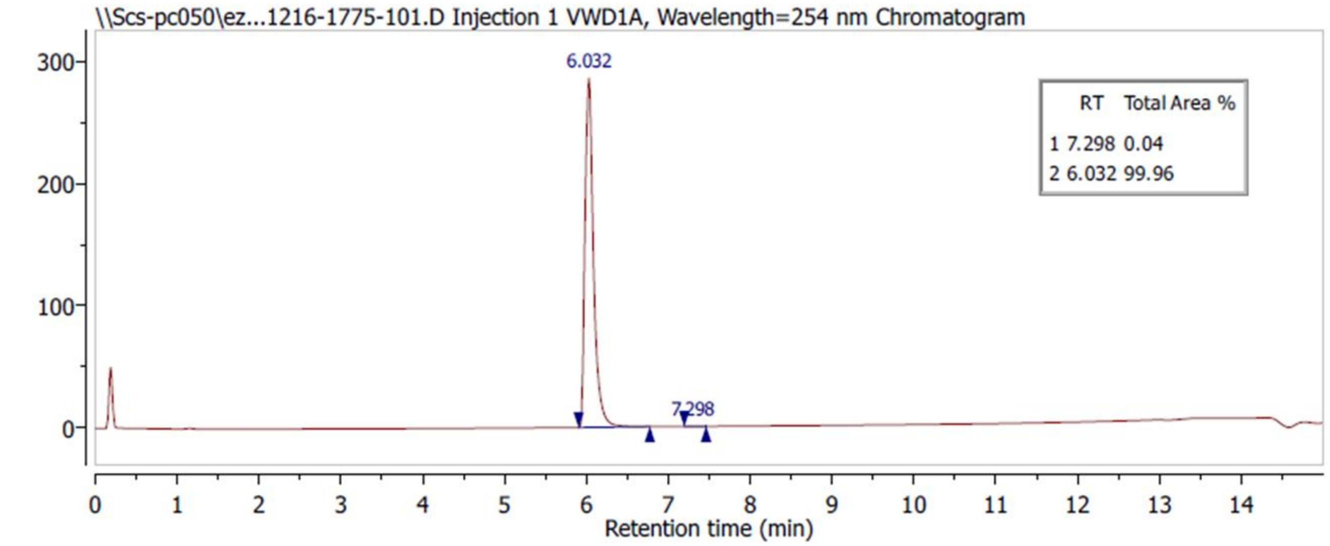
\\Scs-pc050\ez...1216-1775-101.D Injection 1 VWD1A, Wavelength=254 nm Chromatogram
6.032
7.298
RT Total Area %
1 7.298 0.04
2 6.032 99.96
Retention time (min)

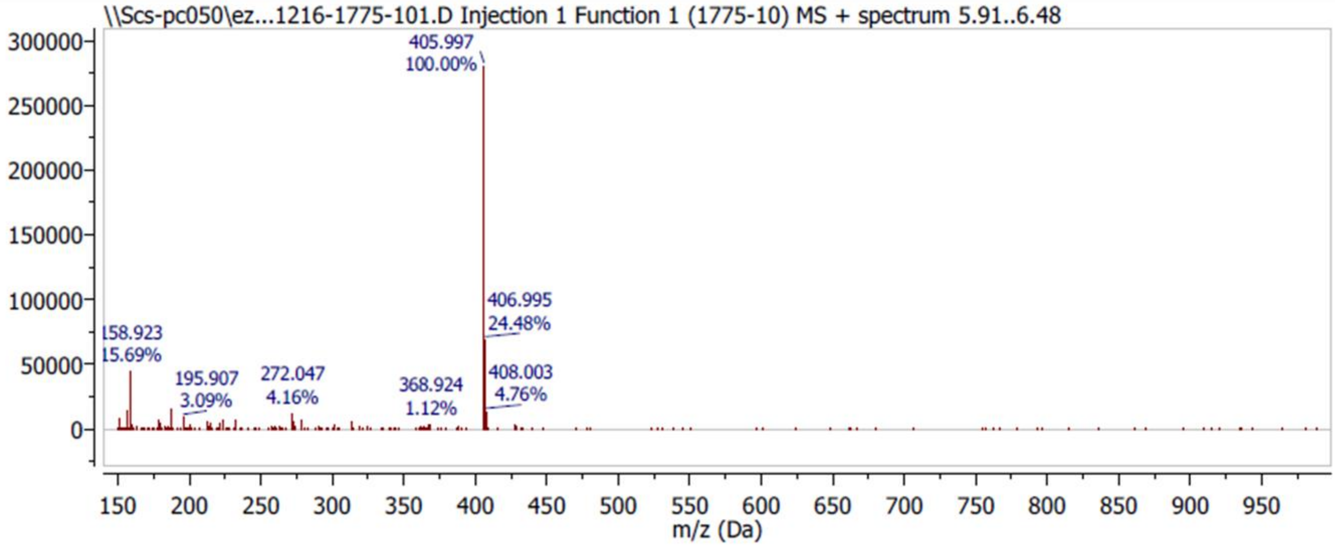
\\Scs-pc050\ez...1216-1775-101.D Injection 1 Function 1 (1775-10) MS + spectrum 5.91..6.48
405.997
100.00%
406.995
24.48%
408.003
4.76%
158.923
15.69%
195.907
3.09%
272.047
4.16%
368.924
1.12%
m/z (Da)

Compound 3

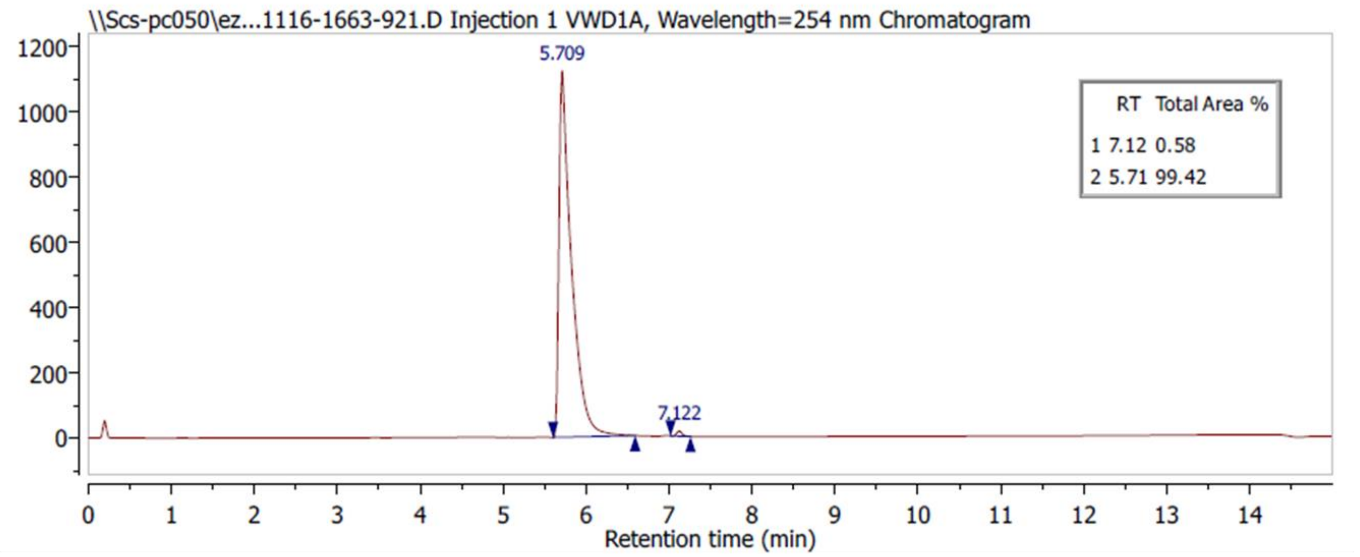

\\Scs-pc050\ez...1116-1663-921.D Injection 1 VWD1A, Wavelength=254 nm Chromatogram
5.709
7.122
RT Total Area %
1 7.12 0.58
2 5.71 99.42
Retention time (min)

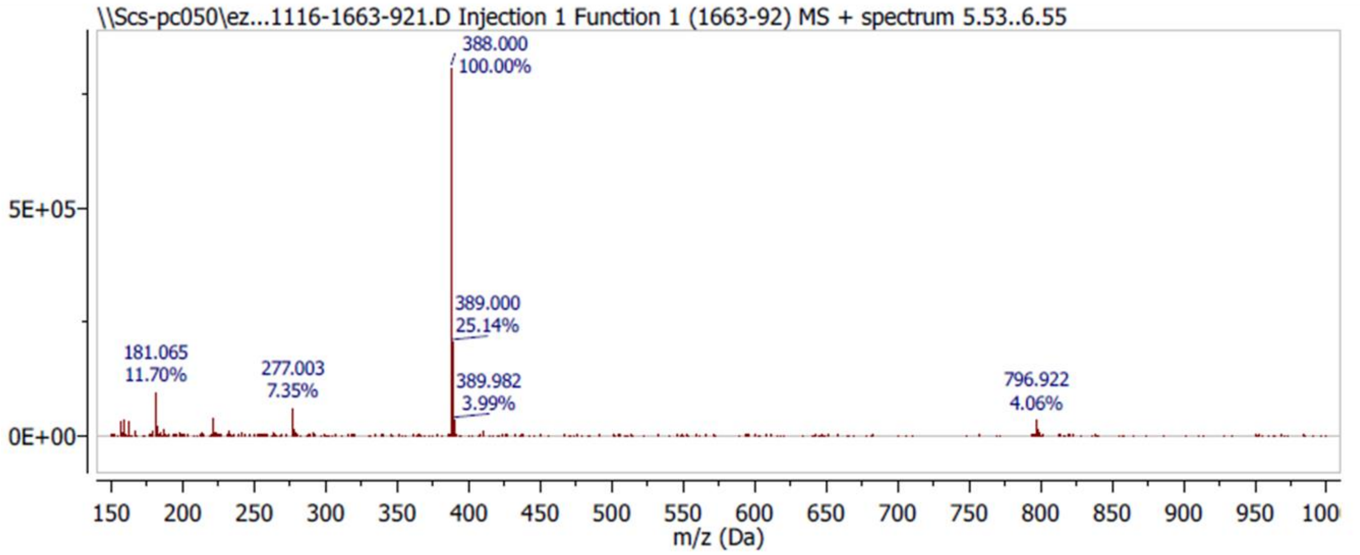

\\Scs-pc050\ez...1116-1663-921.D Injection 1 Function 1 (1663-92) MS + spectrum 5.53..6.55
388.000
100.00%
389.000
25.14%
389.982
3.99%
181.065
11.70%
277.003
7.35%
796.922
4.06%
m/z (Da)

Compound 4

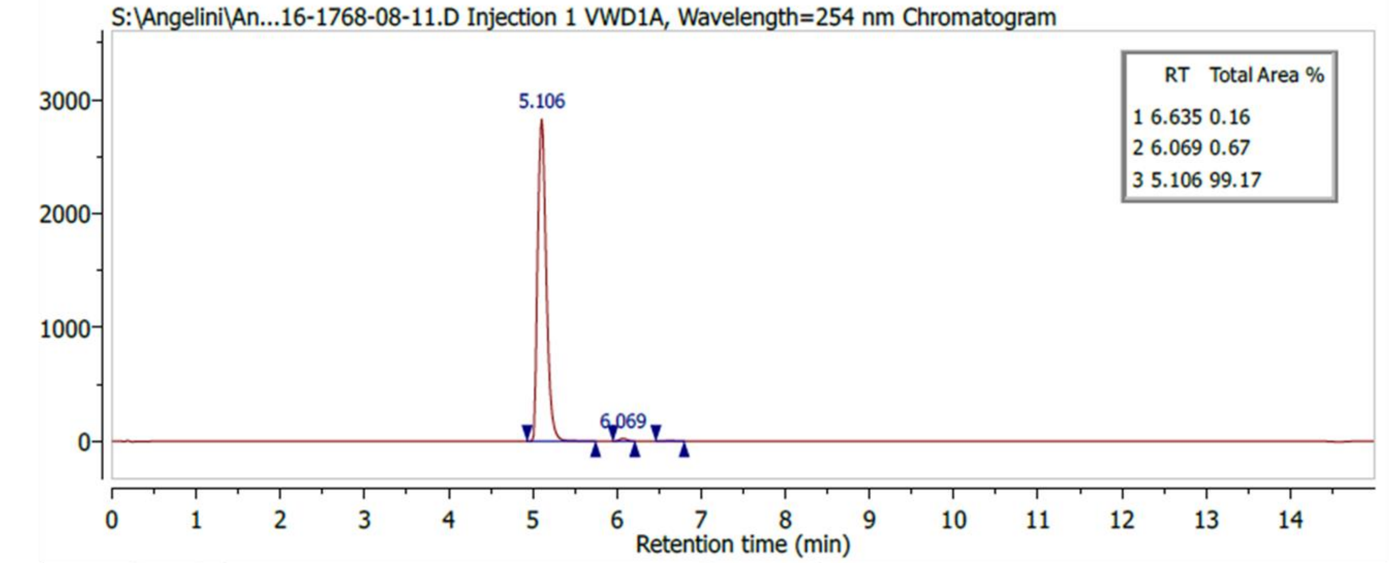
S:\Angelini\An...16-1768-08-11.D Injection 1 VWD1A, Wavelength=254 nm Chromatogram
RT Total Area %
1 6.635 0.16
2 6.069 0.67
3 5.106 99.17
5.106
6.069
Retention time (min)

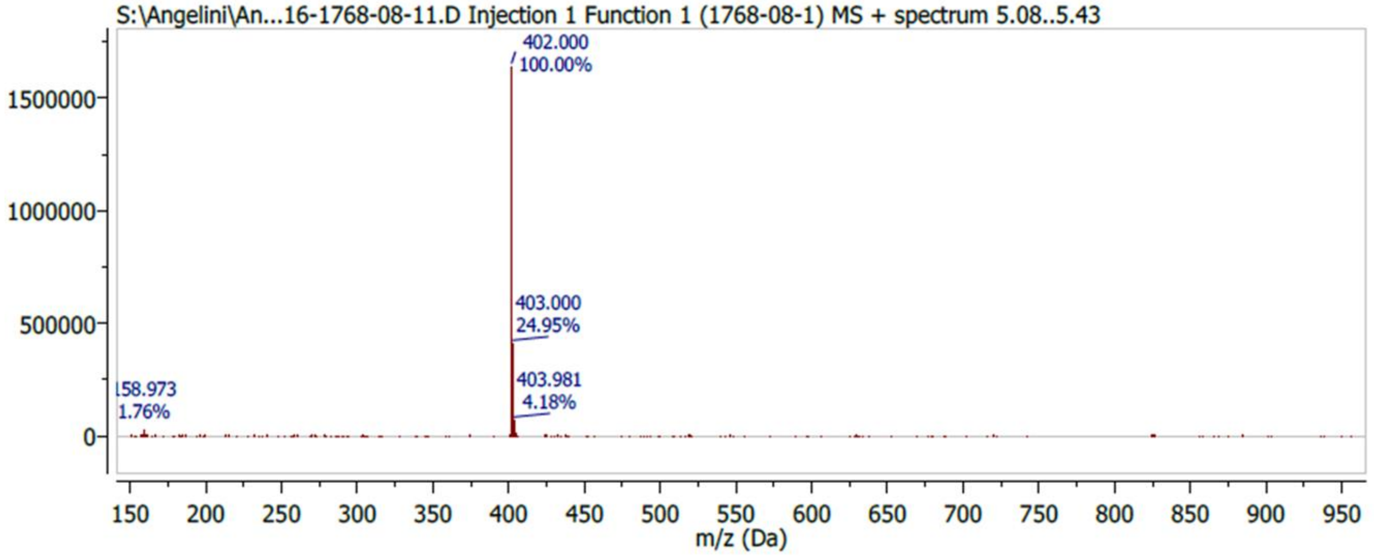
S:\Angelini\An...16-1768-08-11.D Injection 1 Function 1 (1768-08-1) MS + spectrum 5.08..5.43
402.000
100.00%
403.000
24.95%
403.981
4.18%
158.973
1.76%
m/z (Da)

Compound 5

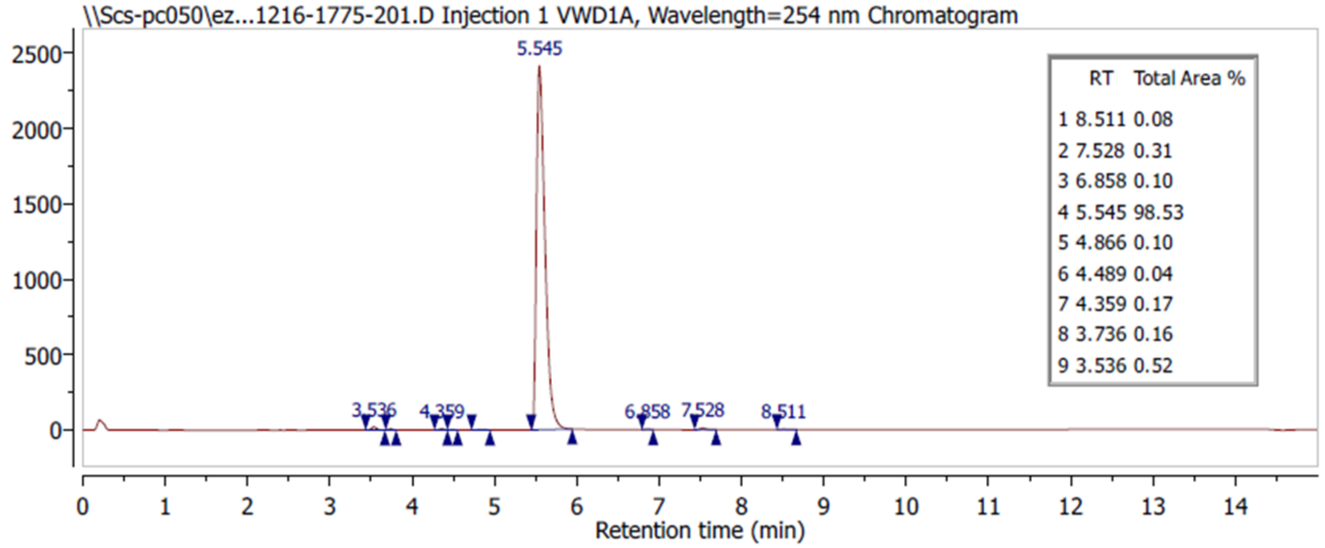

\\Scs-pc050\ez...1216-1775-201.D Injection 1 VWD1A, Wavelength=254 nm Chromatogram
5.545
RT Total Area %
1 8.511 0.08
2 7.528 0.31
3 6.858 0.10
4 5.545 98.53
5 4.866 0.10
6 4.489 0.04
7 4.359 0.17
8 3.736 0.16
9 3.536 0.52
3.536
4.359
6.858
7.528
8.511
Retention time (min)

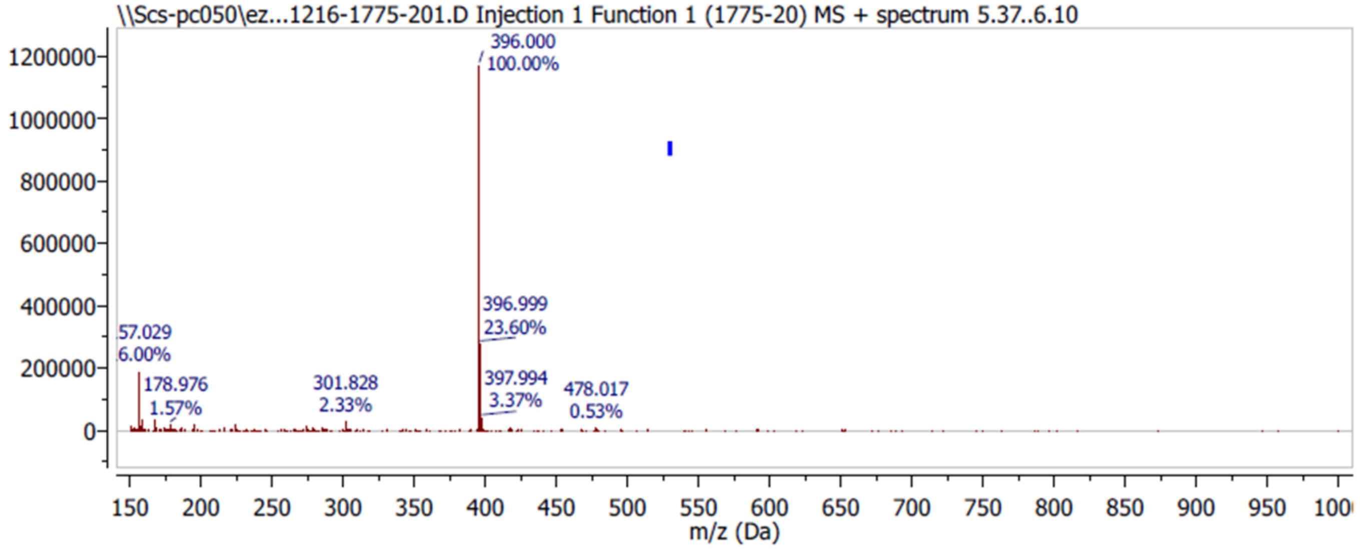

\\Scs-pc050\ez...1216-1775-201.D Injection 1 Function 1 (1775-20) MS + spectrum 5.37..6.10
396.000
100.00%
396.999
23.60%
157.029
6.00%
178.976
1.57%
301.828
2.33%
397.994
3.37%
478.017
0.53%
m/z (Da)

Compound 6

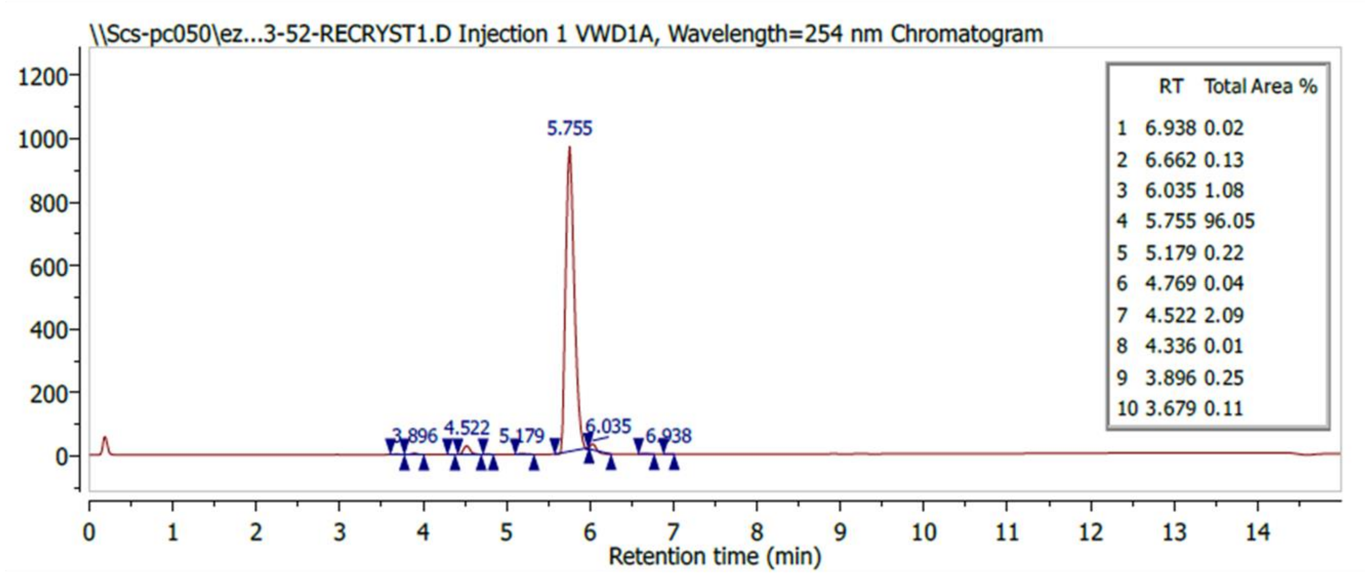
\\Scs-pc050\ez...3-52-RECRYST1.D Injection 1 VWD1A, Wavelength=254 nm Chromatogram
RT Total Area %
1 6.938 0.02
2 6.662 0.13
3 6.035 1.08
4 5.755 96.05
5 5.179 0.22
6 4.769 0.04
7 4.522 2.09
8 4.336 0.01
9 3.896 0.25
10 3.679 0.11
5.755
3.896
4.522
5.179
6.035
6.938
Retention time (min)

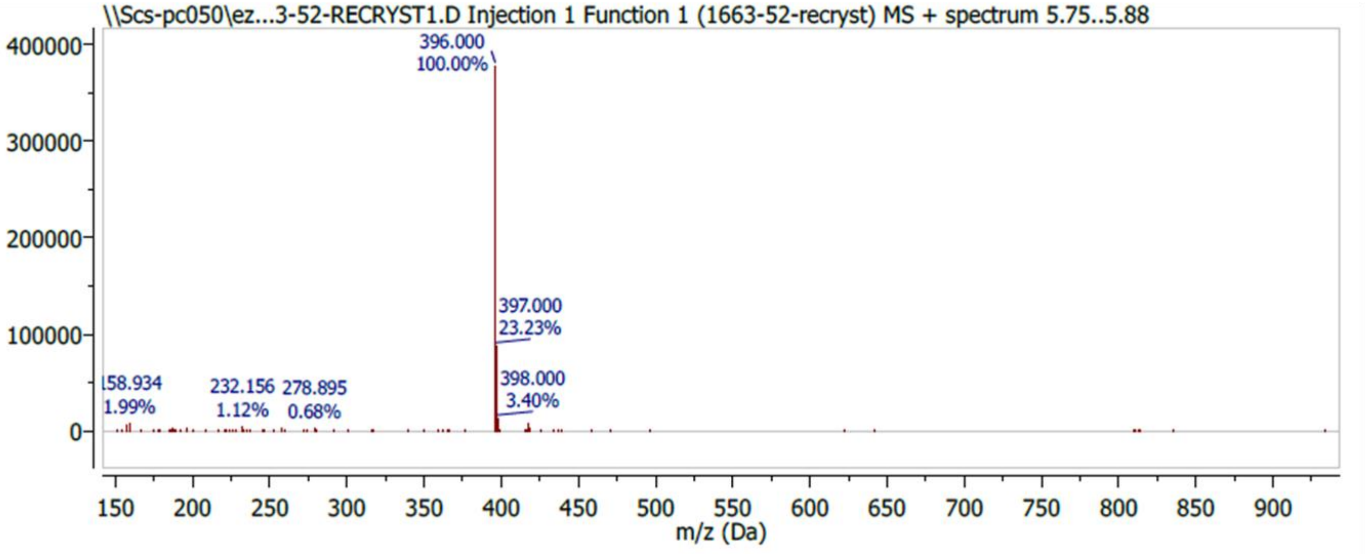
\\Scs-pc050\ez...3-52-RECRYST1.D Injection 1 Function 1 (1663-52-recryst) MS + spectrum 5.75..5.88
396.000
100.00%
397.000
23.23%
398.000
3.40%
158.934
1.99%
232.156
1.12%
278.895
0.68%
m/z (Da)

Compound 7

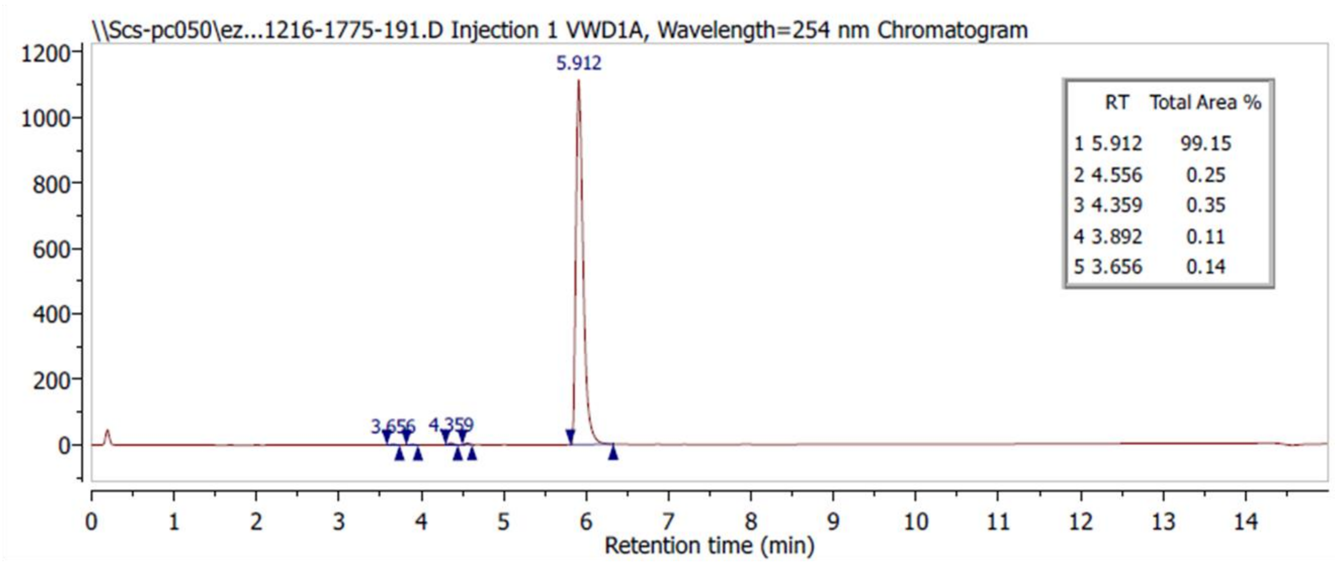

\\Scs-pc050\ez...1216-1775-191.D Injection 1 VWD1A, Wavelength=254 nm Chromatogram
5.912
3.656
4.359
RT Total Area %
1 5.912 99.15
2 4.556 0.25
3 4.359 0.35
4 3.892 0.11
5 3.656 0.14
Retention time (min)

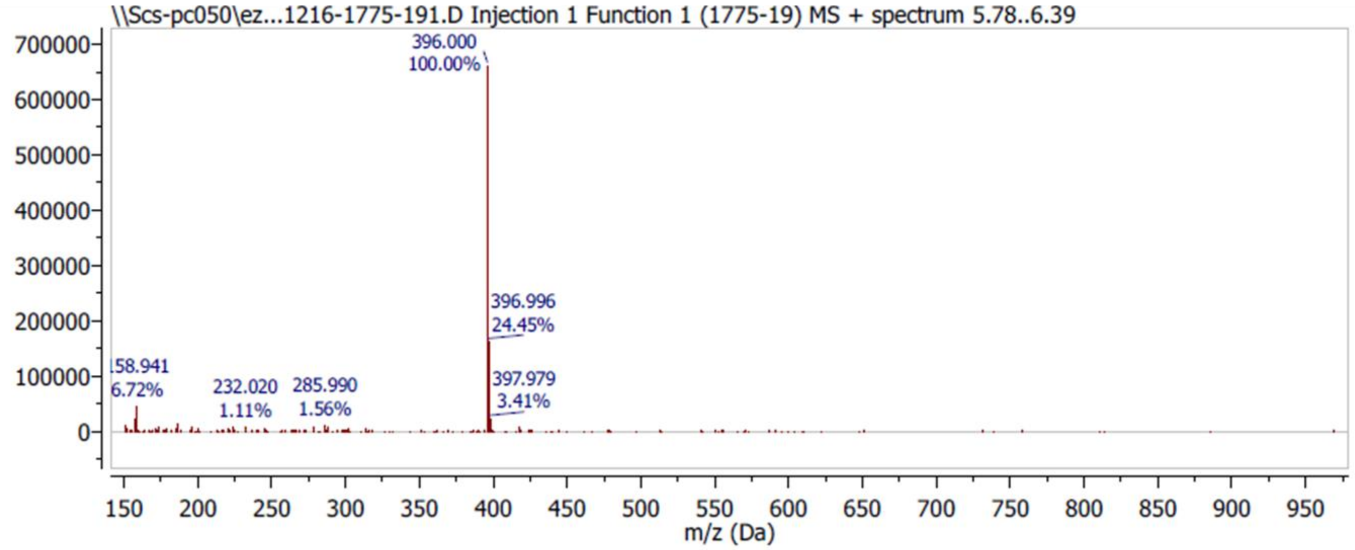

\\Scs-pc050\ez...1216-1775-191.D Injection 1 Function 1 (1775-19) MS + spectrum 5.78..6.39
396.000
100.00%
396.996
24.45%
397.979
3.41%
158.941
6.72%
232.020
1.11%
285.990
1.56%
m/z (Da)

Compound 8

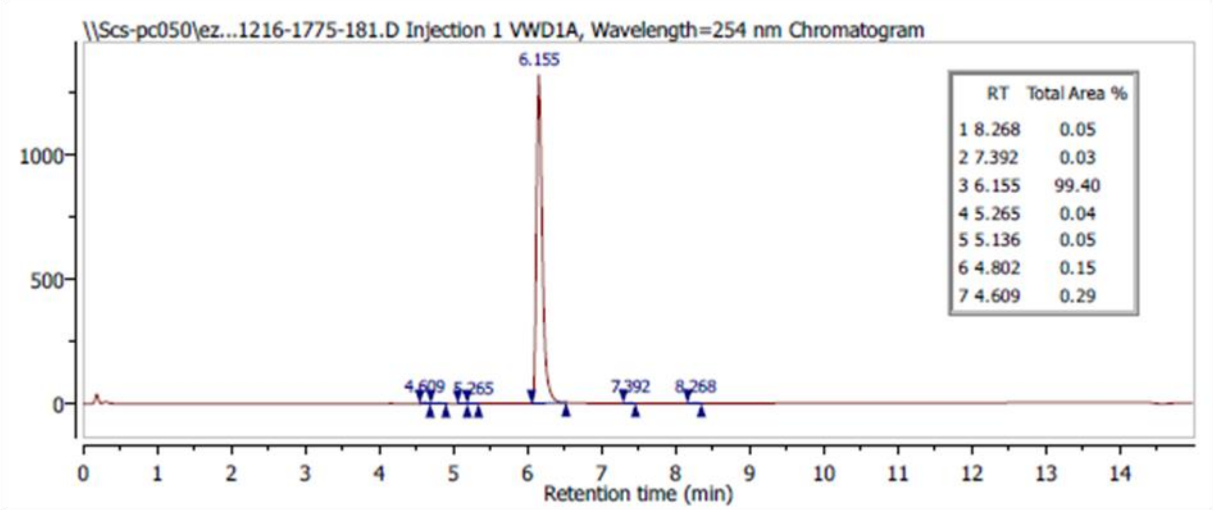
\\Scs-pc050\ez...1216-1775-181.D Injection 1 VWD1A, Wavelength=254 nm Chromatogram
6.155
RT Total Area %
1 8.268 0.05
2 7.392 0.03
3 6.155 99.40
4 5.265 0.04
5 5.136 0.05
6 4.802 0.15
7 4.609 0.29
Retention time (min)

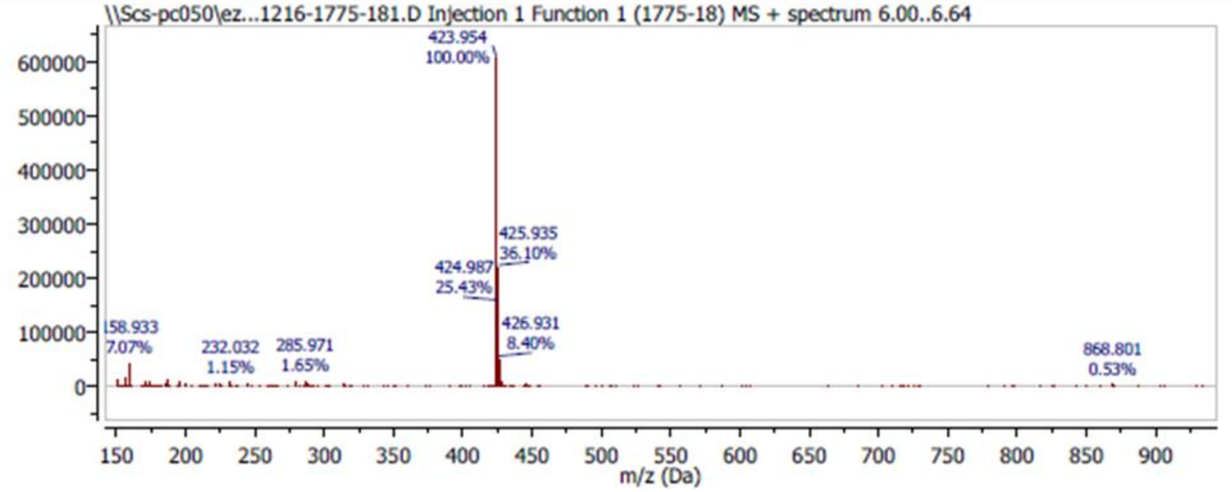
\\Scs-pc050\ez...1216-1775-181.D Injection 1 Function 1 (1775-18) MS + spectrum 6.00..6.64
423.954 100.00%
425.935 36.10%
424.987 25.43%
426.931 8.40%
158.933 7.07%
232.032 1.15%
285.971 1.65%
868.801 0.53%
m/z (Da)

Compound 9

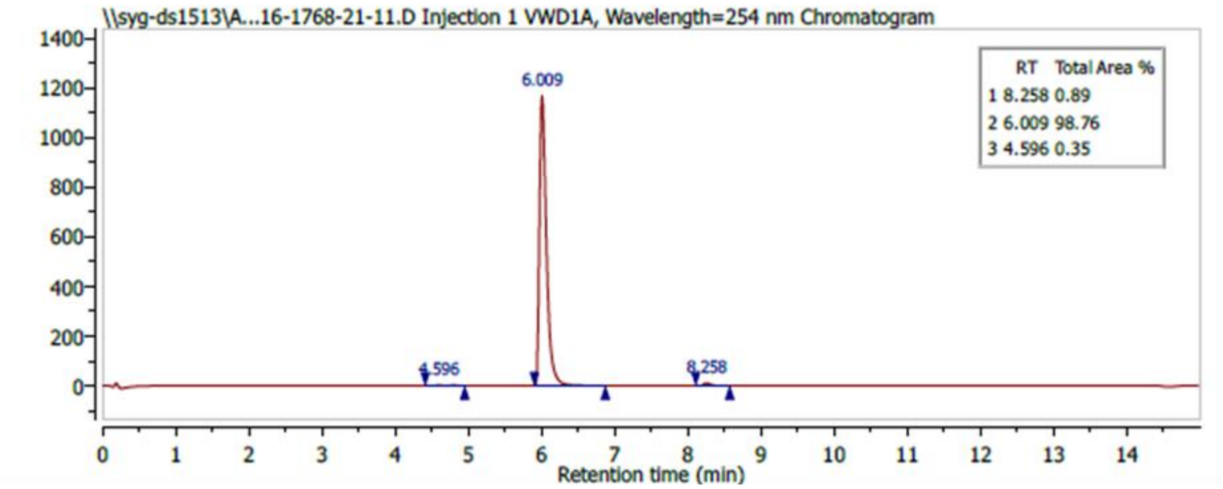
\\syg-ds1513\A...16-1768-21-11.D Injection 1 VWD1A, Wavelength=254 nm Chromatogram
RT Total Area %
1 8.258 0.89
2 6.009 98.76
3 4.596 0.35
6.009
4.596
8.258
Retention time (min)

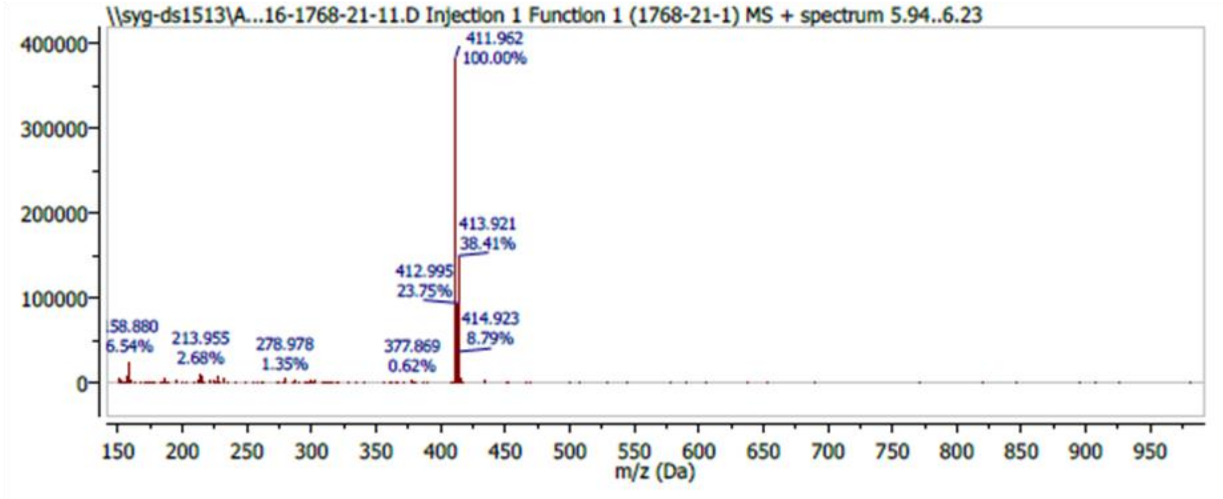
\\syg-ds1513\A...16-1768-21-11.D Injection 1 Function 1 (1768-21-1) MS + spectrum 5.94..6.23
411.962
100.00%
413.921
38.41%
412.995
23.75%
414.923
8.79%
158.880
6.54%
213.955
2.68%
278.978
1.35%
377.869
0.62%
m/z (Da)

Compound 10

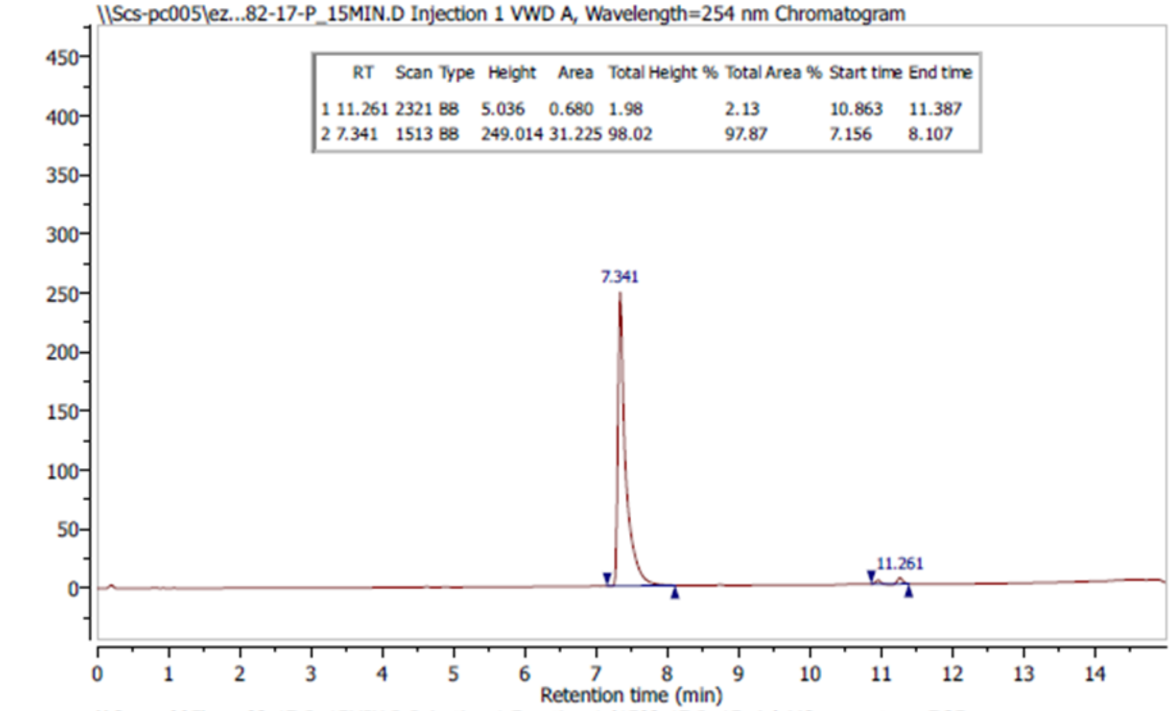
\\Scs-pc005\ez...82-17-P_15MIN.D Injection 1 VWD A, Wavelength=254 nm Chromatogram
RT Scan Type Height Area Total Height % Total Area % Start time End time
1 11.261 2321 BB 5.036 0.680 1.98 2.13 10.863 11.387
2 7.341 1513 BB 249.014 31.225 98.02 97.87 7.156 8.107
7.341
11.261
Retention time (min)

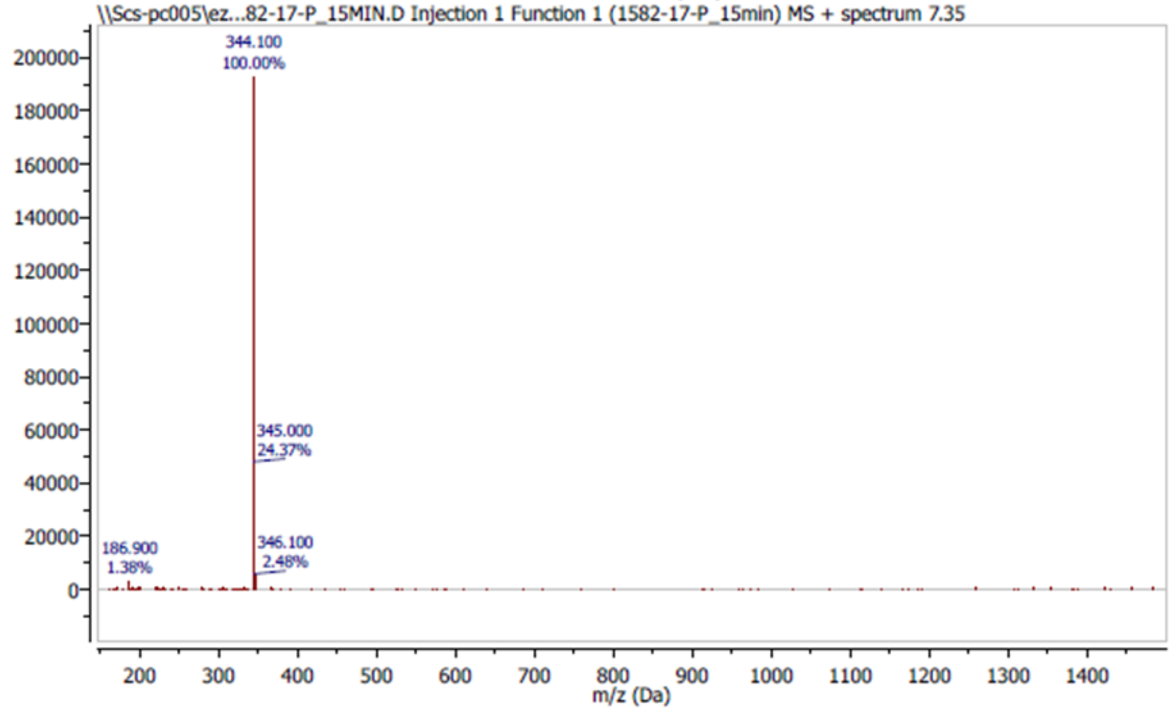
\\Scs-pc005\ez...82-17-P_15MIN.D Injection 1 Function 1 (1582-17-P_15min) MS + spectrum 7.35
344.100
100.00%
345.000
24.37%
346.100
2.48%
186.900
1.38%
m/z (Da)

Compound 11

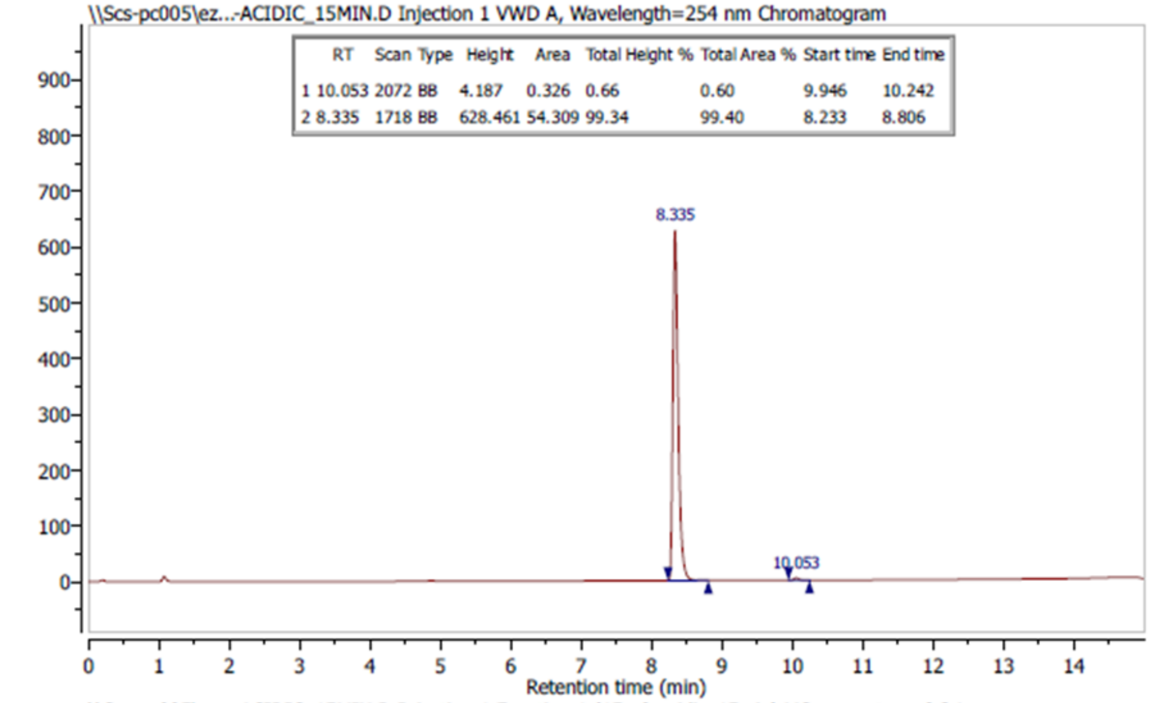

	RT	Scan	Type	Height	Area	Total Height %	Total Area %	Start time	End time
1	10.053	2072	BB	4.187	0.326	0.66	0.60	9.946	10.242
2	8.335	1718	BB	628.461	54.309	99.34	99.40	8.233	8.806

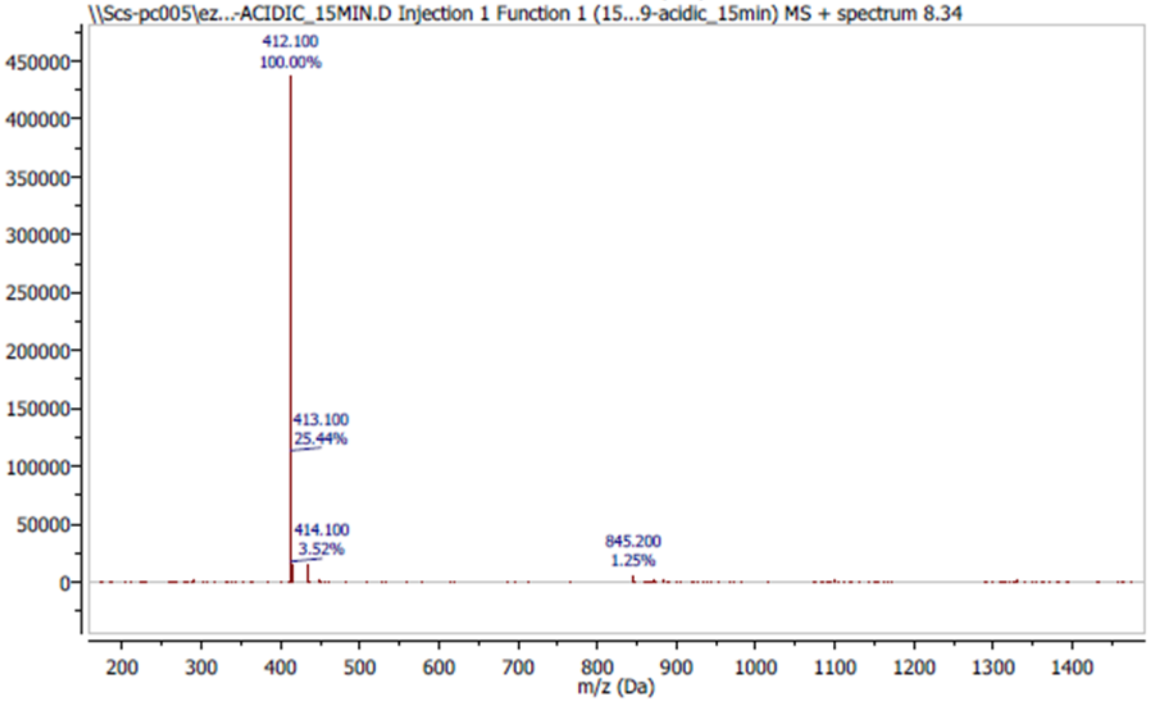

Compound 12

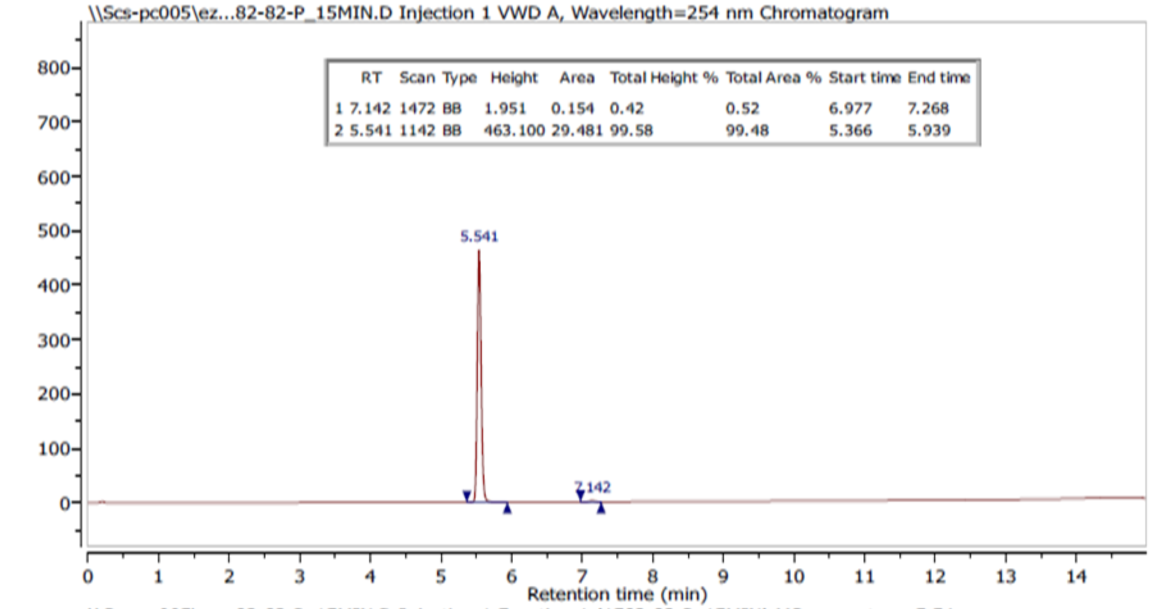
\\Scs-pc005\ez...82-82-P_15MIN.D Injection 1 VWD A, Wavelength=254 nm Chromatogram
RT Scan Type Height Area Total Height % Total Area % Start time End time
1 7.142 1472 BB 1.951 0.154 0.42 0.52 6.977 7.268
2 5.541 1142 BB 463.100 29.481 99.58 99.48 5.366 5.939
5.541
7.142
Retention time (min)

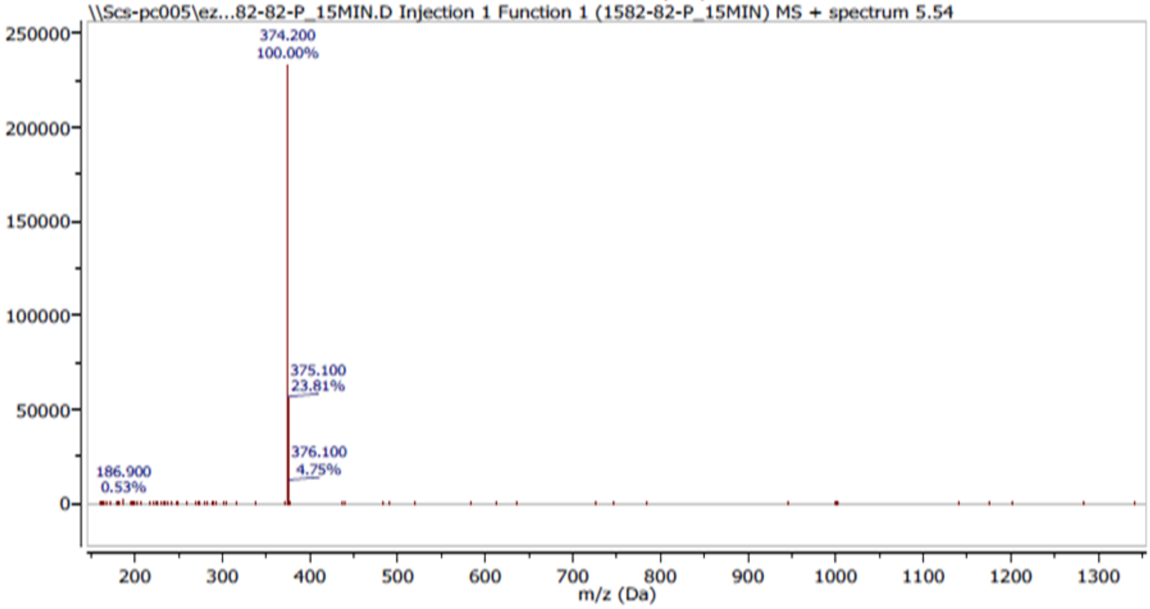
\\Scs-pc005\ez...82-82-P_15MIN.D Injection 1 Function 1 (1582-82-P_15MIN) MS + spectrum 5.54
374.200
100.00%
375.100
23.81%
376.100
4.75%
186.900
0.53%
m/z (Da)

Compound 13

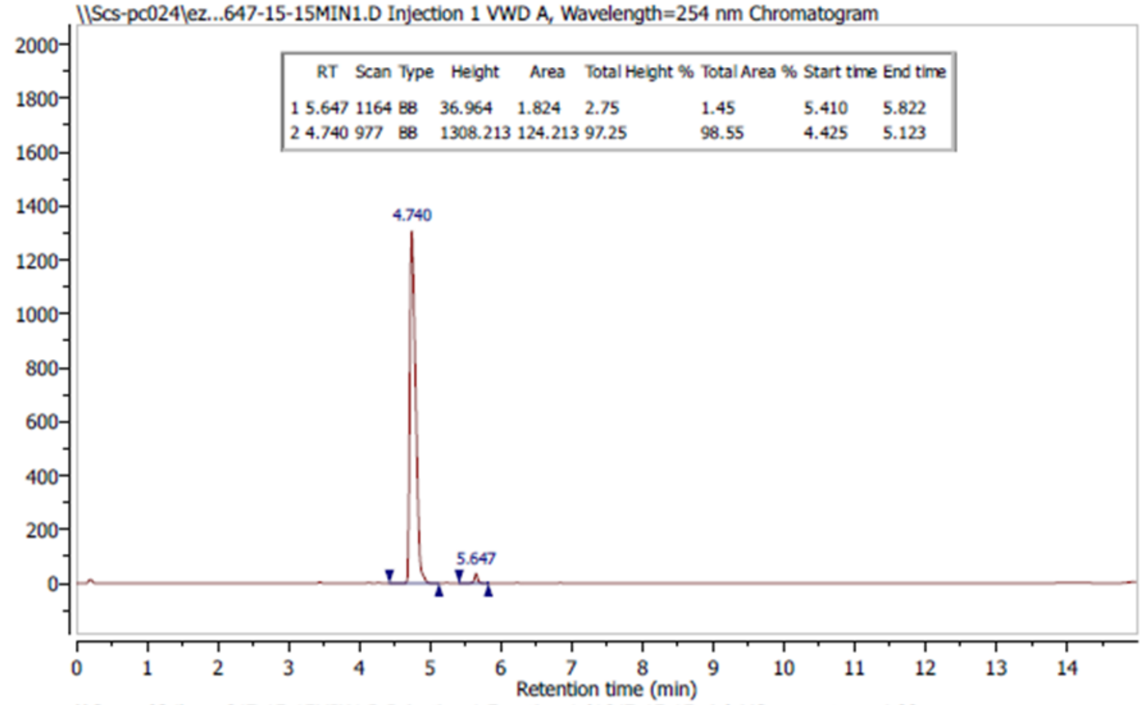

	RT	Scan	Type	Height	Area	Total Height %	Total Area %	Start time	End time
1	5.647	1164	BB	36.964	1.824	2.75	1.45	5.410	5.822
2	4.740	977	BB	1308.213	124.213	97.25	98.55	4.425	5.123

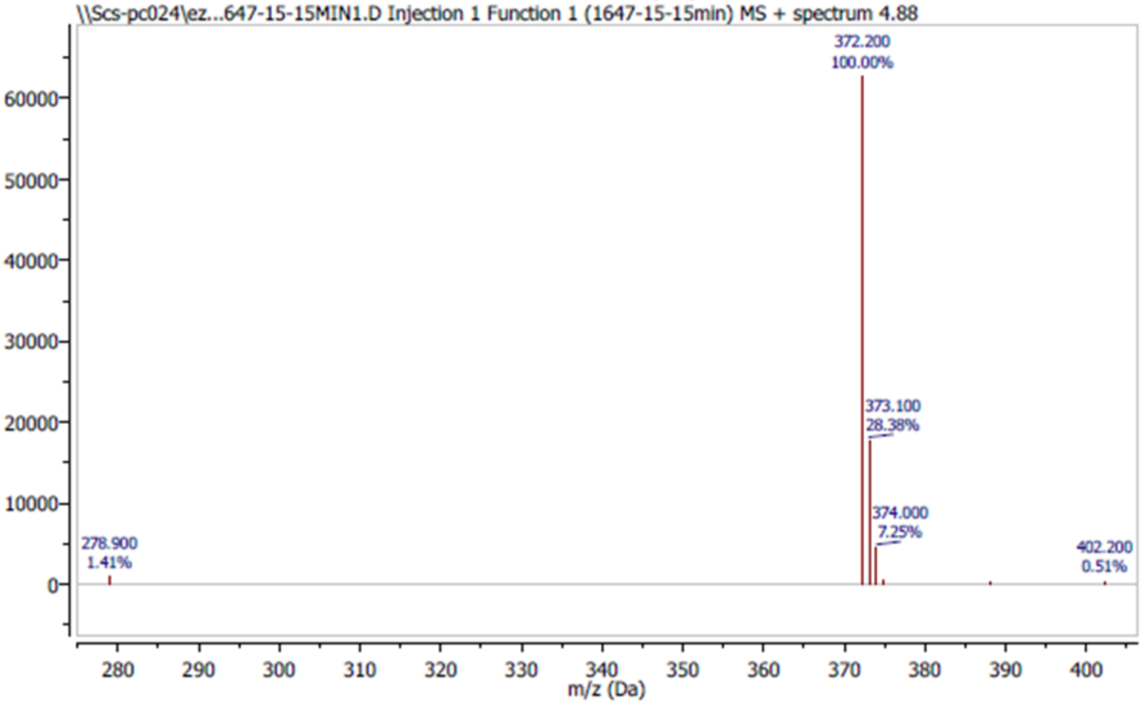

Compound 14

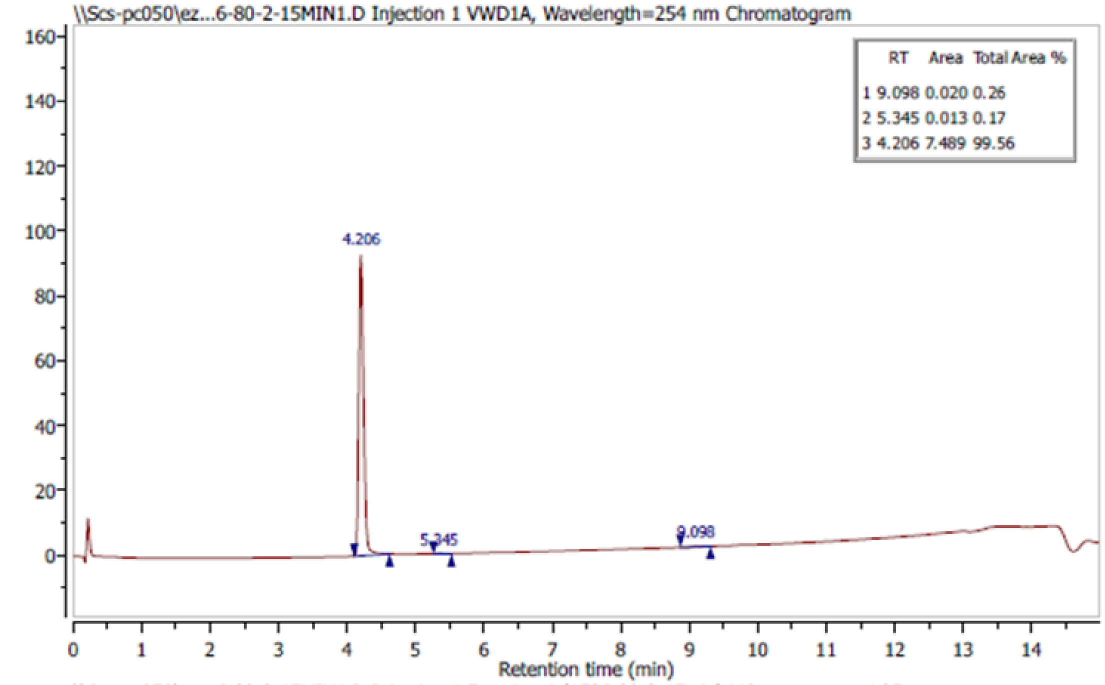

\\Scs-pc050\ez...6-80-2-15MIN1.D Injection 1 VWD1A, Wavelength=254 nm Chromatogram
RT Area Total Area %
1 9.098 0.020 0.26
2 5.345 0.013 0.17
3 4.206 7.489 99.56
4.206
5.345
9.098
Retention time (min)

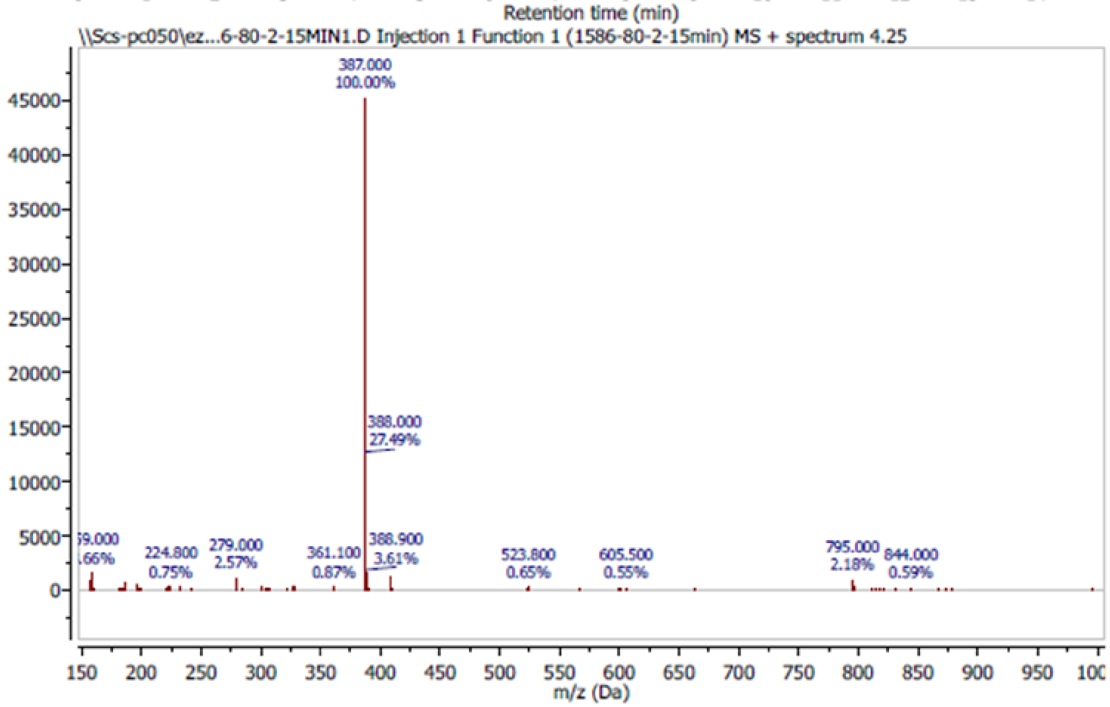

\\Scs-pc050\ez...6-80-2-15MIN1.D Injection 1 Function 1 (1586-80-2-15min) MS + spectrum 4.25
387.000
100.00%
388.000
27.49%
224.800
0.75%
279.000
2.57%
361.100
0.87%
388.900
3.61%
523.800
0.65%
605.500
0.55%
795.000
2.18%
844.000
0.59%
m/z (Da)
